# Vestibular dose predicts toxicity in stereotactic radiosurgery for vestibular schwannomas

**DOI:** 10.1016/j.ctro.2025.101105

**Published:** 2026-01-01

**Authors:** Dimitrios Daskalou, Edouard Romano, Sophie Neveü, Pelagia Tsoutsou, Nikolaos Koutsouvelis, Francis Rousset, Nils Guinand, Minerva Becker, Pascal Senn, Sebastien Tran

**Affiliations:** a Service of Otorhinolaryngology-Head and Neck Surgery, Department of Clinical Neurosciences, Geneva University Hospitals, Geneva, Switzerland; b The Inner Ear and Olfaction Lab, University of Geneva, Faculty of Medicine, Geneva, Switzerland; c Department of Radiation Oncology, University Hospitals of Geneva, Geneva, Switzerland; d Department of Diagnostic Radiology, University Hospitals of Geneva, Geneva, Switzerland

**Keywords:** Vestibular schwannoma, Radiosurgery, Vestibular symptoms, Inner ear, Dosimetric optimization, Vestibular function test

## Abstract

**Introduction:**

Stereotactic radiosurgery (SRS) provides excellent tumor control in small and medium vestibular schwannomas (VS), but its impact on the vestibular system remains uncertain. This study examines the effects of SRS on subjective vestibular symptoms, vestibular function, and potential predictors of symptom worsening.

**Materials and methods:**

A retrospective analysis was conducted on adult VS patients treated with SRS over eight years. Vestibular symptoms were graded at baseline and six months post-SRS. The vestibular sensory organs (VSO) were defined as the combined volume of the saccule, utricle, and ampullae. Vestibular function was assessed with bithermal, bilateral caloric testing, video head impulse testing, and vestibular evoked myogenic potentials. Two illustrative treatment plans were generated to assess feasibility of vestibular dose reduction without compromising planning target volume (PTV) or cochlear constraints.

**Results:**

Among 45 VS patients (median age 61.4 years), 14 (31 %) reported worsened vestibular symptoms at six months. These patients had higher Dose_mean_ to VSO (6.45 Gy vs 2.92 Gy, p < 0.0001). A Dose_mean_ > 4 Gy was strongly associated with symptom worsening (OR = 27.3, 95 % CI [3.4–301.8], p = 0.0002). Similar associations were observed for Dose_max_, with an 8 Gy threshold. Higher Dose_mean_ to the lateral ampulla was correlated with a greater percentage change in caloric weakness (slope = 7.77, R^2^ = 0.38, p = 0.04). In two illustrative plans, VSO optimisation lowered vestibular dose without compromising PTV coverage or cochlear dose.

**Conclusion:**

Higher vestibular radiation dose is strongly associated with worsened vestibular symptoms and possibly with functional decline. Dose reduction to vestibular subunits is feasible without compromising the tumor dose, helping mitigate these effects.

## Highlights

• Vestibular sensory organs can be delineated on high-resolution planning CT.
• Vestibular sensory organs include the vestibule (saccule and utricle) and ampullae.
• Higher vestibular dose predicts symptom worsening and vestibular function loss.
• Dose_mean_ >4 Gy and Dose_max_ >8 Gy to vestibular sensory organs increase symptom risk.

## 1 Introduction

Vestibular schwannomas (VS) are benign tumors that develop from the Schwann cells of the eighth cranial nerve. Although previously considered rare, recent epidemiological studies estimate a lifetime prevalence exceeding 1 in 500 individuals [1]. Stereotactic radiosurgery (SRS) is a key treatment option for small to medium-sized vestibular schwannomas, offering high tumor control rates [2]. However, the inner ear, comprising the cochlea and vestibular system, is at risk of functional loss after SRS treatment [3].

The cochlea has long been recognized as an organ at risk (OAR) and is routinely delineated during SRS planning, with established dose constraints in both fractionated and single-session settings. Dose optimization has been shown to reduce the risk of radiation-induced hearing loss and tinnitus [4], [5]. In contrast, the radiation tolerance of the vestibular system is poorly understood. Yet, up to a third of treated patients complain about new or increased vestibular symptoms, typically at 6 months post-SRS [3], [6], [7]. While some vestibular dysfunction can be attributed to tumor-related effects, SRS itself is also suspected to contribute to this morbidity [8], [9], [10], [11]. Although radiation-induced vestibular symptoms can significantly impair quality of life, few studies have quantitatively explored the relationship between vestibular radiation dose and post-treatment vestibular function [12], [13], [14], [15], [16], [17], [18].

This study aims to evaluate the impact of SRS on both subjective vestibular symptoms and objectively measured vestibular function in patients with unilateral VS. By delineating the three ampullae and the vestibule (containing the saccule and utricle) individually for the first time in this context, we explored whether incorporating these structures into treatment planning might be feasible, and we aimed to assess whether subunit-level radiation exposure correlates with post-treatment vestibular outcomes.

## 2 Materials and methods

### 2.1 Study design

This retrospective analysis included consecutive non-neurofibromatosis type 2 adult patients treated with SRS for a VS over an 8-year period (2014–2021) at a tertiary care facility. This study was approved by the Regional Research Ethics Committee (CCER, Project ID: 2022-01257) and conducted in accordance with the Declaration of Helsinki.

### 2.2 Patient population

We included all adult patients treated with SRS for a unilateral VS at our center during the aforementioned period. Patients were excluded if they had received prior treatment for their VS (surgery or SRS), had a diagnosis of neurofibromatosis type 2, or had a history of middle ear surgery on the affected side.

### 2.3 Intervention parameters

All patients were treated in a single 12 Gy session to the gross tumor volume (no additional margins for the planning target volume [PTV]), prescribed at the 80 % isodose. Treatment was delivered using either a Novalis TX with non-coplanar arcs or a TrueBeam with micro-MLC via the Hyperarc technique.

### 2.4 Selection of vestibular subunits

**Fig. 1:**
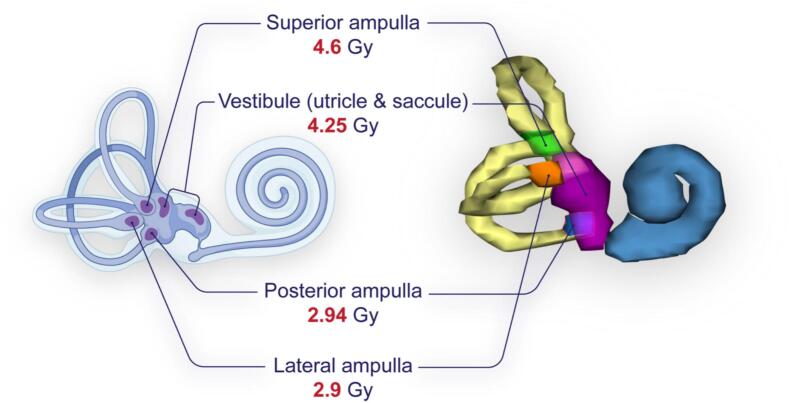
On the left, an anatomical illustration of the inner ear highlighting the superior, lateral, and posterior ampullae and the vestibule (utricle and saccule). On the right, a three-dimensional reconstruction of the delineated vestibular subunits from a planning CT scan, generated using Varian Eclipse software. Corresponding subunits are matched between the schematic and the reconstruction. Values in red represent the median Dmean for each subunit (n = 45). Graphic elements adapted from BioRender.com.

Relevant functional subunits of the vestibular system were defined by ENT specialists and radiation oncologists. The three ampullae (superior, lateral, posterior), mainly detecting angular acceleration, and the utricle and saccule, mainly sensing linear acceleration and gravity, were selected (Fig. 1). Due to their small size and proximity on CT scans, the utricle and saccule were contoured together as a single unit, referred to as the vestibule. The term vestibular sensory organs (VSO) was defined as the combined volume of the vestibule and ampullae.

### 2.5 Vestibular structure delineation

We retrospectively delineated the vestibular apparatus of all patients on the planning CT scan. Delineation on MRI was tested and not retained due to limitations in image registration accuracy and to improve external reproducibility. In collaboration with expert head and neck radiologists, we developed a precise delineation method and an atlas for the vestibule and three ampullae (Supplementary Material 1). The vestibule was contiguous with the ampullae, semicircular canals, and anterior basal cochlea. Planning CT scans had a slice thickness of 0.6–1 mm, and high-resolution contouring tools in Varian Eclipse software were used. This approach allowed clear visualization of the bony labyrinth openings in the petrous temporal bone. Each ampulla was delineated on at least two consecutive slices for dosimetric calculations.

### 2.6 Dosimetric evaluation

We evaluated dosimetry on treatment plans, reporting mean (Dmean) doses for each of the four vestibular subunits and their combined volume (VSO). Due to the small size of the ampullae, maximum dose (Dmax) was reported for the VSO only. Dmax was defined as the near-maximum dose D2% [19]. Additionally, to explore whether the dose to vestibular subunits could be reduced without compromising PTV coverage or increasing cochlear dose, we retrospectively generated dosimetric plans, with and without optimization of vestibular subunits, for two illustrative cases. Planning was first done without optimizing vestibular subunits, aiming to minimize cochlear dose while maintaining 99 % PTV coverage. A second plan then included the VSO in optimization to reduce their dose without altering cochlear dose or PTV coverage. A maximum 5 % variation in cochlear Dmean was allowed, and PTV coverage remained unchanged.

### 2.7 Outcome assessment

#### 2.7.1 Subjective vestibular symptoms

Vestibular symptoms were graded according to the Common Terminology Criteria for Adverse Events (CTCAE) v5.0 (range, 0 to 3) by expert radiation oncologists at baseline and 6 months post-SRS [20]. The items “Vertigo” and “Vestibular disorder” were evaluated; when both applied, the highest grade was used. Vestibular symptoms worsening was defined as an increase of ≥1 grade.

#### 2.7.2 Objective vestibular function

Vestibular function was assessed using a standardized battery comprising bithermal caloric testing, video head impulse testing (vHIT), and ocular and cervical vestibular evoked myogenic potentials (oVEMP and cVEMP).

Caloric testing was performed using bithermal irrigation with water at 30 °C and 44 °C. The resulting lateral canal responses were recorded using videonystagmography. Caloric asymmetry was calculated using Jongkees’ formula, with higher percentages indicating greater unilateral weakness [21]. Absolute slow-phase velocity values were additionally verified. The outcome measure for caloric testing was the shift in caloric weakness from baseline to follow-up.

vHIT (Synapsys, Marseille, France) was used to evaluate the vestibulo-ocular reflex (VOR) gain of the superior, lateral, and posterior semicircular canals. Eye and head velocities were recorded using high-speed infrared video goggles, and canal-specific VOR gain values were extracted. The outcome measure for vHIT was the change in VOR gain from baseline to follow-up.

oVEMP and cVEMP system tests were used to assess the function of the utricle and saccule, respectively. Bone and air conducted oVEMPs, and air conducted cVEMPs were recorded. Responses were classified as present or absent.

To study the progression of vestibular function post-SRS, we included patients with initially normal or mildly affected baseline vestibular function for each respective test. For caloric testing, we included patients with an initial caloric asymmetry <50 %. Regarding vHIT, we accepted a baseline VOR gain >60 % and for o and cVEMPs, a present response on initial evaluation.

### 2.8 Statistical analysis

Descriptive statistics were used to summarize patient characteristics and dosimetric variables. Continuous variables were reported as medians with interquartile ranges (IQR) and compared using the Mann–Whitney *U* test for two-group comparisons or the Kruskal–Wallis test for comparisons involving more than two groups. Categorical variables were analyzed using Fisher’s exact test. Univariable analyses were conducted to identify potential predictors of vestibular symptom worsening. Correlations between continuous variables were assessed using linear regression models. A multivariable linear regression was performed to evaluate the combined effect of tumor volume and distance to the internal auditory canal (IAC) fundus on vestibular subunit dose. Odds ratios (OR) with 95 % confidence intervals (CI) were calculated to assess the association between vestibular dose thresholds and the likelihood of symptom worsening. A two-sided p-value < 0.05 was considered statistically significant. All analyses were performed using GraphPad Prism, version 10.4.2 (build 534).

Our dataset was complete for all dosimetric variables, vestibular schwannoma MRI characteristics, subjective vestibular symptom assessments, and baseline hearing tests. Missing data were present for the baseline vestibular battery (n = 4) and the follow-up vestibular battery (n = 23). Missing data were assumed to be missing at random and were addressed by performing complete case analysis, excluding individuals with incomplete data from each respective statistical test.

## 3 Results

### 3.1 Population and dosimetric distribution across vestibular subunits

A total of 45 patients were included. Patient and treatment characteristics are presented in Table 1. Among the delineated vestibular subunits, the superior ampulla received the highest median Dmean, at 4.6 Gy (IQR, 4.36 Gy), followed by the vestibule (4.25 Gy, IQR 4.8), the posterior ampulla (2.94 Gy, IQR 3.41), and the lateral ampulla (2.9 Gy, IQR 2.79; Fig. 1).

**Table 1:** Patient and treatment characteristics (n = 45).

Age, **median (IQR), yr**	**61.4 (15.45)**
Gender	
Male, n (%)	22 (48.9)
Female, n (%)	23 (51.1)
Tumor side	
Right, n (%)	21 (46.7)
Left, n (%)	24 (53.3)
Tumor volume, median (IQR), mm^3^	410 (233-1050)
Koos grade	
Koos I, n (%)	7 (15.6)
Koos II, n (%)	23 (51.1)
Koos III, n (%)	14 (31.1)
Koos IV, n (%)	1 (2.2)
Distance to fundus, median (IQR), mm	0.5 (0-3)
Treatment device	
Novalis TX, n (%)	39 (86.7)
TrueBeam, n (%)	6 (13.3)

IQR interquartile range.

As expected, a negative correlation was observed between Dmean for each anatomical subunit and the tumor’s distance to the IAC fundus, with higher radiation doses observed in tumors located closer to the fundus across all anatomical regions (Table 2). A negative correlation was also found between Dmean for each subunit and VS volume. However, when combining both the distance to the IAC fundus and VS volume in a multivariable linear regression model, only the first variable predicted vestibular subunit Dmean (Table 2).

**Table 2:** Simple and multiple linear regression predicting dmean to vestibular subunits.

		Simple linear regression		Multivariable linear regression		
Anatomical Subunit	**Predictor**	**β (95 % CI)**	**p-value**	**β (95 % CI)**	**p-value**	**Adjusted R^2^**
Vestibule	Distance to Fundus	−0.56 (−0.7 to −0.4)	<0.0001	−1.2 (−1.6 to −0.8)	<0.0001	0.67
	VS Volume	−123.8 (−212 to – 35)	0.008	0.0002 (−0.0006 to 0.001)	0.53	
Superior Ampulla	Distance to Fundus	−0.48 (−0.7 to −0.3)	<0.0001	−1.3 (−1.8 to −0.8)	<0.0001	0.58
	VS Volume	−101.6 (−192 to −10)	0.03	0.0005 (−0.0005 to 0.001)	0.31	
Lateral Ampulla	Distance to Fundus	−0.71 (−0.9 to −0.4)	<0.0001	−0.83 (−1.2 to −0.5)	<0.0001	0.56
	VS Volume	−135.4 (−275 to 4)	0.06	0.0004 (−0.0003 to 0.001)	0.22	
Posterior Ampulla	Distance to Fundus	−0.75 (−1 to −0.5)	<0.0001	−0.85 (−1.1 to −0.5)	<0.0001	0.6
	VS Volume	−128 (−255 to −0.9)	0.049	0.0004 (−0.0003 to 0.001)	0.26	

CI confidence interval, VS vestibular schwannoma.

### 3.2 Association between vestibular dose and subjective symptoms

**Fig. 2:**
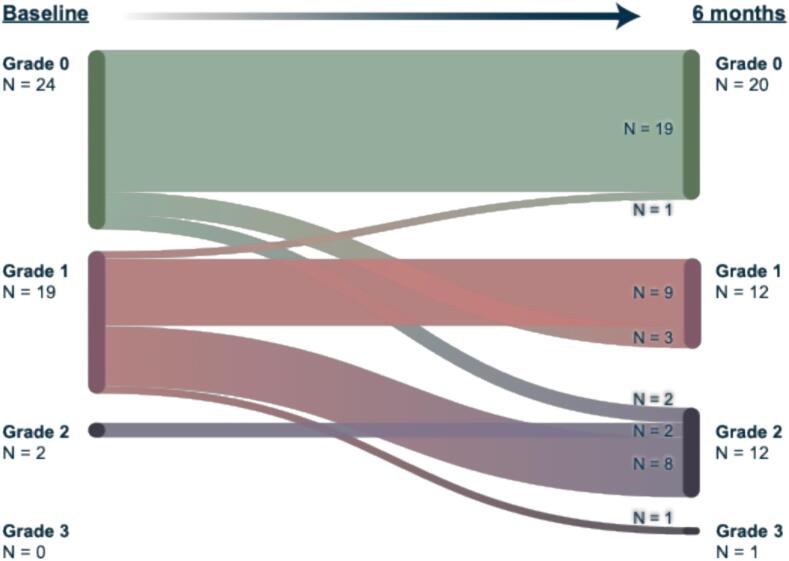
**Alluvial diagram illustrating the evolution of vestibular symptom grades from baseline to 6 months after SRS.** Each flow represents individual patient transitions according to CTCAE v5.0 items “Vertigo” and “Vestibular disorder” (grade range: 0–3). At 6 months, five patients with no baseline symptoms (grade 0) developed new symptoms (three progressed to grade 1 and two to grade 2). Among patients with mild baseline symptoms (grade 1), nine progressed to higher grades (eight to grade 2 and one to grade 3). Overall, 14 of 45 patients (31 %) experienced worsening, 30 patients (67 %) remained stable, and one patient (2 %) improved following SRS.

**Fig. 3:**
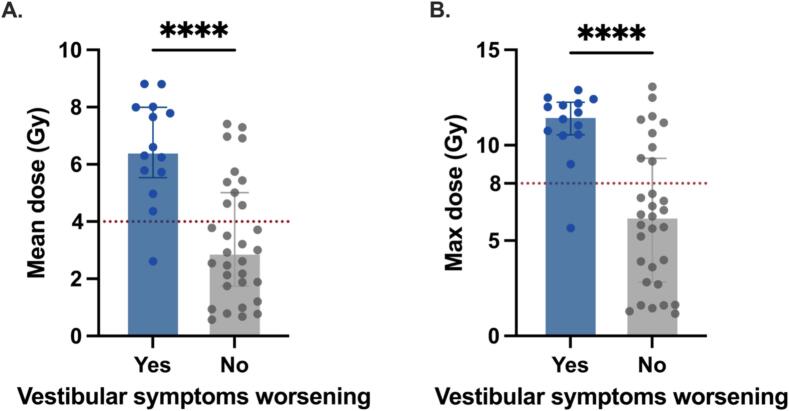
Barplots comparing the (A) mean dose and (B) max dose received by the vestibular sensory organs (combined vestibule and three ampullae) between patients presenting worsened vestibular symptoms at 6 months after stereotactic radiosurgery, compared to those with stable or improved symptoms (n = 45). Mann–Whitney tests were implemented. A significant difference between the two groups is indicated by asterisks (****p < 0.0001). The horizontal red dotted line represents the threshold above which the likelihood of symptom worsening is significantly increased. The height of the boxes represents the median; error bars indicate the interquartile range (Q1–Q3).

At 6-month follow-up, five patients with no baseline vestibular symptoms developed new symptoms (n = 3 grade 1 and n = 2 grade 2). In addition, nine patients with mild baseline symptoms (grade 1) progressed to higher grades (n = 8 grade 2 and n = 1 grade 3; Fig. 2). Together, 14 of the 45 patients (31 %) reported worsened vestibular symptoms following SRS. The VSO (combined vestibule and three ampullae) received significantly higher radiation exposure in these patients compared to those with stable or improved symptoms (median Dmean 6.45 Gy vs. 2.92 Gy, p < 0.0001; Fig. 3A). A Dmean > 4 Gy was significantly associated with an increased likelihood of symptom worsening (OR = 27.3; 95 % CI, 3.4–301.8; p = 0.0002; Fig. 3A).

Similarly, patients with symptom worsening had a significantly higher median Dmax to the VSO (11.55 Gy vs. 6.27 Gy; p < 0.0001; Fig. 3B). A Dmax > 8 Gy was also highly associated with increased risk (OR = 31.8; 95 % CI 3.9–350.4; p < 0.0001; Fig. 3B).

**Fig. 4:**
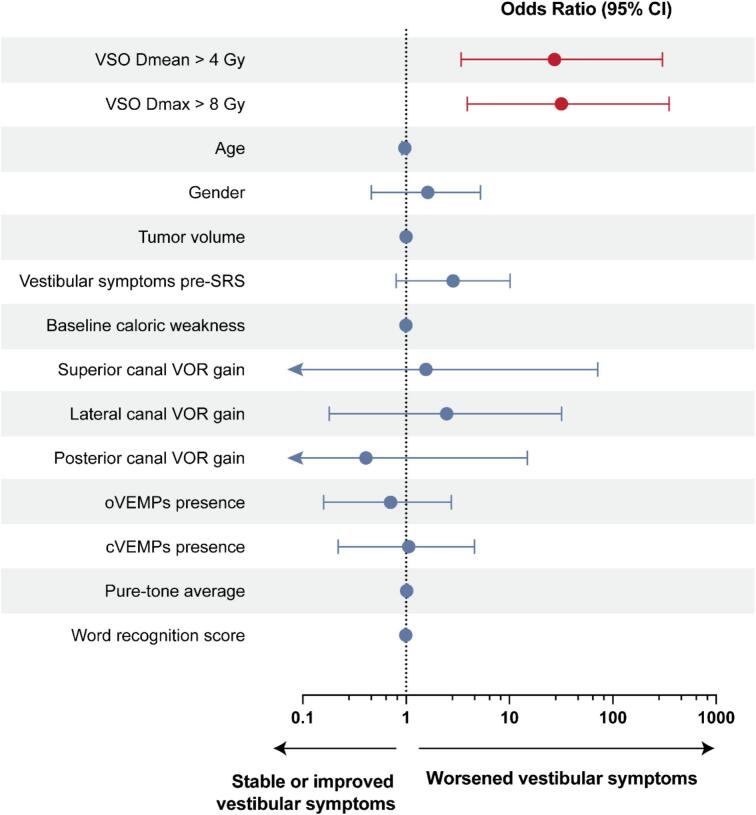
**Predictors of vestibular symptom worsening at 6 months after stereotactic radiosurgery.** Forest plot of odds ratios with 95 % confidence intervals (CI) from univariable logistic regression (N = 45; 14 worsened). Dose metrics refer to the vestibular sensory organs (VSO; vestibule and three ampullae), dichotomized at Dmean > 4 Gy and Dmax > 8 Gy; both were significantly associated with symptom worsening. Other variables were not significant. The vertical dotted line indicates OR = 1 (left, lower odds; right, higher odds). VSO vestibular sensory organs, Dmean mean dose, Dmax maximum dose, VOR vestibulo-ocular reflex, oVEMPs ocular vestibular evoked myogenic potential, cVEMPs cervical vestibular evoked myogenic potential, SRS stereotactic radiosurgery.

We searched for potential confounders that could contribute to this striking association. Demographic factors, including age (p = 0.4) and gender (p = 0.5), as well as tumor characteristics such as tumor volume (p = 0.4) and Koos grade (p = 0.9), were not associated with subjective vestibular worsening. Similarly, the presence of vestibular symptoms prior to SRS (p = 0.2) did not predict post-treatment symptom exacerbation. Baseline vestibular function, as assessed by caloric weakness (p = 0.3), VOR gain for the superior (p = 0.7), lateral (p = 0.4), and posterior (p = 0.6) semicircular canals, oVEMPs (p = 0.7), and cVEMPs (p > 0.99), was not significantly associated with subjective outcome at 6 months. Baseline hearing function, measured by pure-tone average (p = 0.6) and word recognition score (p = 0.5), also did not predict vestibular symptom worsening (Fig. 4).

### 3.3 Association between vestibular subunit dose and measured vestibular function

**Fig. 5:**
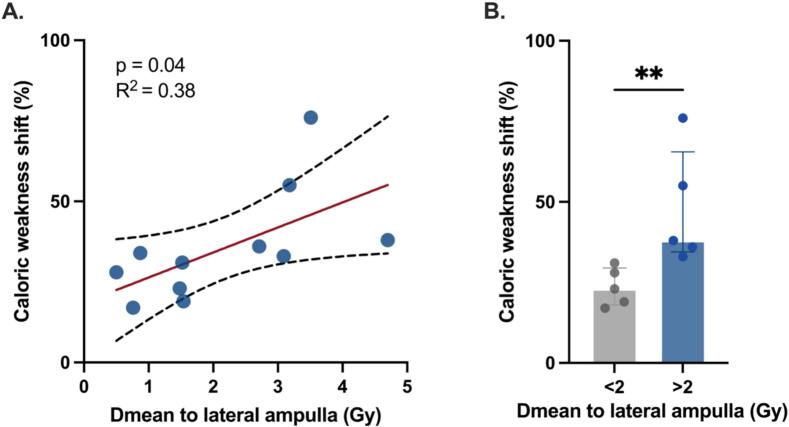
A. A scatter plot demonstrating the relationship between caloric weakness shift from baseline to post-radiosurgery evaluation and the Dmean received by the lateral ampulla (n = 11). A positive linear correlation was observed (R^2^ = 0.38; p = 0.04). The red line indicates the linear regression fit, and the dashed lines indicate the 95 % confidence interval. B. Barplots comparing the caloric weakness shift between patients who received a Dmean < 2 Gy to the lateral ampulla and those who received a Dmean > 2 Gy. A Mann–Whitney test was implemented. A significant difference between the two groups is indicated by asterisks (**p = 0.008). The height of the boxes represents the median; error bars indicate the interquartile range (Q1–Q3). Dmean mean dose.

A subgroup of 11 patients met the inclusion criteria for analysis of measured vestibular function using bithermal caloric testing. The median follow-up time was 15 months (IQR, 15), and no correlation was found between the time interval from baseline to follow-up testing and vestibular outcome (p = 0.8). A significant positive correlation was observed between the percentage shift in caloric weakness and the Dmean to the lateral ampulla (slope = 7.77; 95 % CI [0.28–15.26]; R^2^ = 0.38; p = 0.04; Fig. 5A). Patients who received a Dmean > 2 Gy to the lateral ampulla exhibited a significantly greater percentage shift in caloric weakness compared to patients receiving < 2 Gy (median shift 38 % vs. 23 %; p = 0.008; Fig. 5B). Among these patients, three (27 %) reported subjective worsening of vestibular symptoms at 6 months. These individuals exhibited a greater percentage shift in caloric response (median 55 % vs. 30.5 %), although this difference did not reach statistical significance (p = 0.13).

**Fig. 6:**
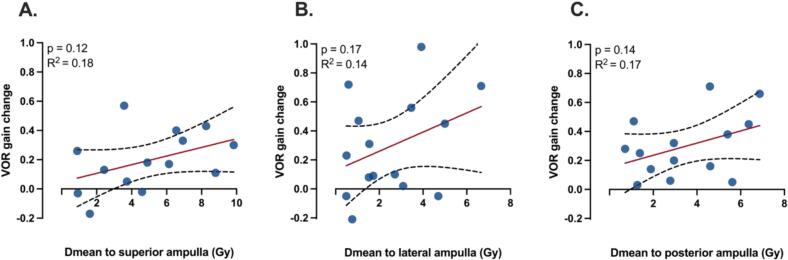
Scatter plots showing the relationship between VOR gain change and the Dmean received by the (A) superior (n = 14), (B) lateral (n = 15), and (C) posterior (n = 14) semicircular canals. A trend for a positive correlation is observed, albeit non-significant. The red line indicates the linear regression fit, and the dashed lines indicate the 95 % confidence interval. VOR vestibulo-ocular reflex, Dmean mean dose.

Changes in VOR gain on vHIT for the superior (n = 14), lateral (n = 15), and posterior (n = 14) semicircular canals demonstrated a trend toward a positive correlation with the Dmean to the respective ampullae; however, none of these associations reached statistical significance (Fig. 6.A-C).

The Dmean received by the vestibule (utricle and saccule) did not differ between patients who lost oVEMP (p = 0.45) or cVEMP (p = 0.9) responses compared to those who maintained these responses post-SRS.

### 3.4 Dosimetric optimization aimed at reducing vestibular dose

To illustrate the technical feasibility of reducing vestibular dose, we generated two treatment plans for two illustrative Koos II tumors in which the VSO were added as optimization objectives. In the first case, VSO Dmean and Dmax decreased by 33 % and 27 %; in the second, by 46 % and 25 %, respectively, with identical PTV coverage and cochlear dose in both plans. As this exploratory analysis is limited to two examples, it is presented solely to demonstrate feasibility.

## 4 Discussion

Our study highlights the potential benefit of introducing the vestibular sensory organs as a new organ at risk during planning of stereotactic radiosurgery for vestibular schwannoma. We demonstrated that these structures can be reliably contoured on CT with millimetric slices, and in two illustrative planning examples, incorporating the VSO into optimization did not compromise PTV coverage and cochlear dose. Clinically, we found that one-third of patients reported worsened vestibular symptoms six months after treatment, and that higher doses to the VSO were significantly associated with this outcome. Furthermore, we observed a dose–response relationship between radiation dose to the lateral ampulla and changes in caloric function, supporting the relevance of subunit-level dose analysis.

In the present study, 31 % of our population reported worsened vestibular symptoms at 6 months post-SRS. This finding aligns with a previous large study involving 500 patients treated with Gamma Knife radiosurgery [6]. We found that Dmean > 4 Gy and Dmax > 8 Gy delivered to the VSO were strongly associated with worsening vestibular symptoms at 6 months. This matches previous work by Tuleasca and colleagues, who studied the dose delivered to the vestibule, primarily represented by the utricle, as a surrogate for the posterior labyrinth, and showed that a maximum dose of more than 8 Gy was linked to new vestibular symptoms within the first 6 months after SRS [22]. Stavas et al. studied 10 patients and found no association between radiation dose and changes in vestibular function or DHI scores. Several methodological differences could explain this discrepancy [18]. First, most (9/10) patients in their study underwent fractionated radiosurgery, whereas our study used single-session SRS. Second, they included patients with poor baseline vestibular function (median caloric weakness of 65 %). Third, they attempted to correlate the vestibule dose with caloric testing, which mainly reflects lateral semicircular canal function. Considering the significant dose difference between the vestibule and lateral ampulla (median Dmean 4.25 Gy vs. 2.9 Gy in our study), this may explain their negative findings. In a recent retrospective study, Ermiş et al. measured the dose to the vestibule and found a dose–response relationship with patient-reported dizziness at 6 months post-SRS [14]. Notably, they report that a minimum dose (Dmin) > 5 Gy is linked to worsened vestibular symptoms. Given the challenges of using Dmin in current practice, our study focused on Dmean and Dmax, finding clear relationships with cut-offs at 4 and 8 Gy, respectively. Interestingly, these cut-offs are similar to those used clinically for the cochlea [23], [24], [25]. Ermiş and colleagues also noted that improved caloric function post-SRS is associated with lower Dmean and Dmax at the vestibule. Our findings support these results and provide additional detail by correlating the caloric response with the dose received by the lateral ampulla. Furthermore, our analysis included dosimetric assessments of the ampullae and examined the full dynamic range of the VSO, including the vHIT, which was absent in previous studies.

The present study is the first to evaluate vestibular subunits separately in the context of SRS. These subunits provide the central nervous system with motion information and play a key role in all domains of the vestibular system and balance [26]. Importantly, they can each be assessed using dedicated vestibular tests, making a subunit-specific analysis both feasible and functionally relevant for research purposes. Although our study evaluated the subunits separately to correlate them with objective vestibular function and assess the value of contouring them, only the VSO was incorporated into treatment planning in clinical practice, applying the soft constraints described above (Dmax < 8 Gy and Dmean < 4 Gy). In our cohort, a significant association was observed between the dose to the lateral ampulla and changes in caloric function, a test that primarily reflects the function of the lateral semicircular canal [27]. In addition, we observed a consistent trend between reduced VOR gain on the vHIT and higher radiation dose to the corresponding ampullae, although these associations did not reach statistical significance. The semicircular canals themselves were not delineated, as they do not contain sensory receptors; only the ampullae, which house the cristae ampullaris responsible for detecting angular acceleration, were considered, given their anatomical and functional relevance.

The present study has several limitations, the first being its retrospective nature and the small size of its cohort. The single-center dosimetric design introduces center bias. Differences in contouring, planning, and accelerator accuracy across institutions may limit reproducibility of the observed benefit, requiring external validation [28]. In addition, the feasibility of vestibular dose reduction was illustrated in only two cases, and these examples should therefore be interpreted as technical demonstrations rather than generalizable dosimetric conclusions. Vestibular symptoms were assessed by experienced radiation oncologists at baseline and at 6 months post-SRS using the CTCAE v5.0 grading criteria. However, a standardized, patient-reported questionnaire was not used, which might have captured more nuanced symptomatology. In addition, vestibular outcomes were assessed at a single post-treatment time point, limiting our ability to evaluate the temporal dynamics of symptom evolution, as early deterioration may be followed by compensation-related improvement. Another limitation is the non-uniform timing of post-SRS vestibular function testing. Although we investigated the relationship between follow-up interval and measured vestibular decline and found no significant association, this variability may still introduce bias. Furthermore, follow-up vestibular testing was available for only a subset of patients, which may have reduced the statistical power to detect associations. These limitations should be considered when interpreting our findings.

Collectively, these findings support the integration of VSO into future SRS planning with the aim of minimizing vestibular toxicity. As functional preservation becomes an increasingly central goal in the management of VS, strategies to spare critical inner ear structures are essential to improving patient quality of life. Our approach provides a framework for selectively reducing dose to vestibular substructures without compromising target coverage or cochlear constraints. Building on these results, we are now conducting a prospective study that incorporates vestibular subunits as OAR during treatment planning, coupled with a standardized, longitudinal assessment of vestibular function using a comprehensive battery of tests. This next step will help validate the clinical utility of our approach and further clarify dose–response relationships.

## 5 Conclusions

Our study demonstrates that incorporating the vestibular sensory organs (including vestibular ampullae and the vestibule) into OAR delineation during SRS planning for VS may be feasible and clinically relevant. Higher radiation doses to these subunits were associated with both subjective symptom worsening and objective vestibular dysfunction. Integrating these structures into treatment planning may help mitigate vestibular toxicity and contribute to improved patient quality of life.

## Funding

No funding was received to assist with the preparation of this manuscript. The Swiss Academy of Medical Sciences MD–PhD grant (323630_214546) supported D.D. The remaining authors disclose no conflicts of interest.

## Declaration of Competing Interest

The authors declare that they have no known competing financial interests or personal relationships that could have appeared to influence the work reported in this paper.
