# Supplementary material for: Vestibular dose predicts toxicity in stereotactic radiosurgery for vestibular schwannomas
Source: Clin Transl Radiat Oncol. 2026 Jan 1;57:101105. doi: 10.1016/j.ctro.2025.101105 (PMC12811486; doi:10.1016/j.ctro.2025.101105)
Supplement: Supplementary Data 1 [file mmc1.pdf]

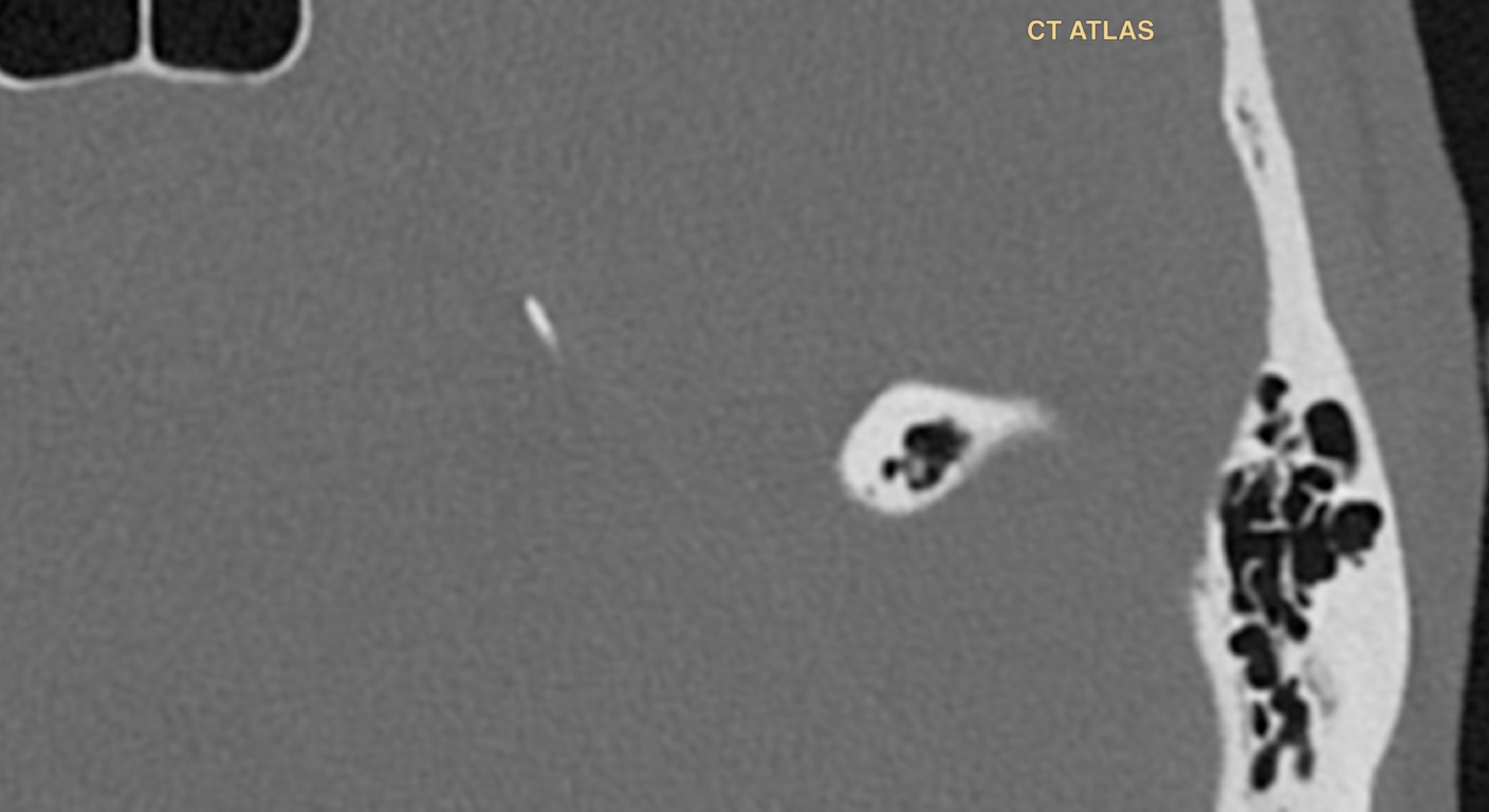
CT ATLAS

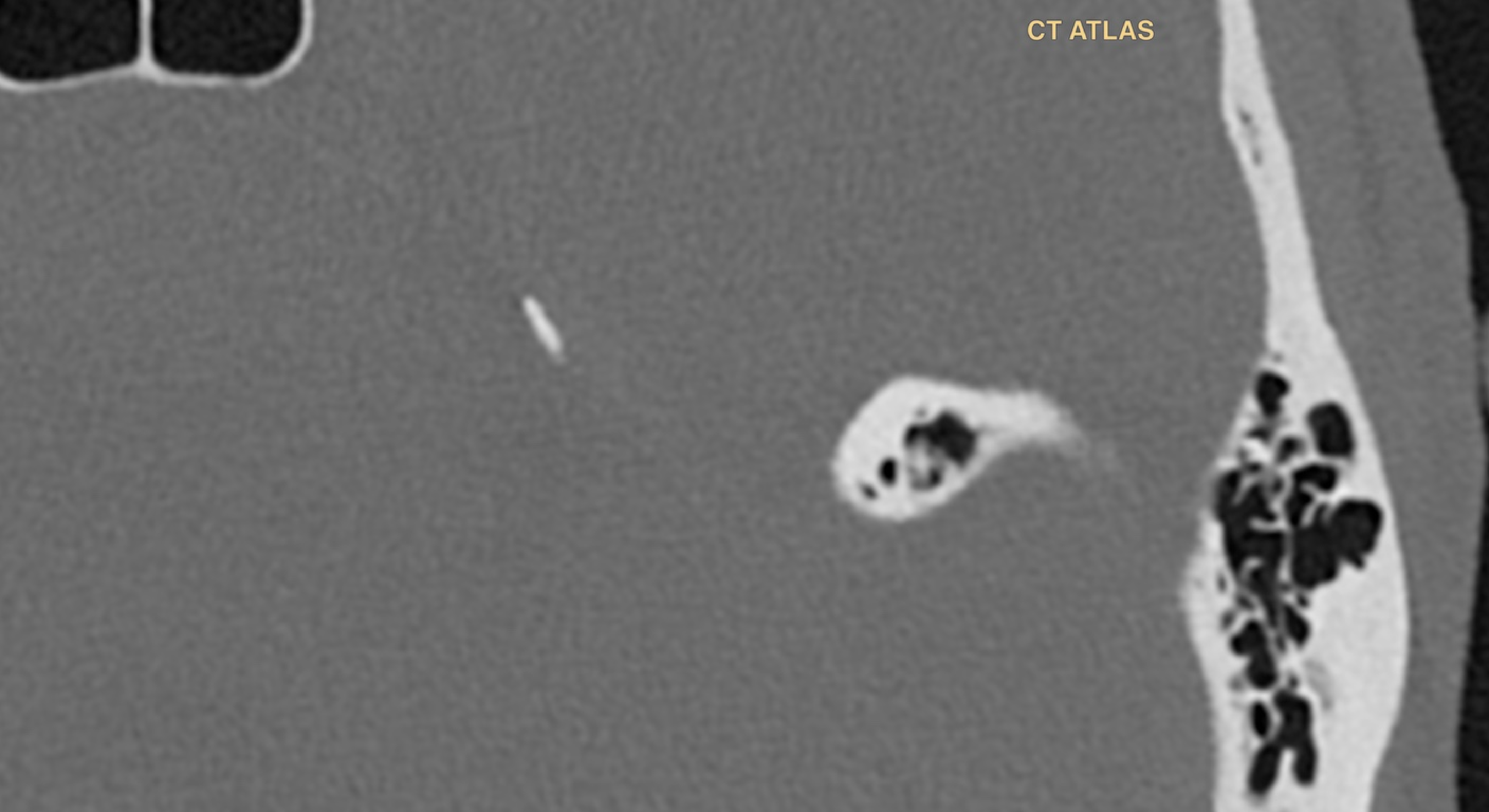
CT ATLAS

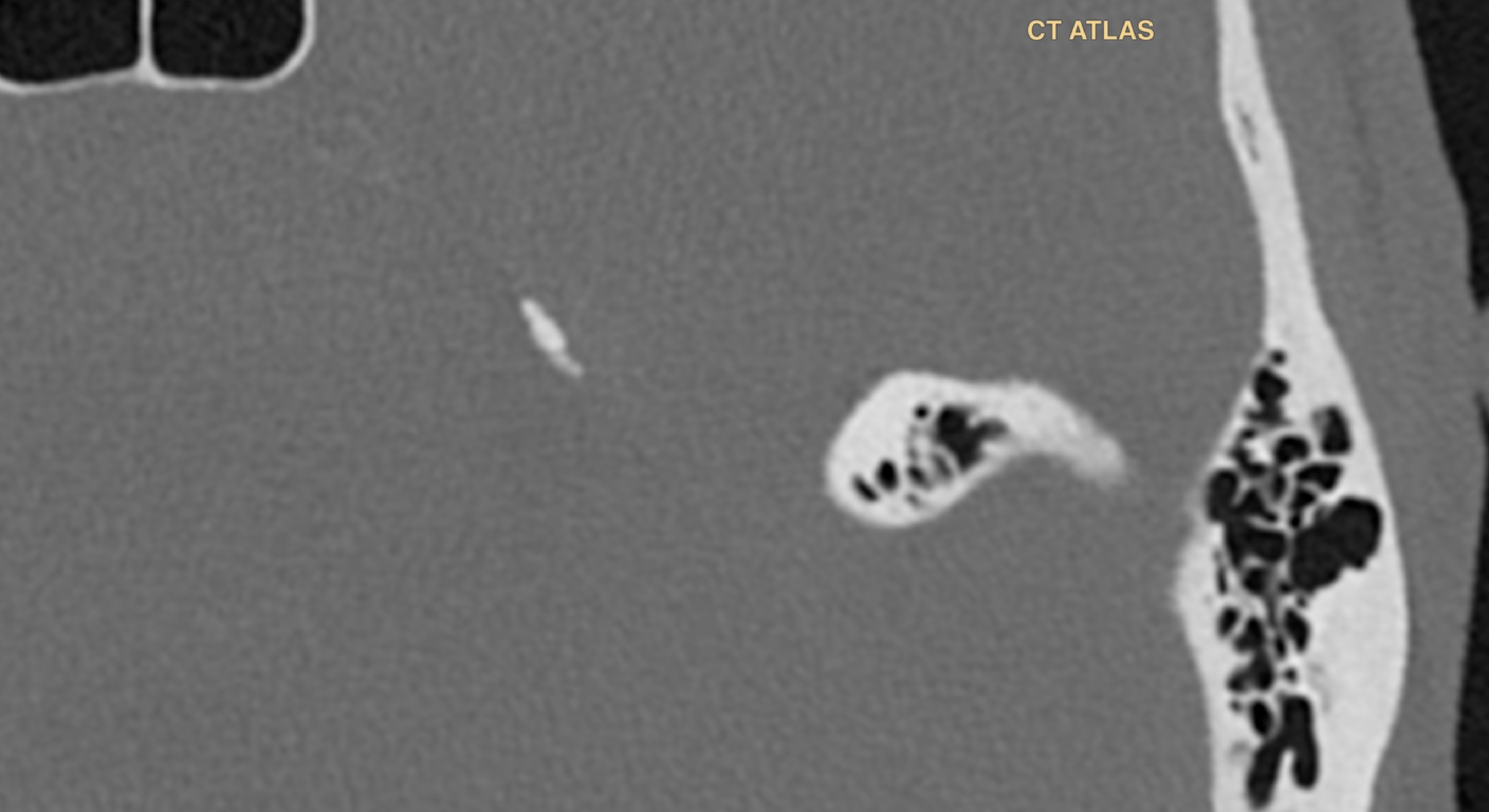
CT ATLAS

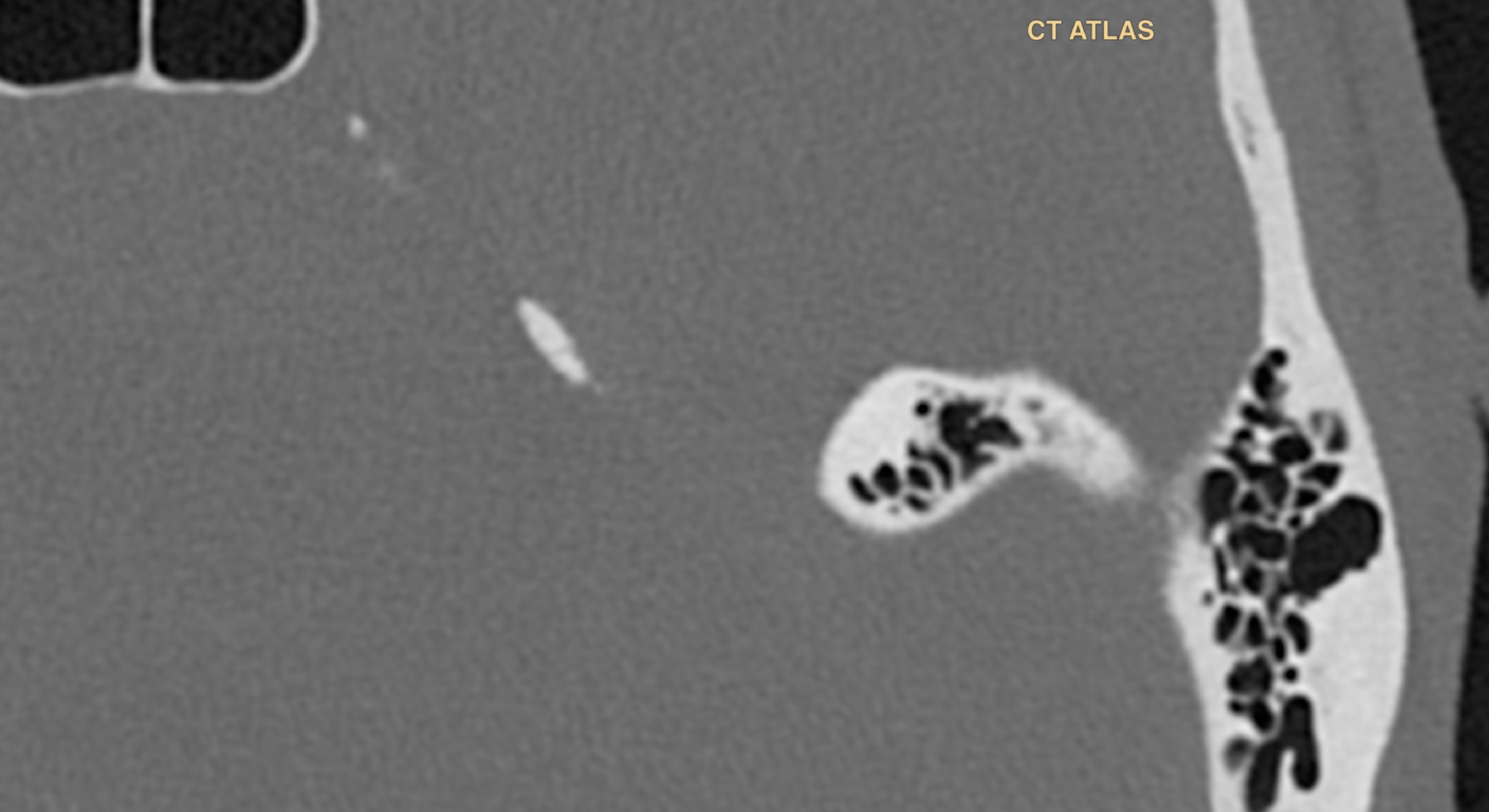
CT ATLAS

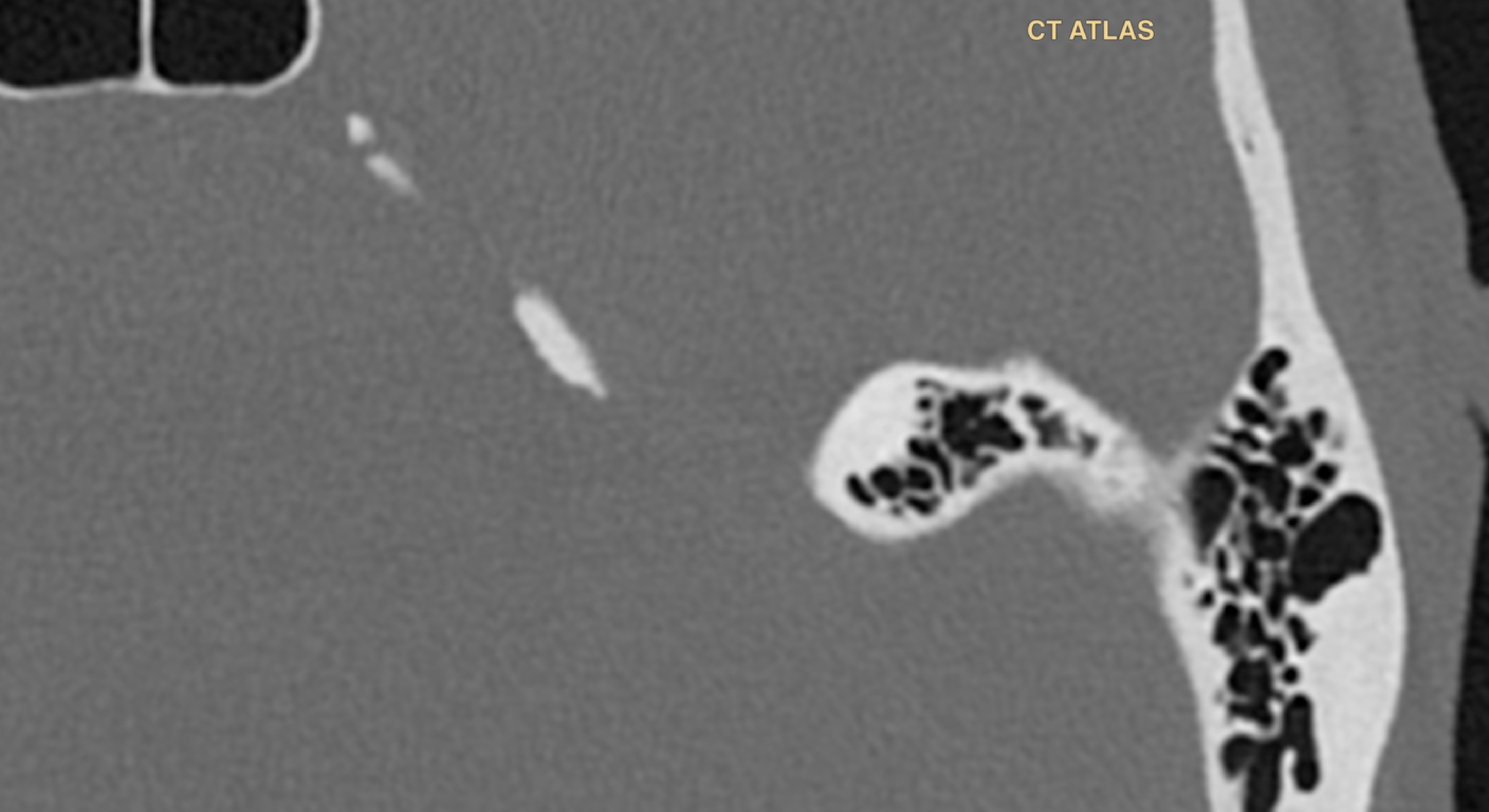
CT ATLAS

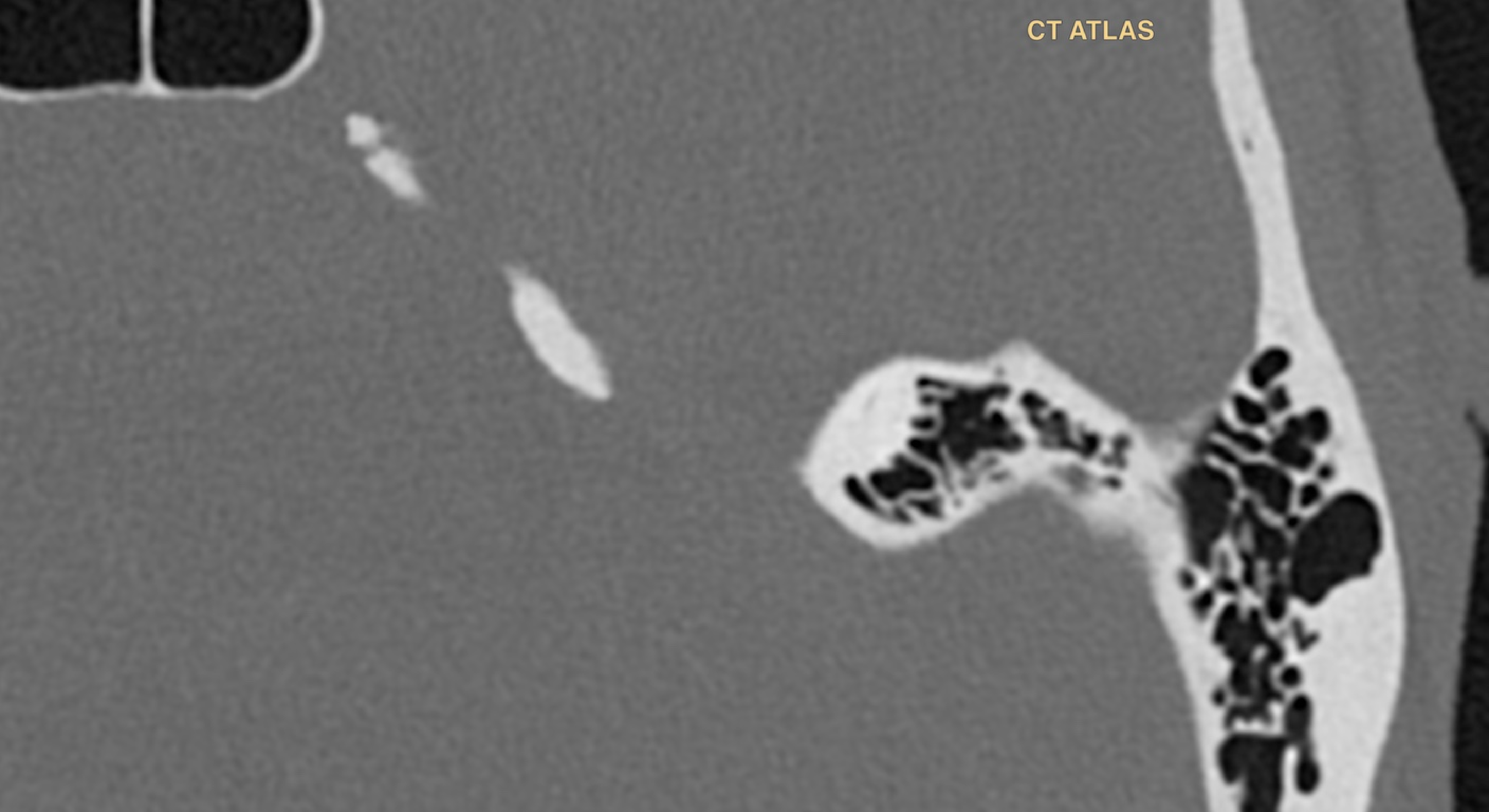
CT ATLAS

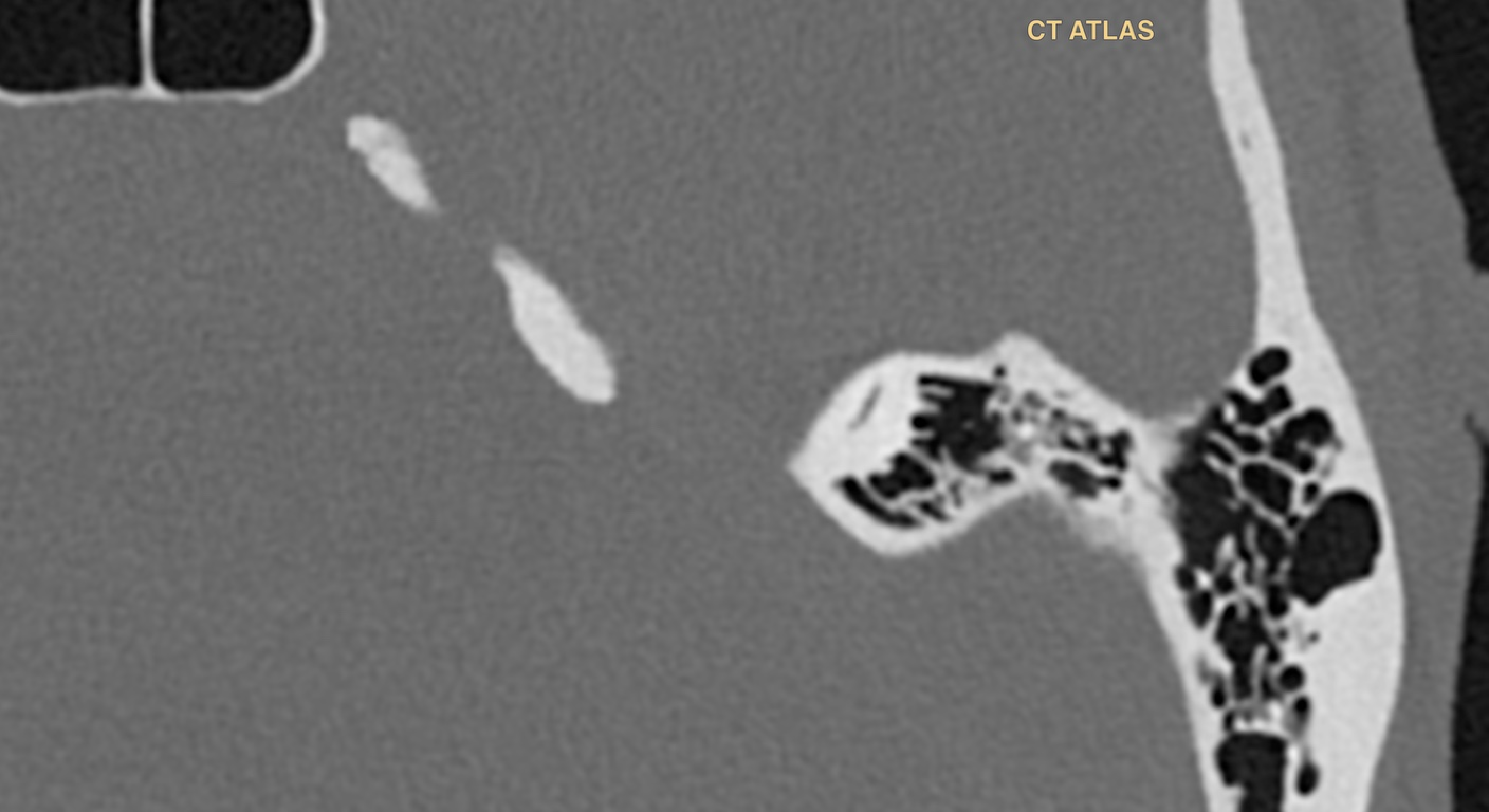
CT ATLAS

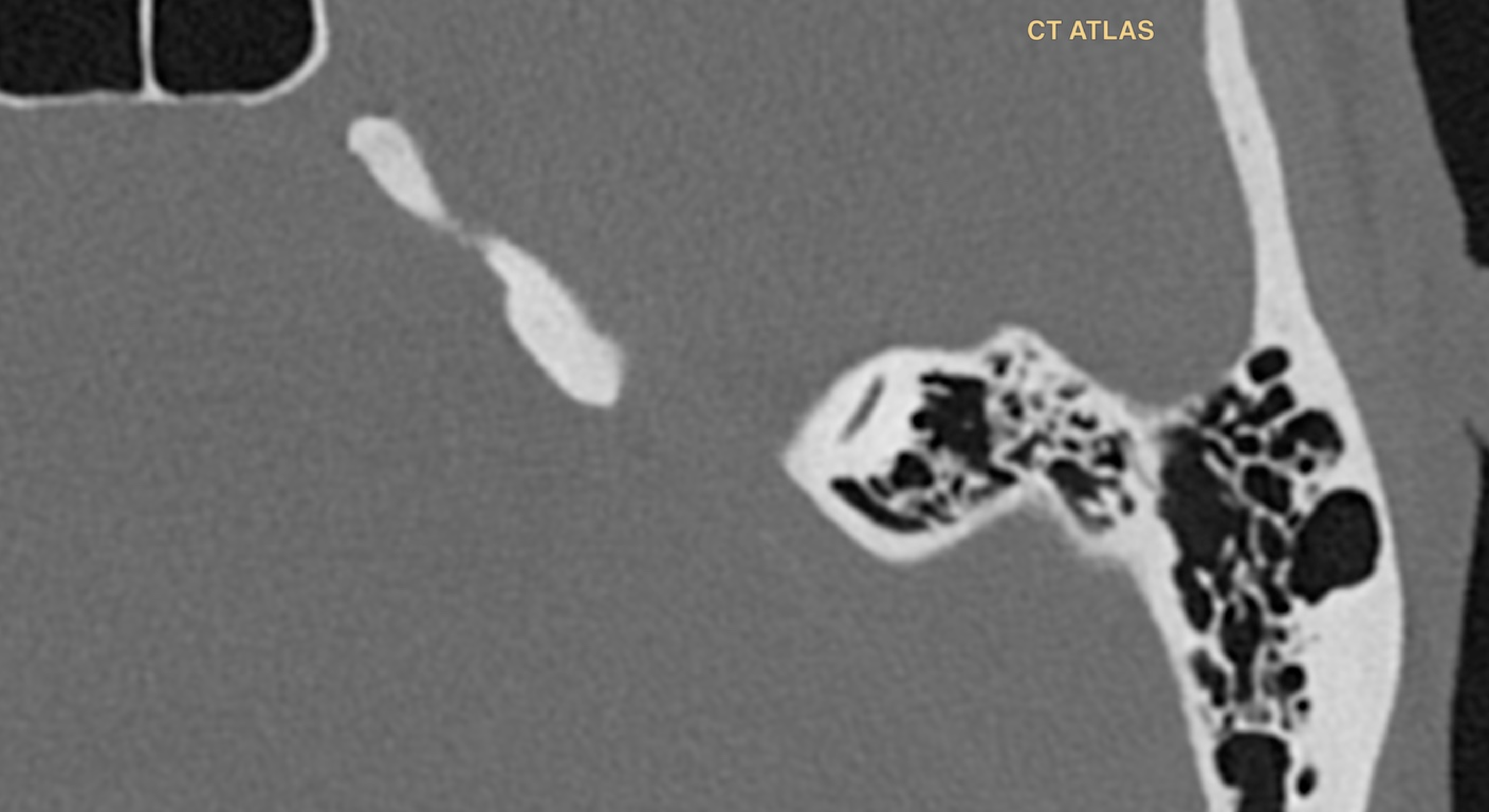
CT ATLAS

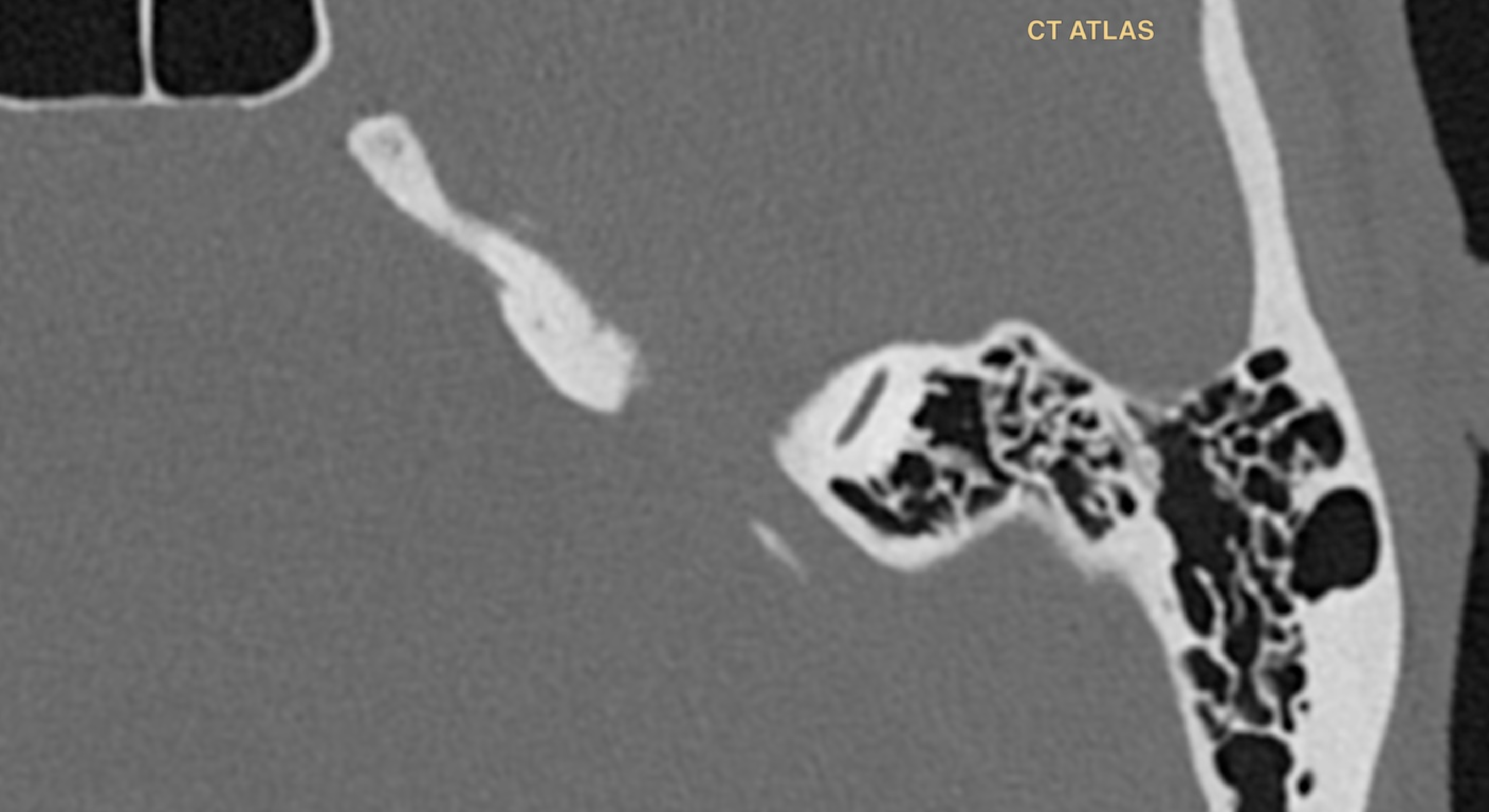
CT ATLAS

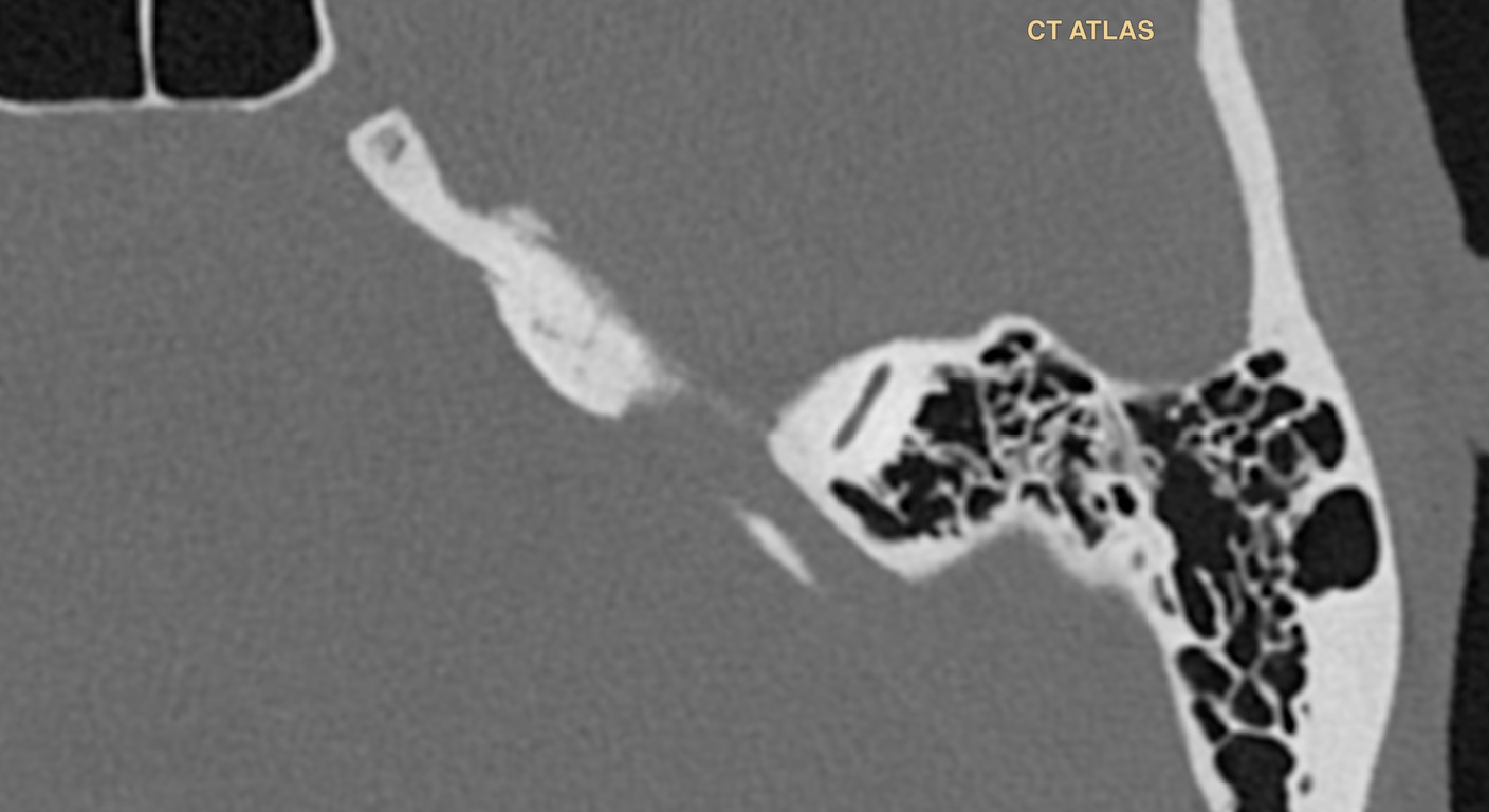
CT ATLAS

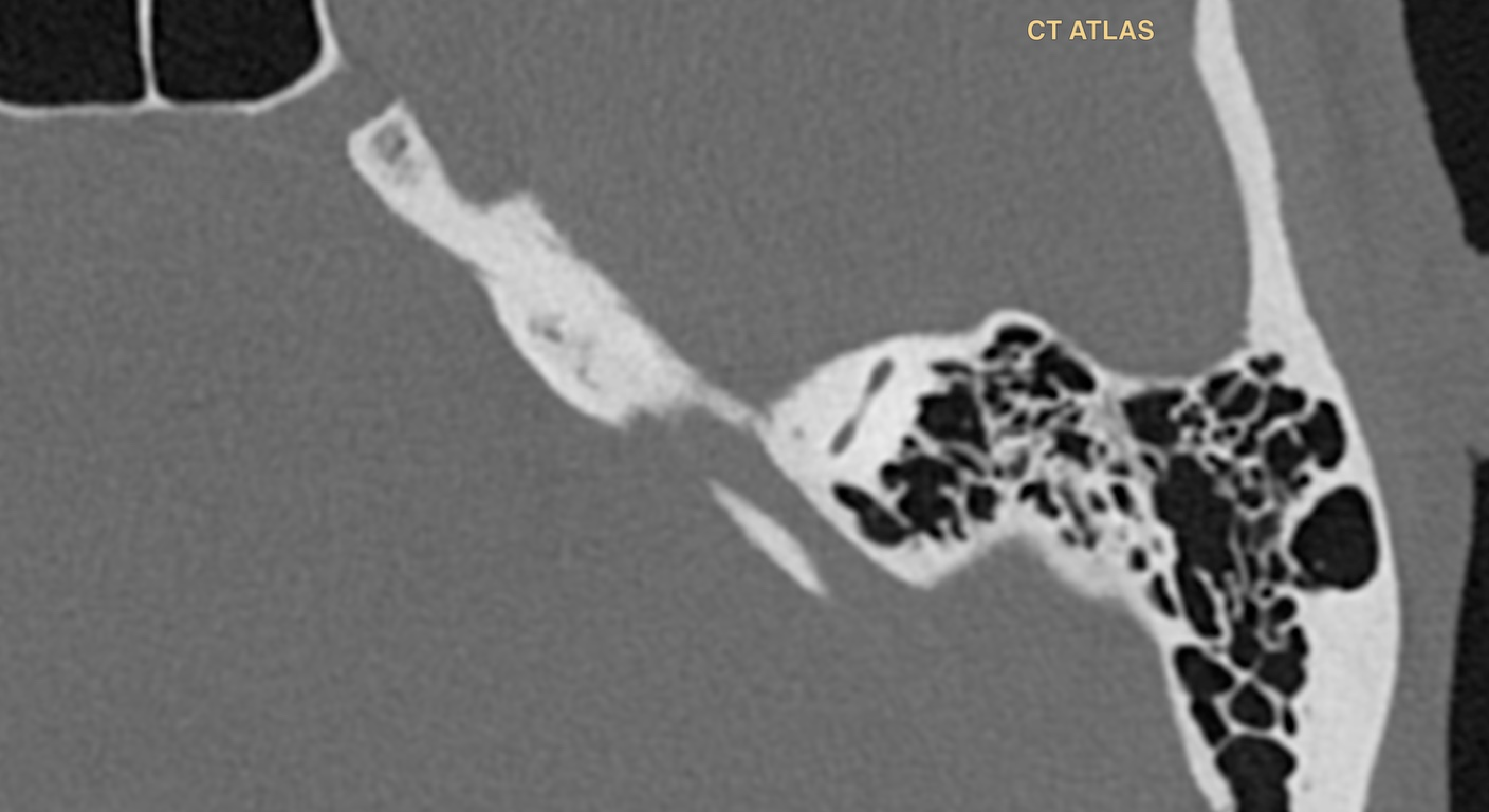
CT ATLAS

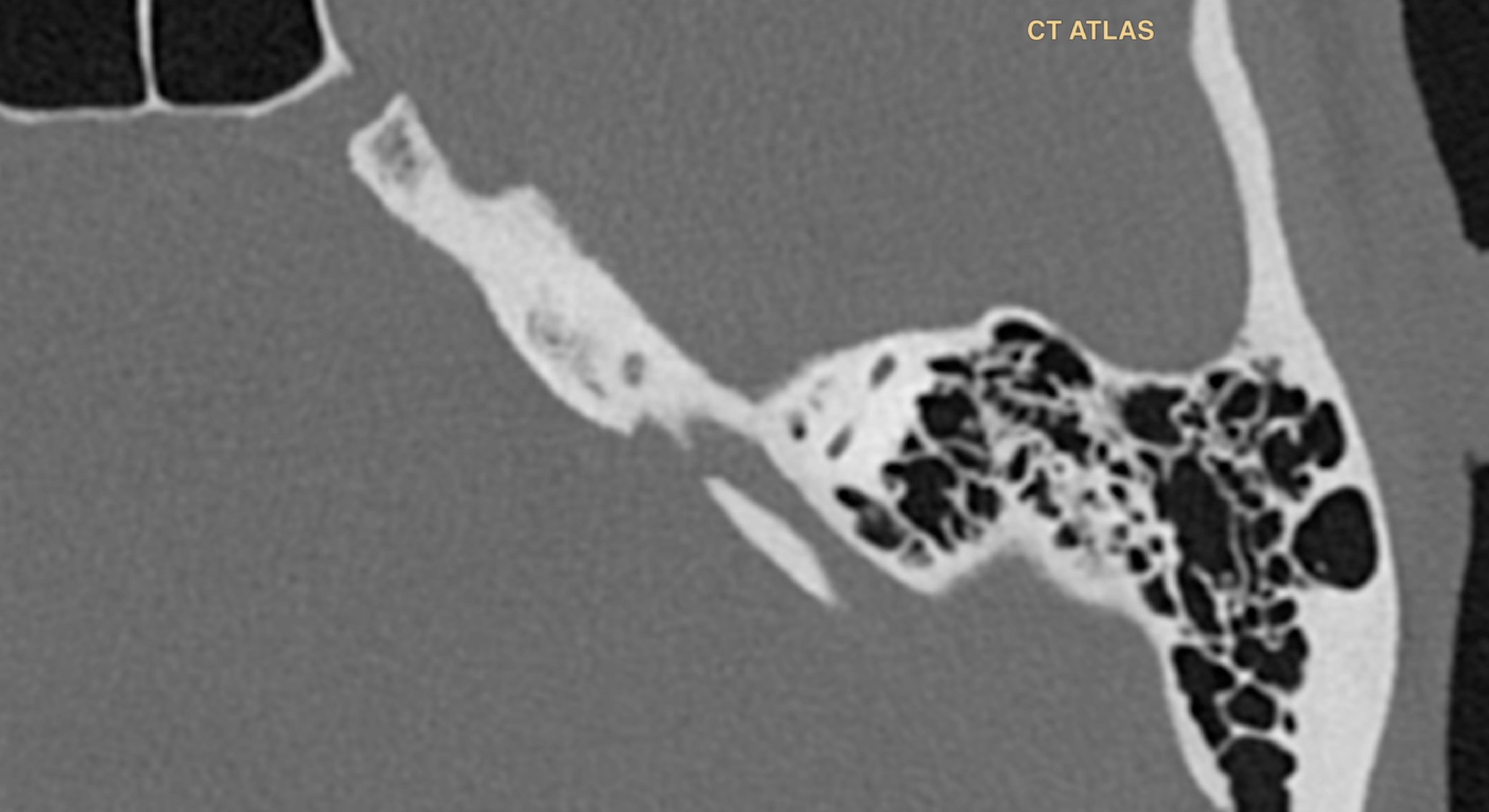
CT ATLAS

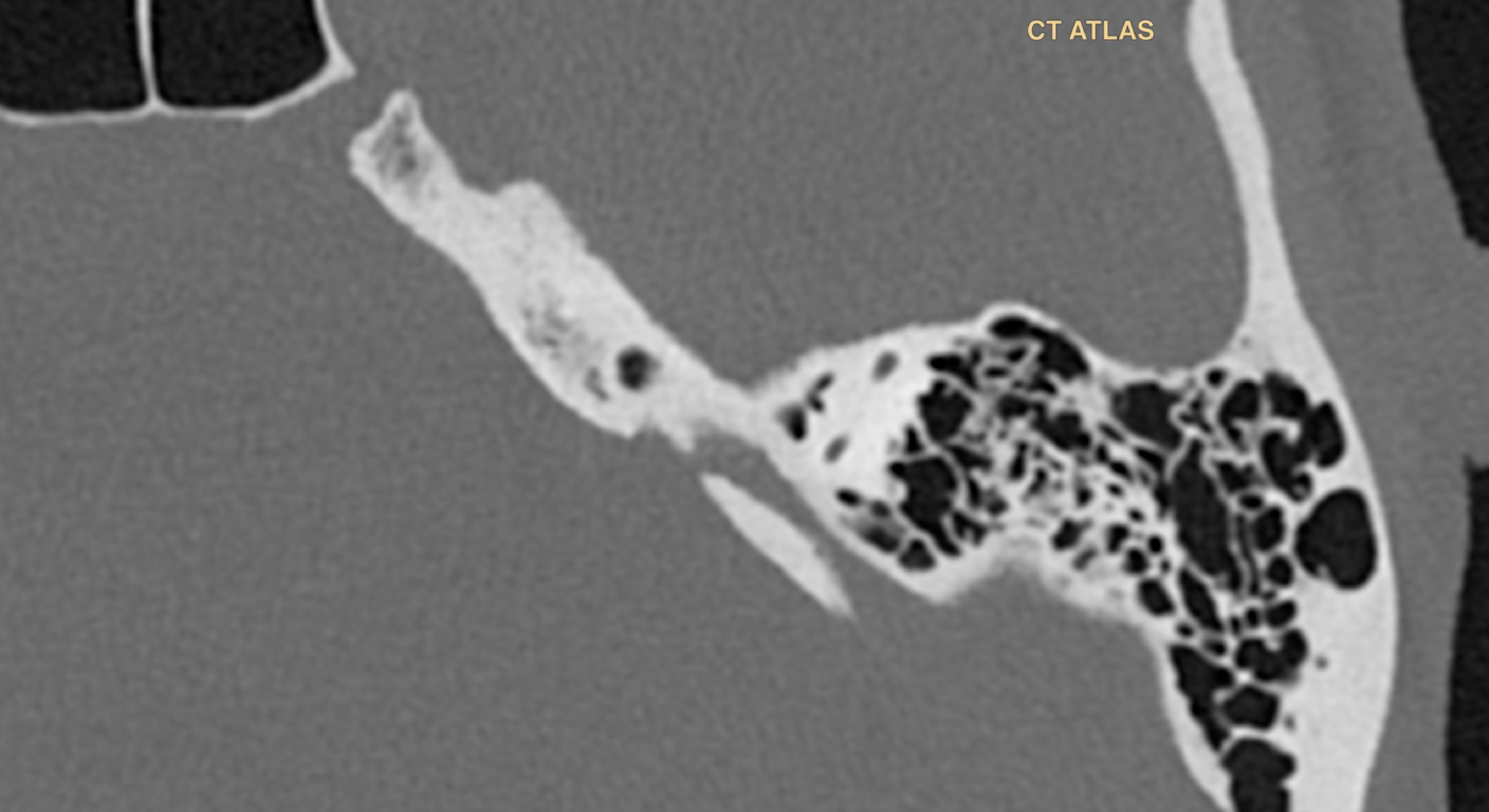
CT ATLAS

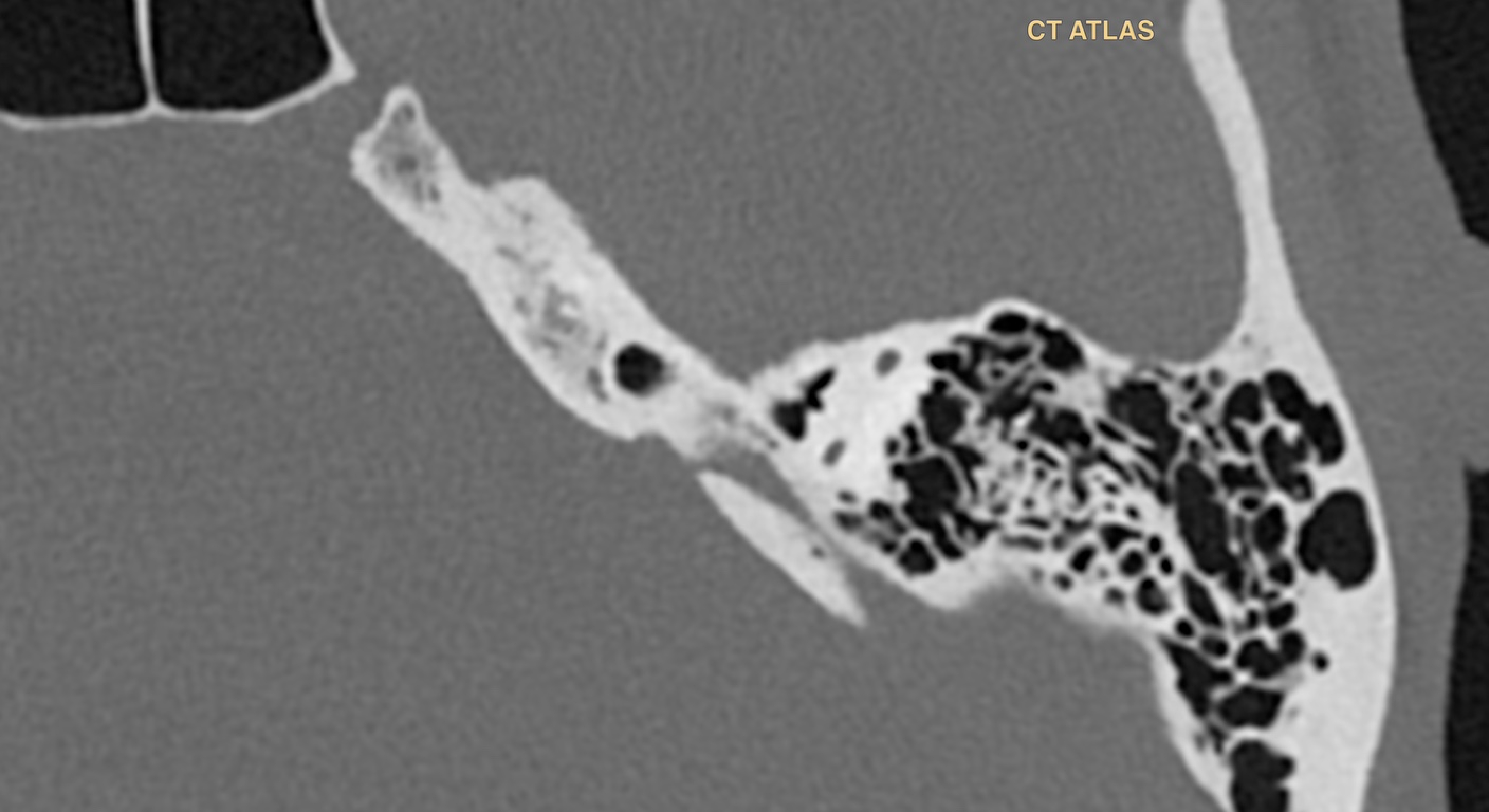
CT ATLAS

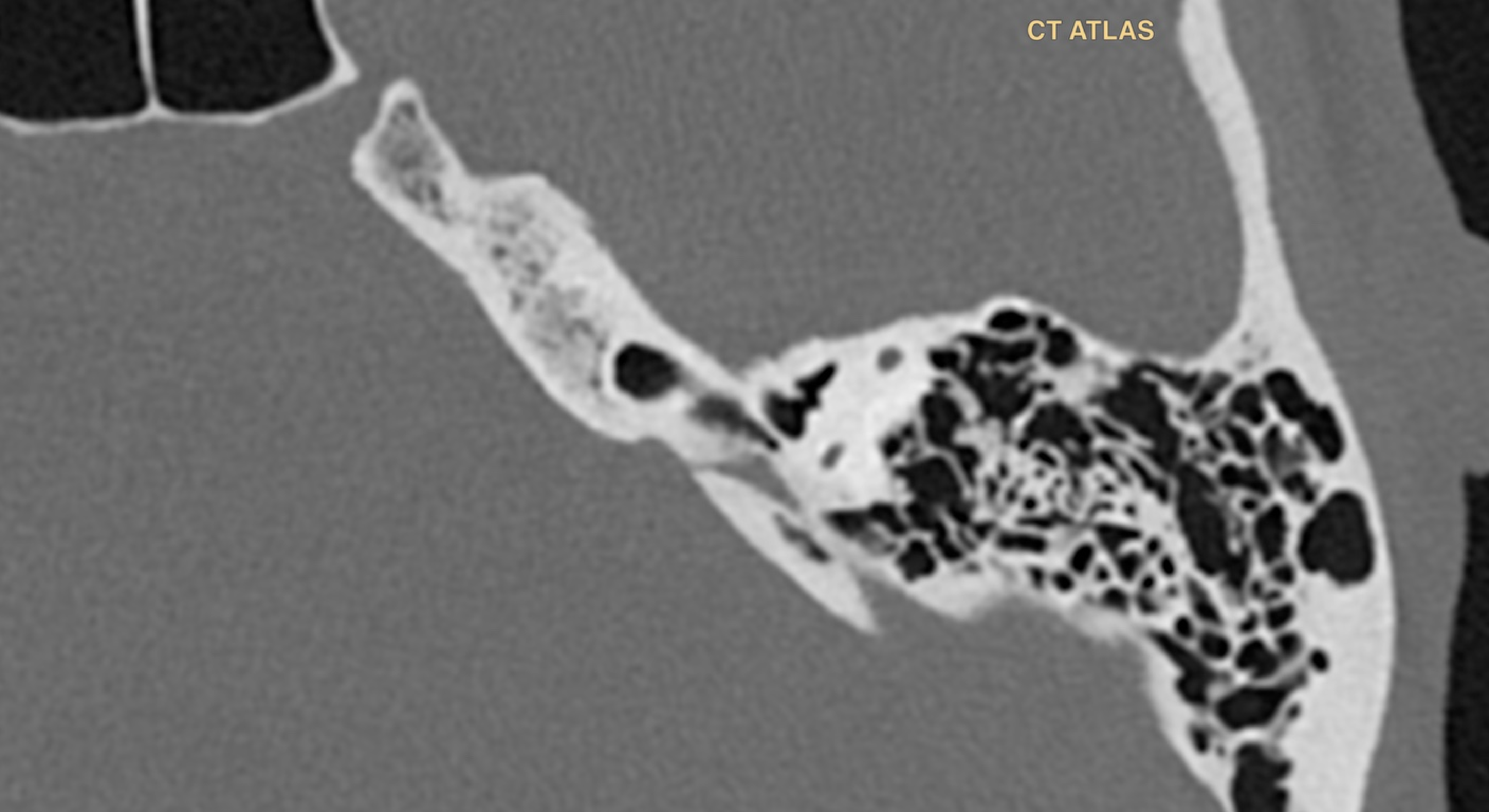
CT ATLAS

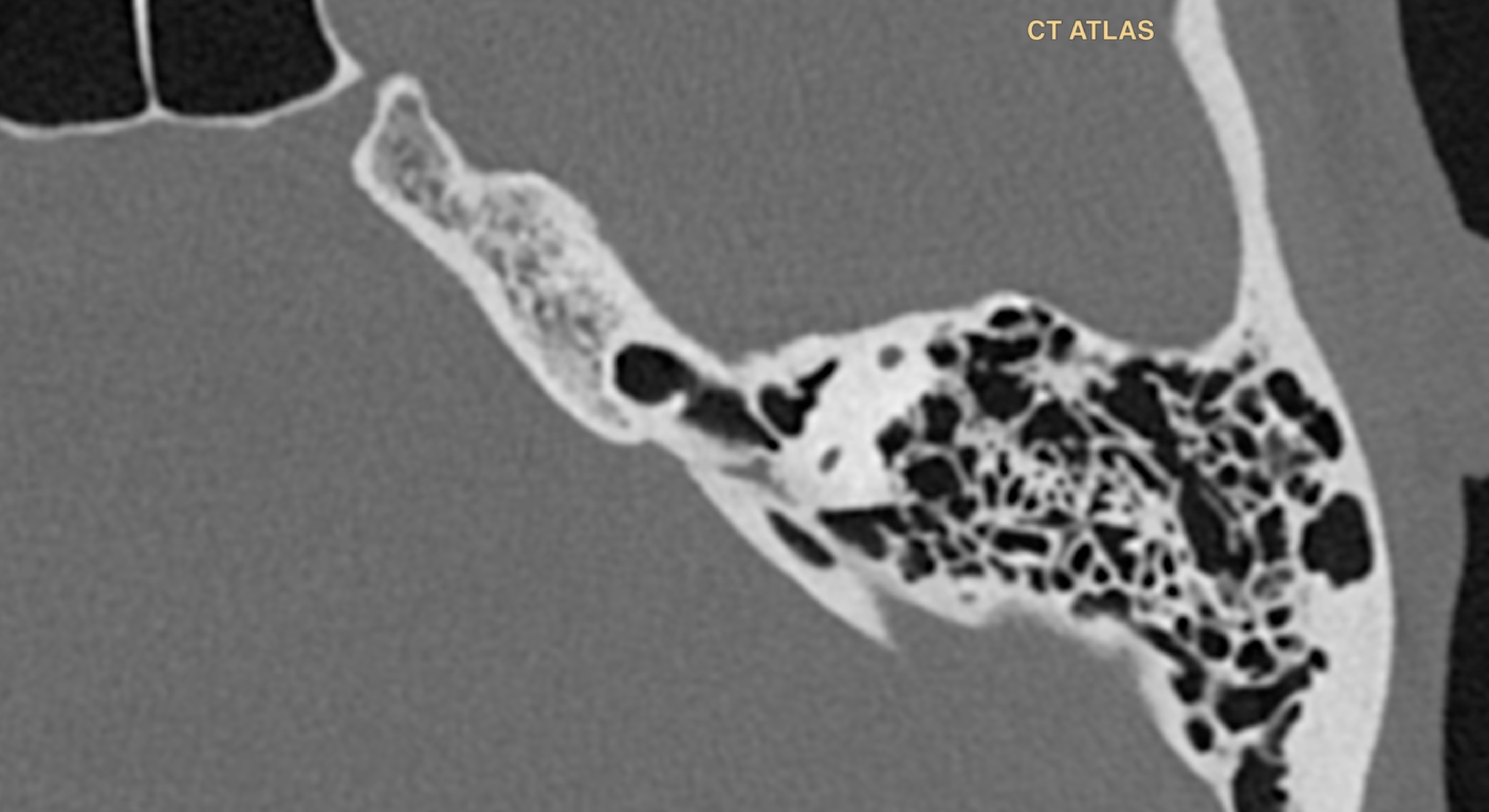
CT ATLAS

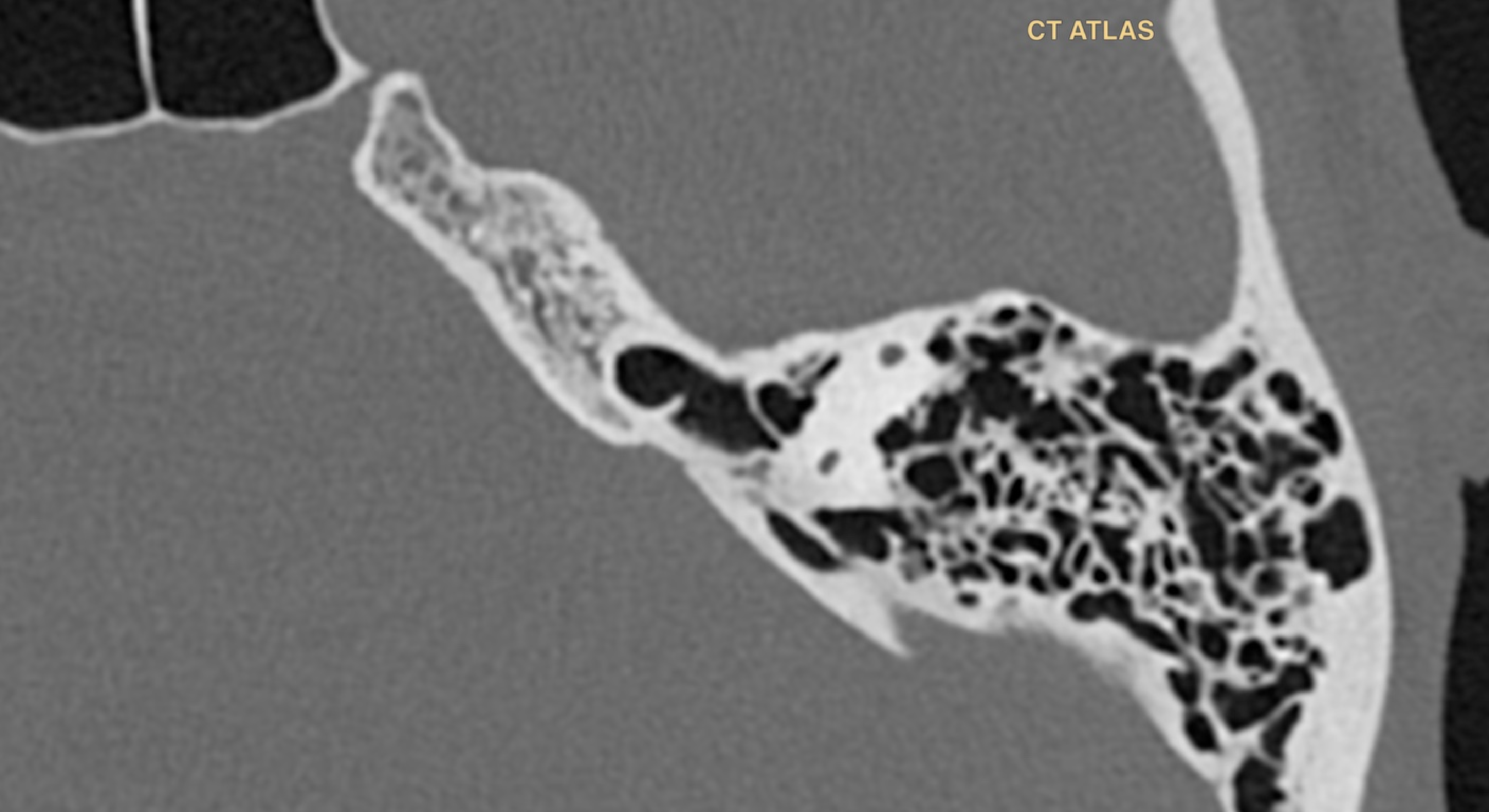
CT ATLAS

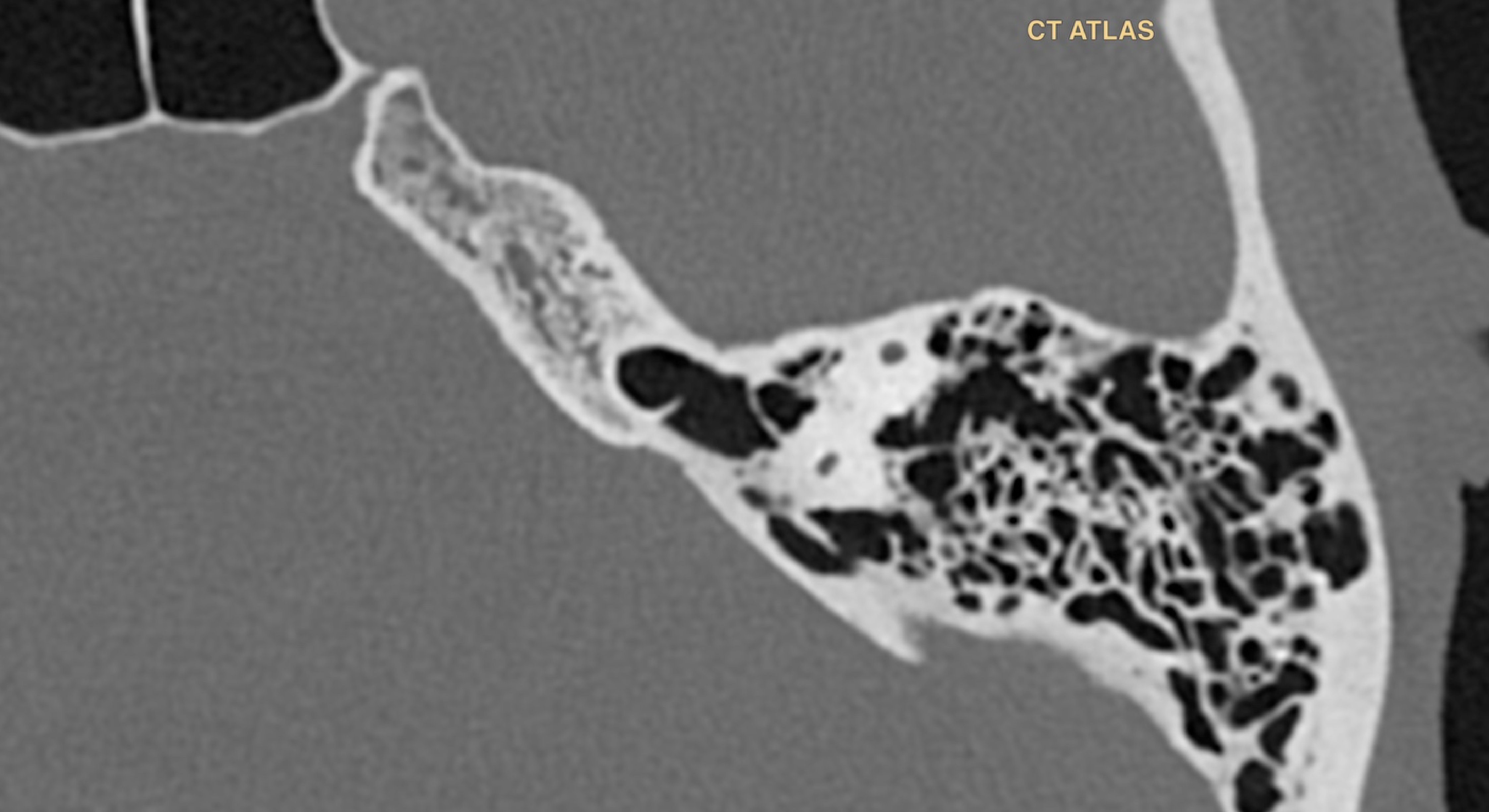

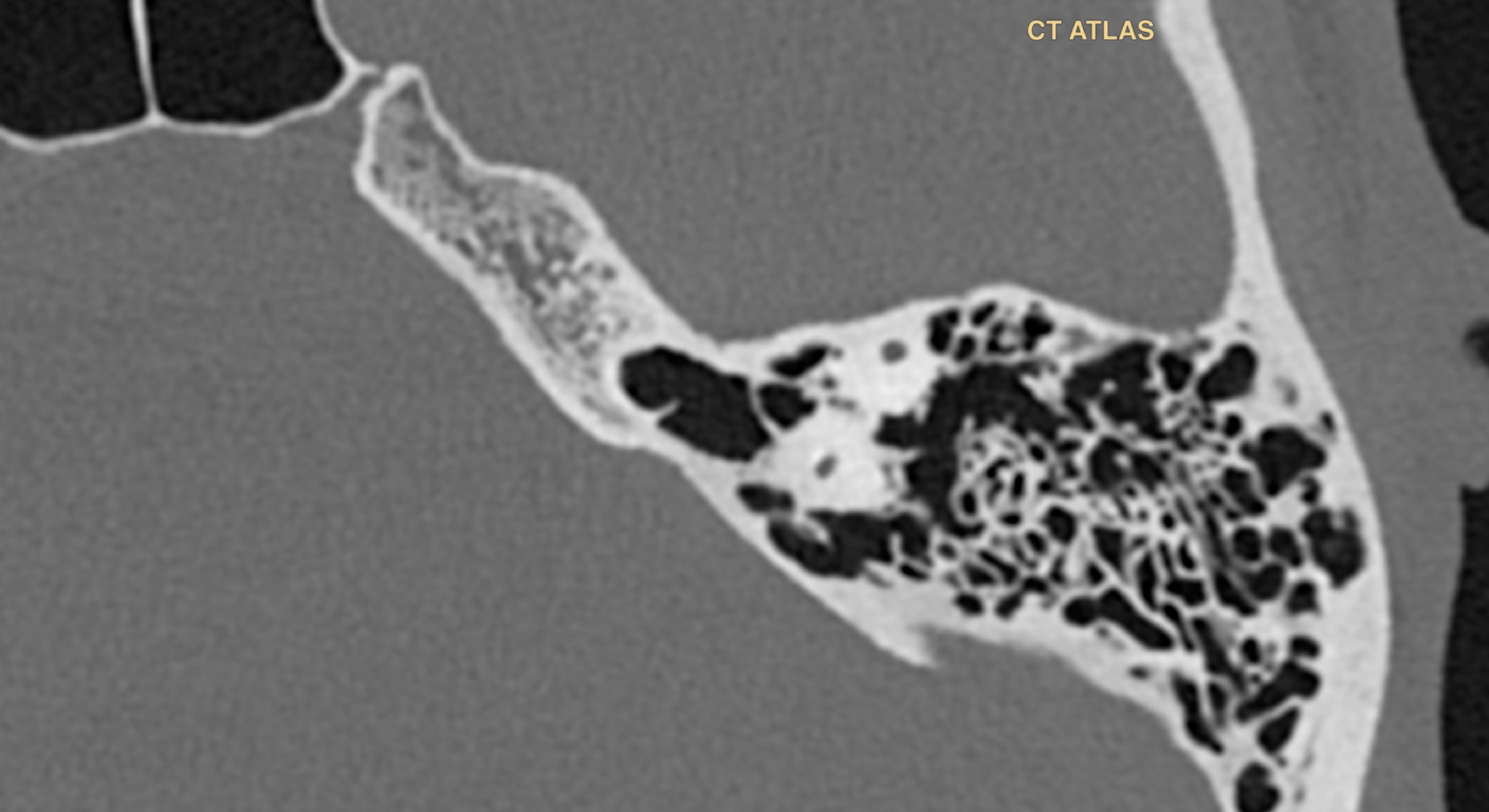
CT ATLAS

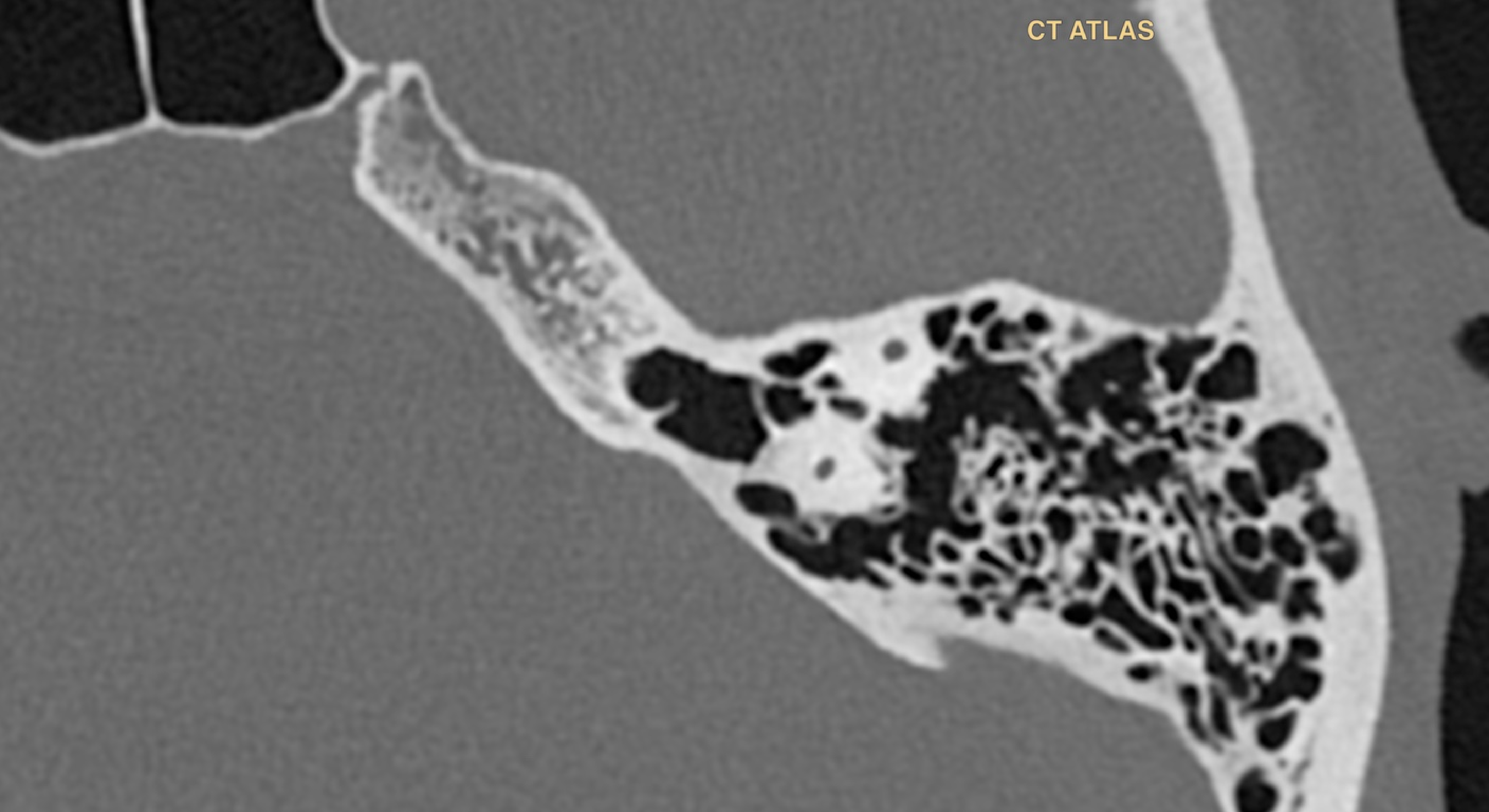

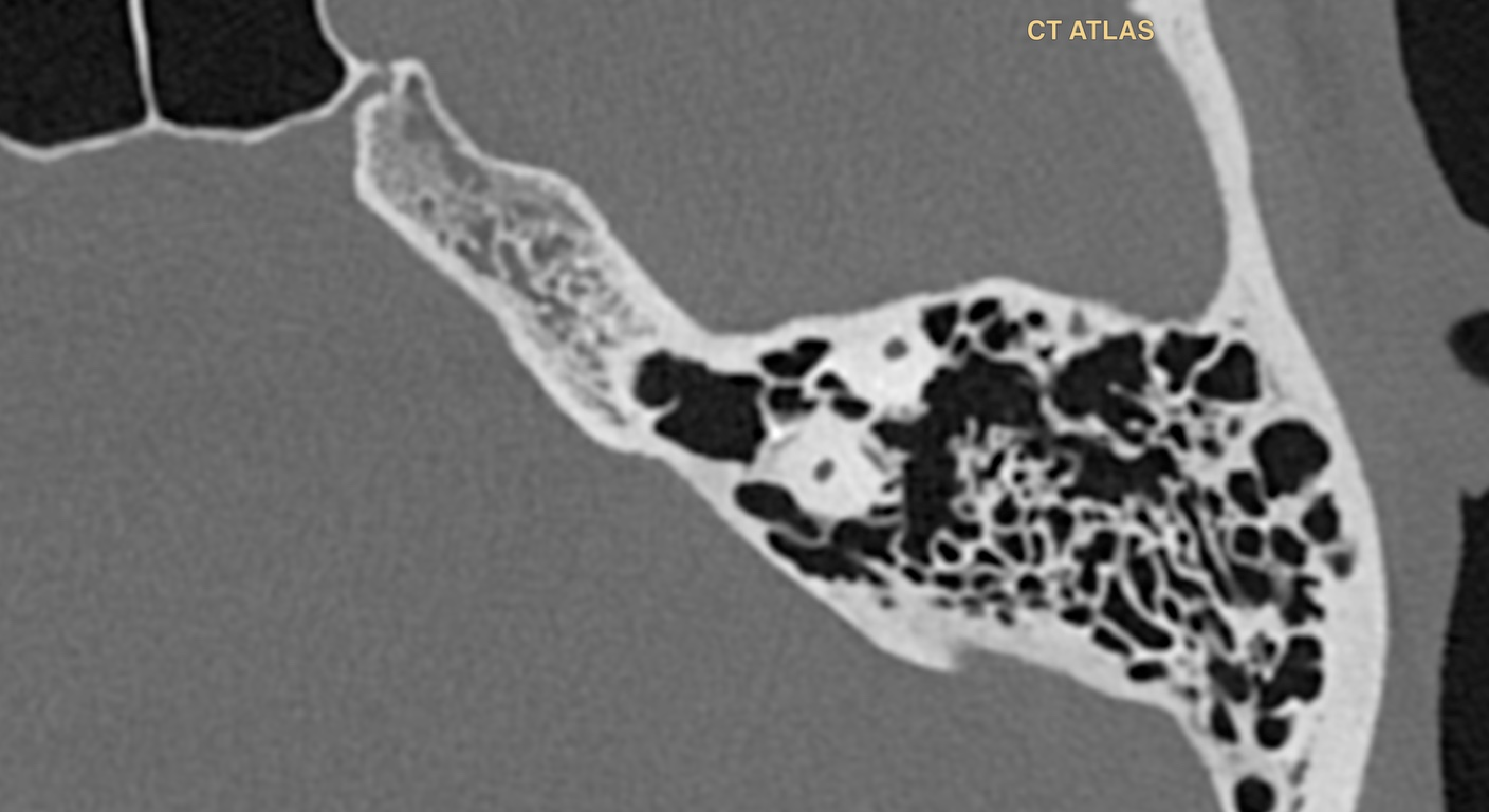
CT ATLAS

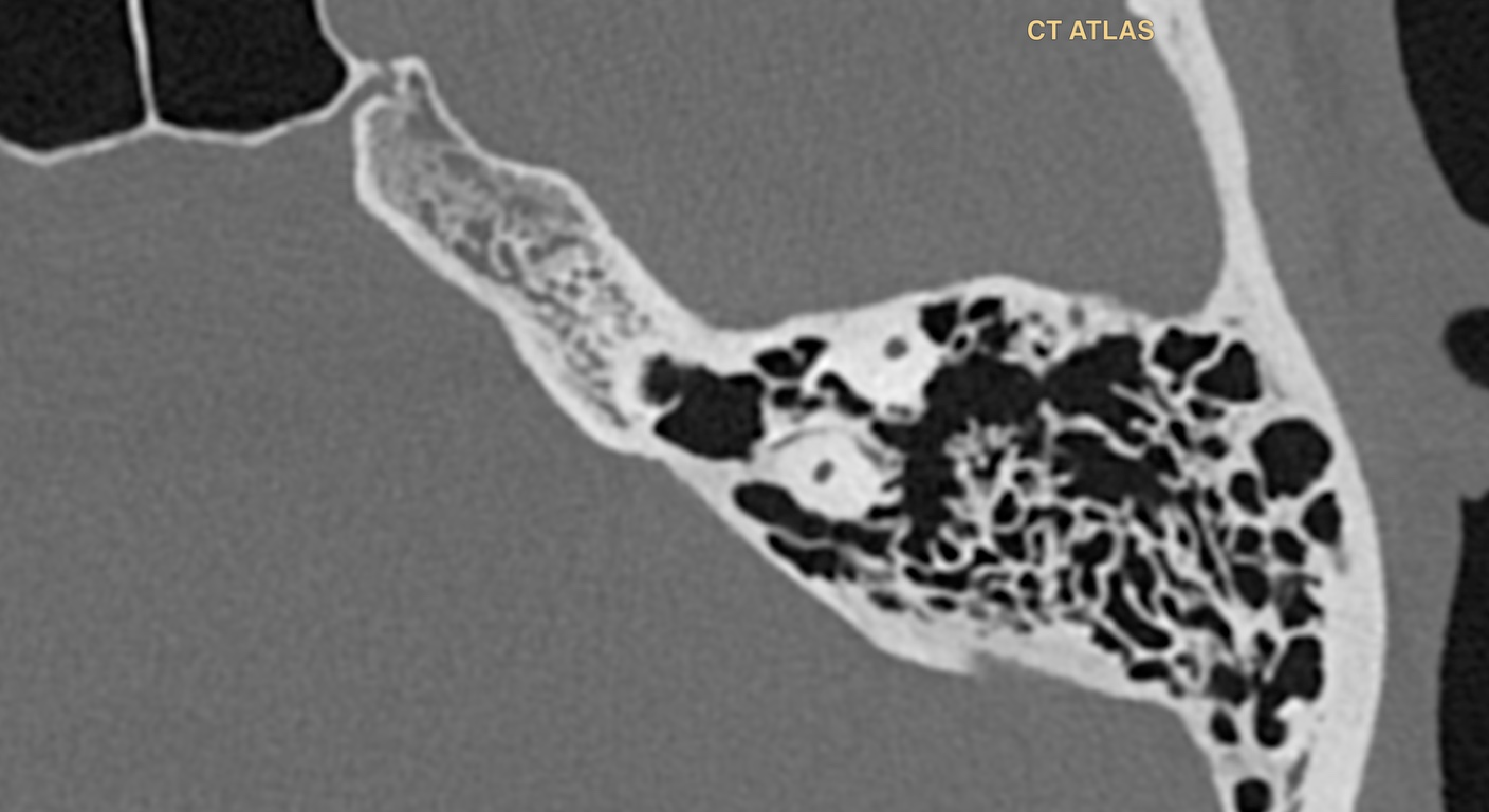
CT ATLAS

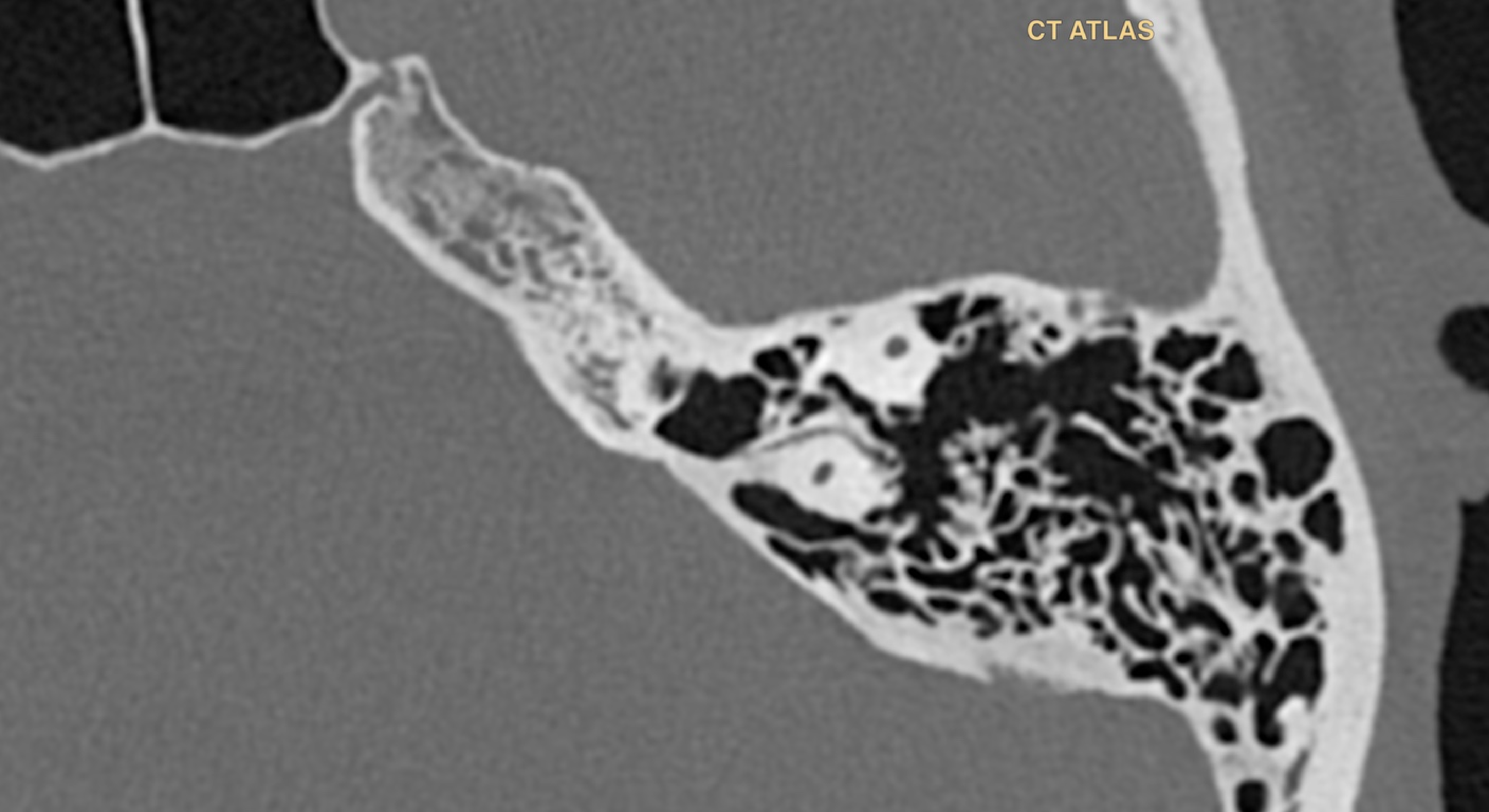
CT ATLAS

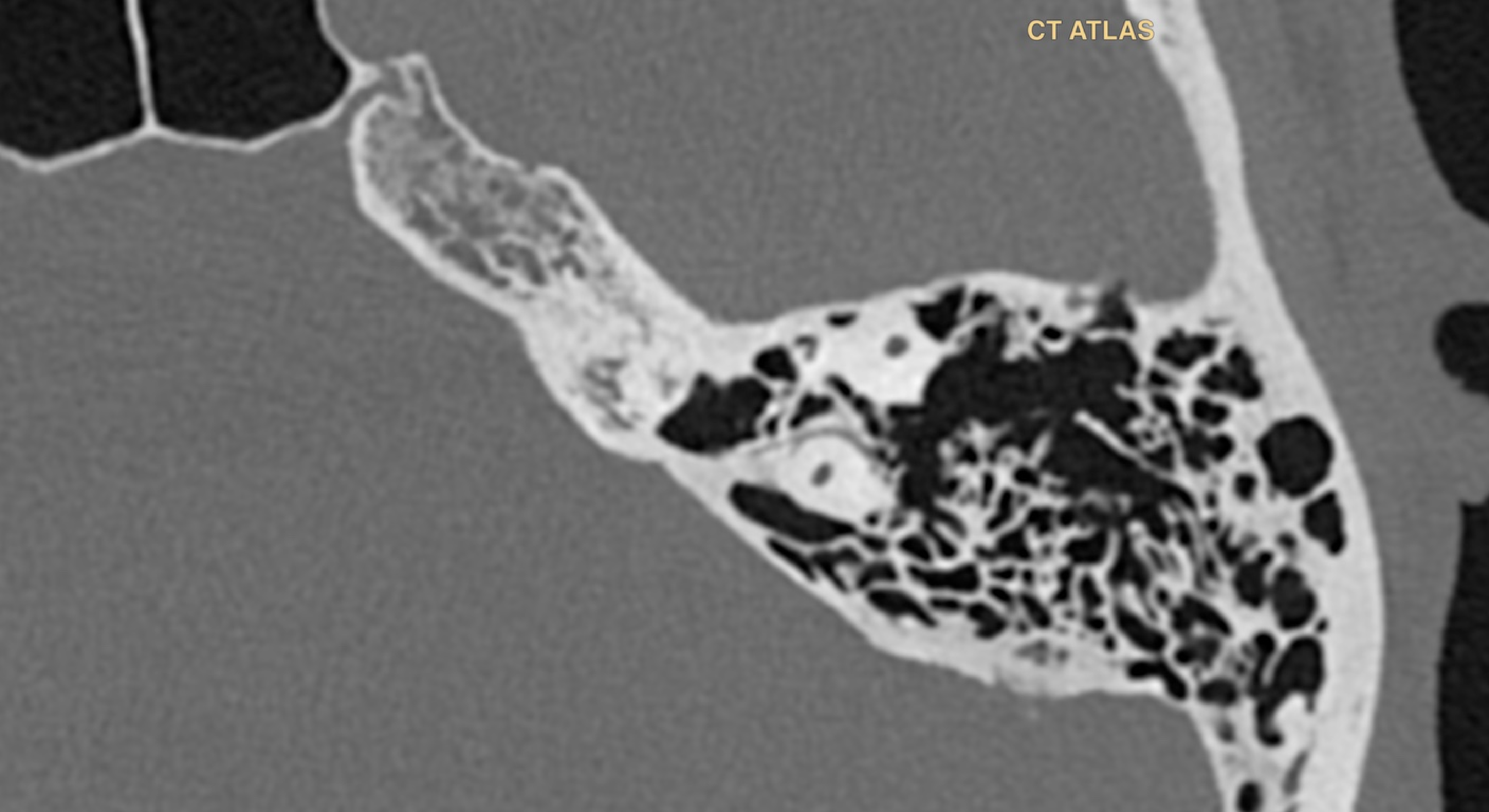
CT ATLAS

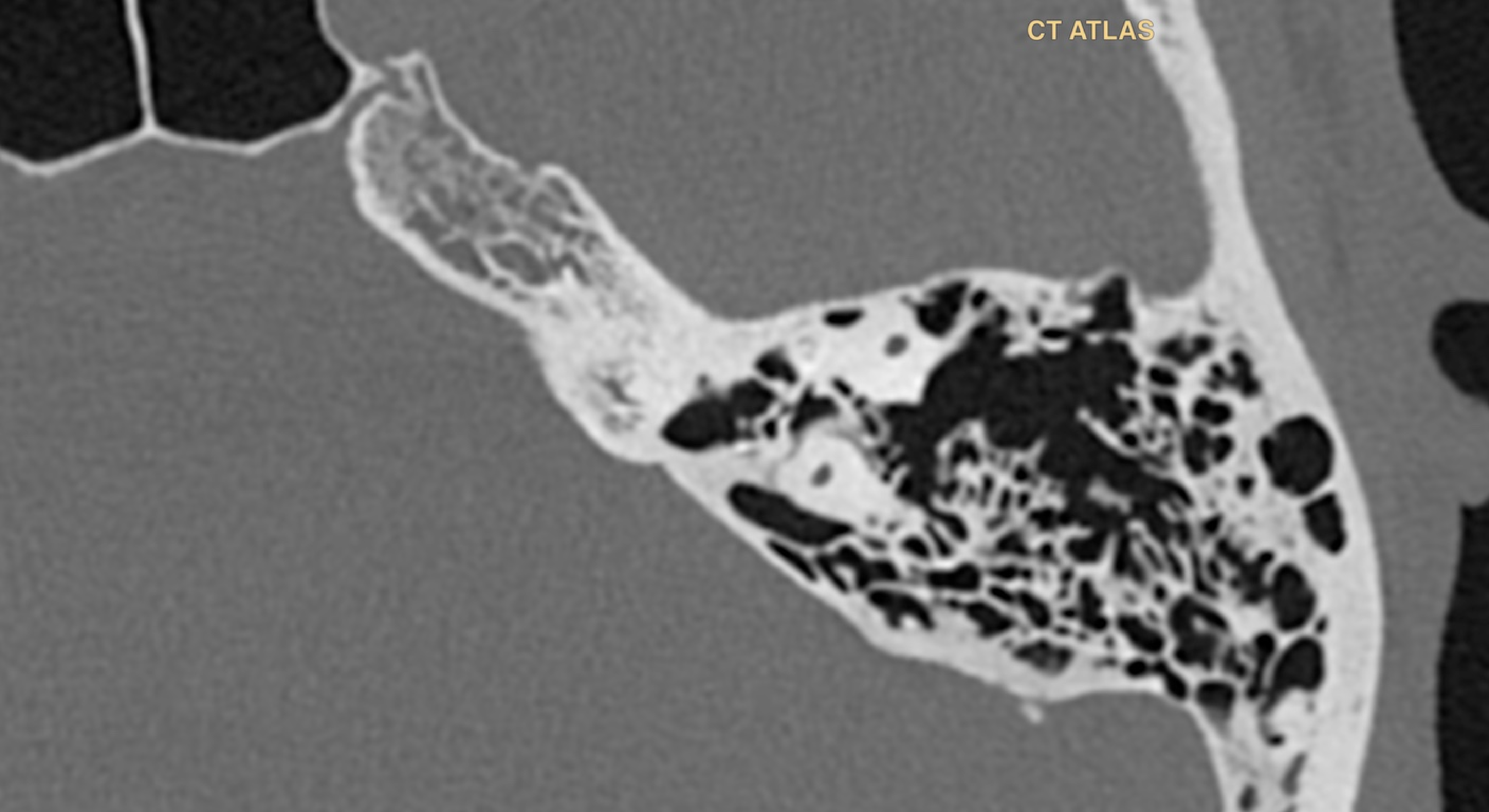
CT ATLAS

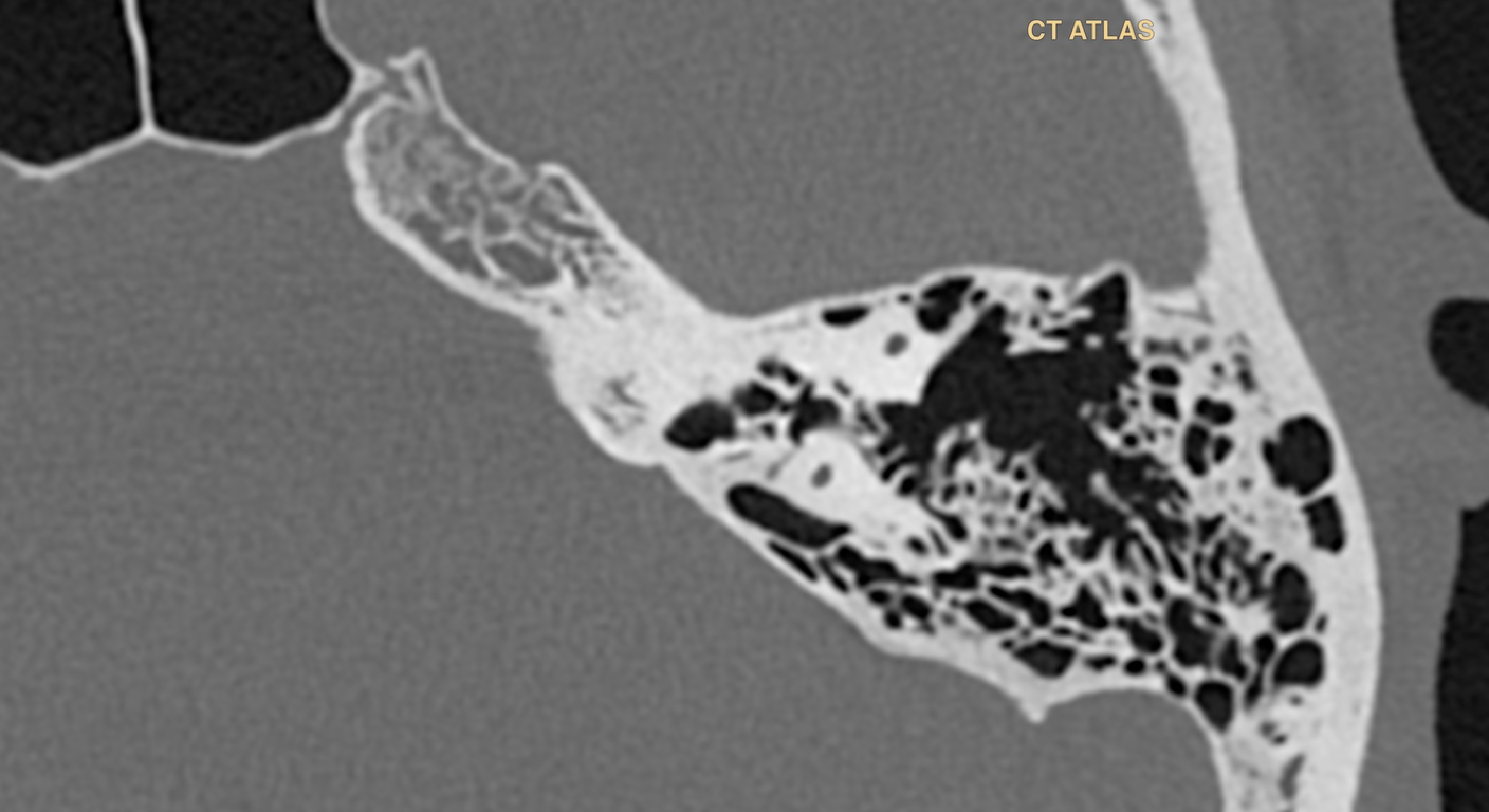
CT ATLAS

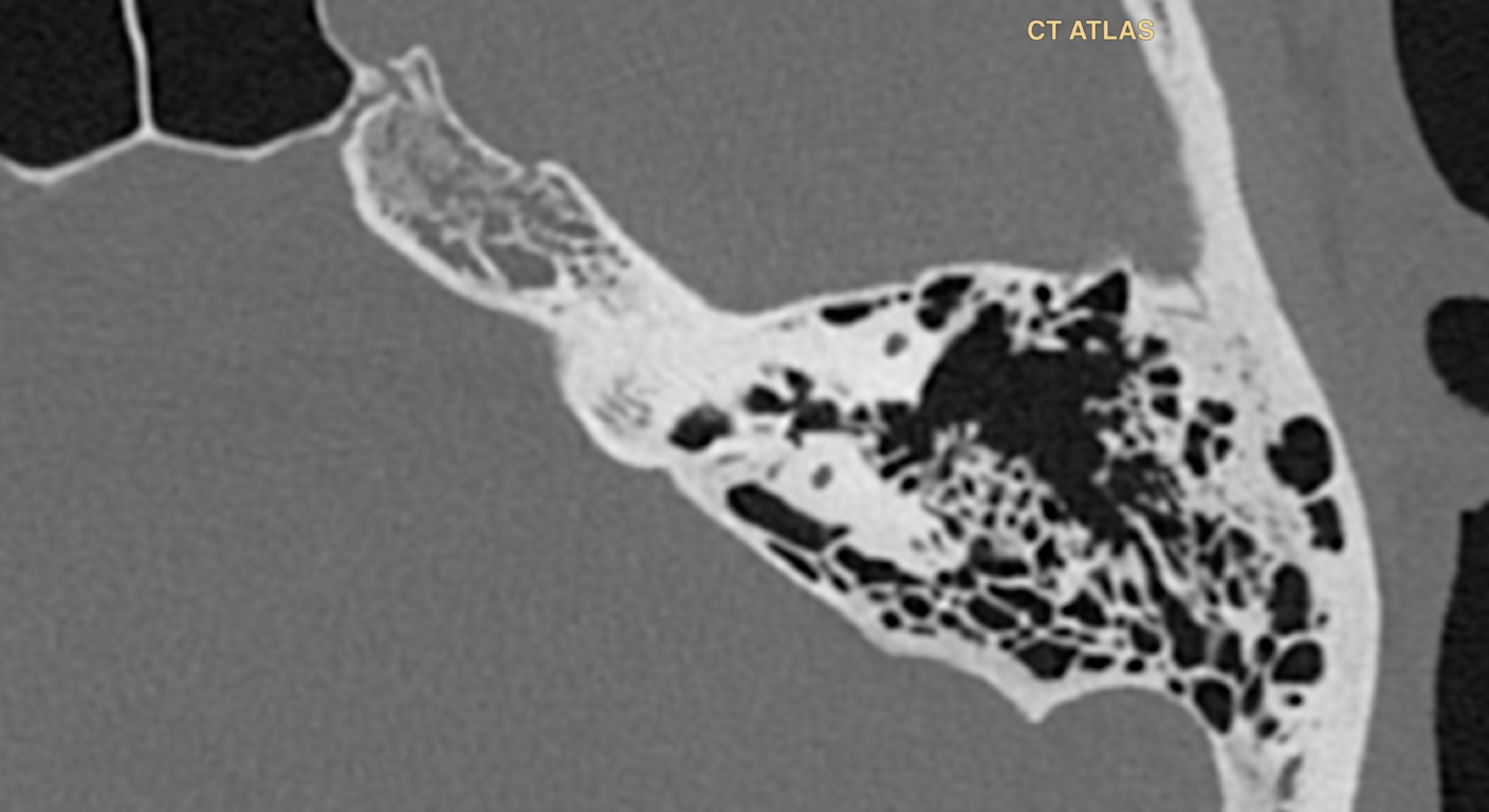
CT ATLAS

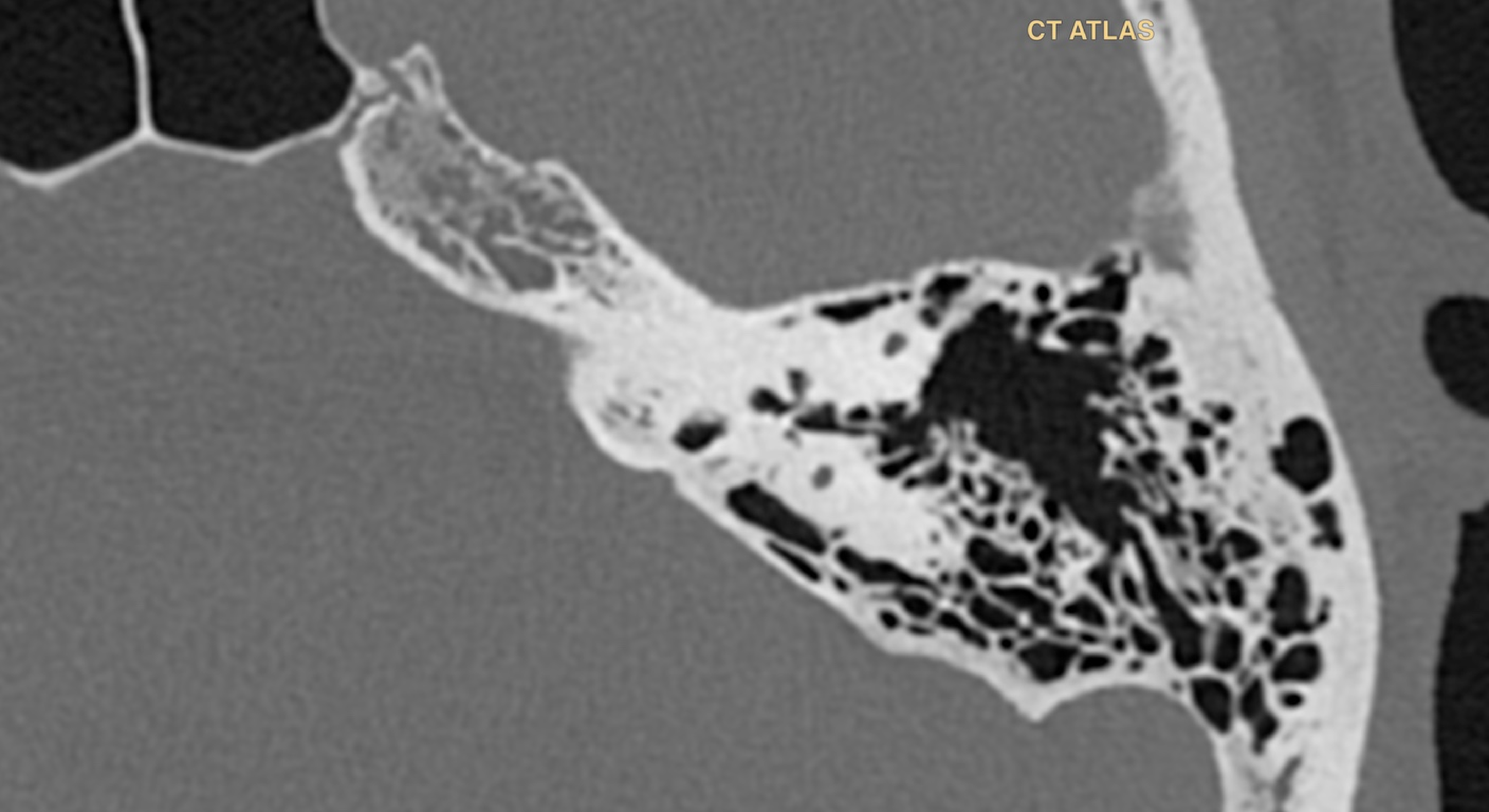
CT ATLAS

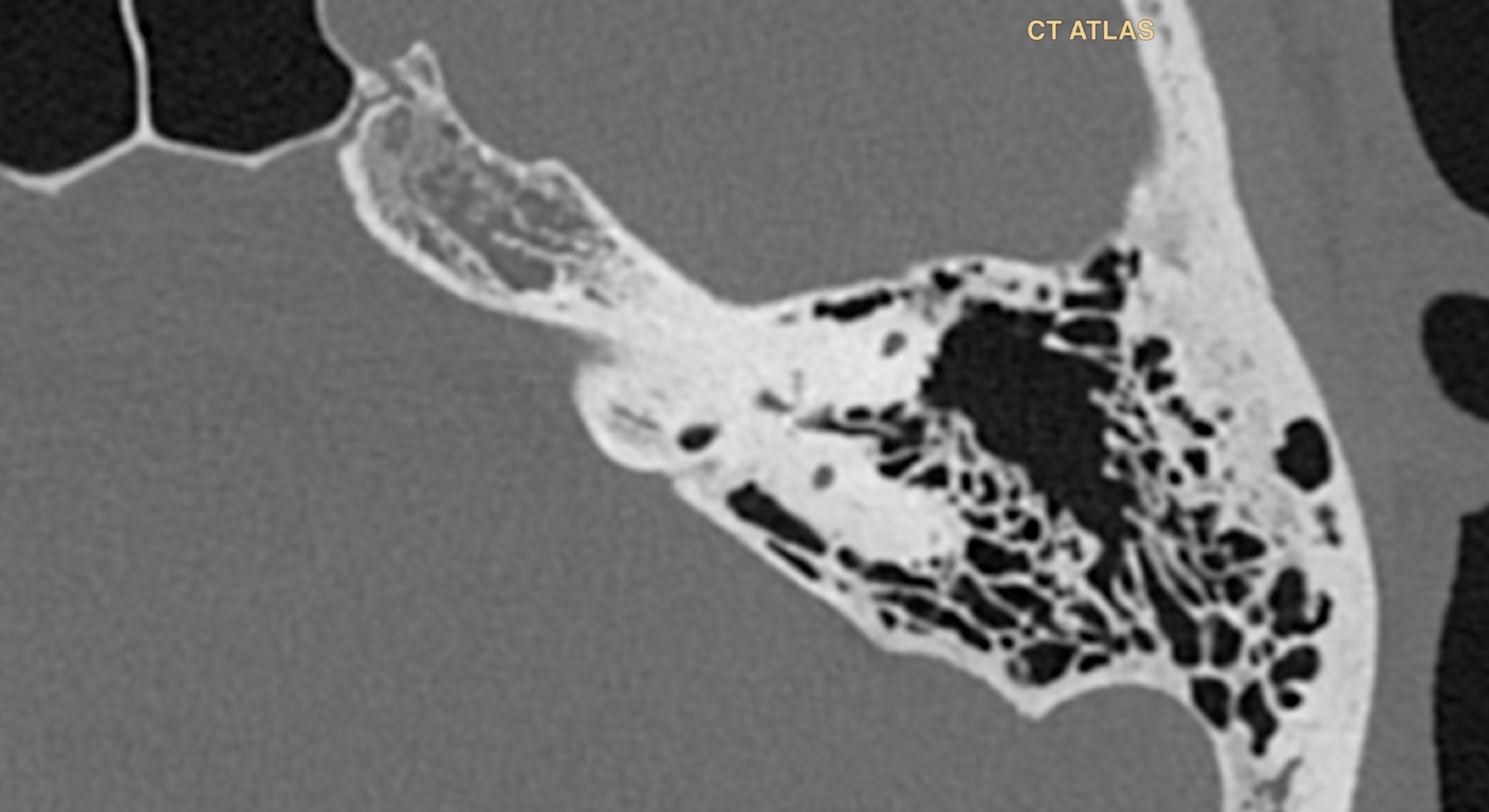
CT ATLAS

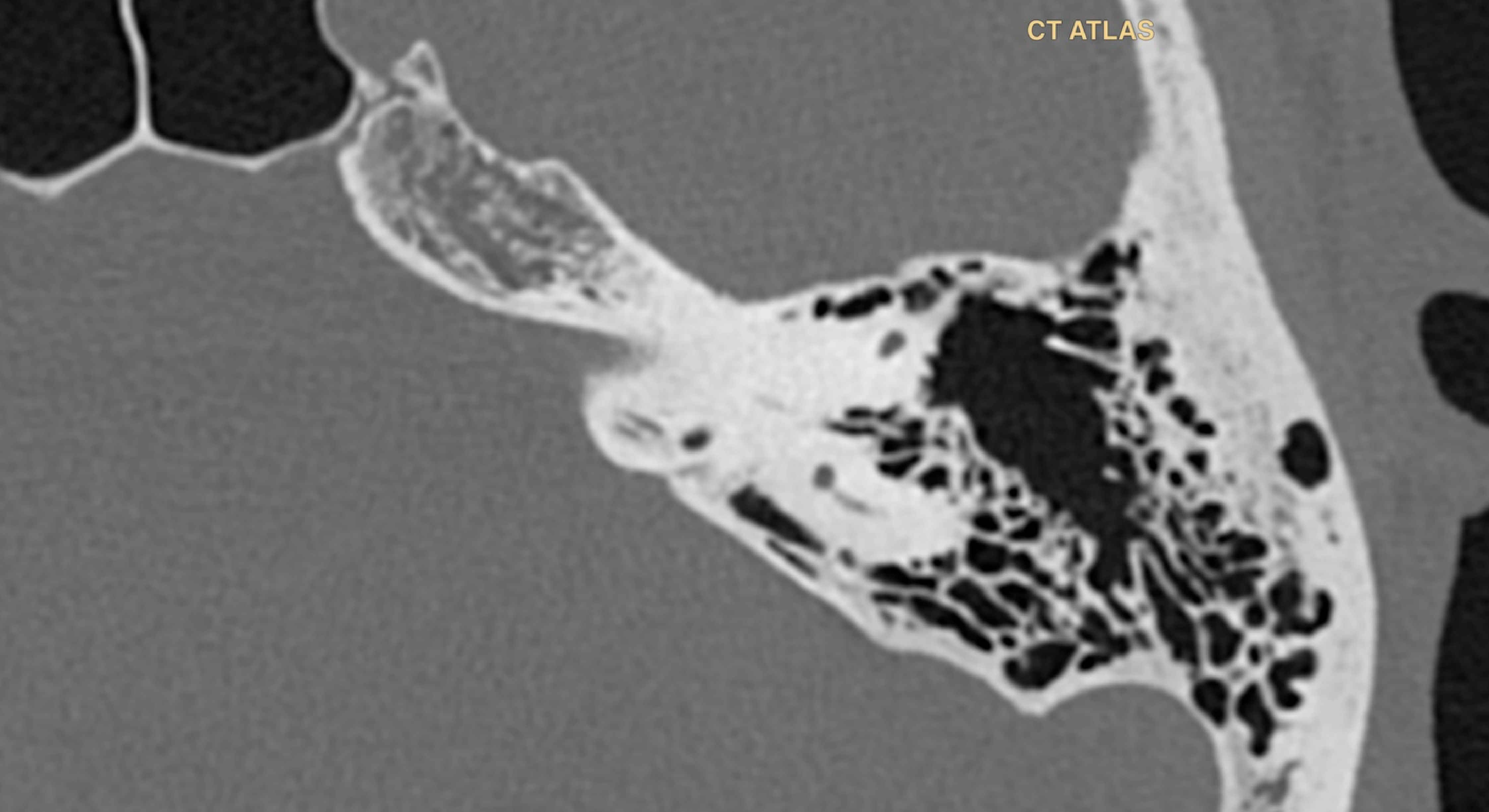
CT ATLAS

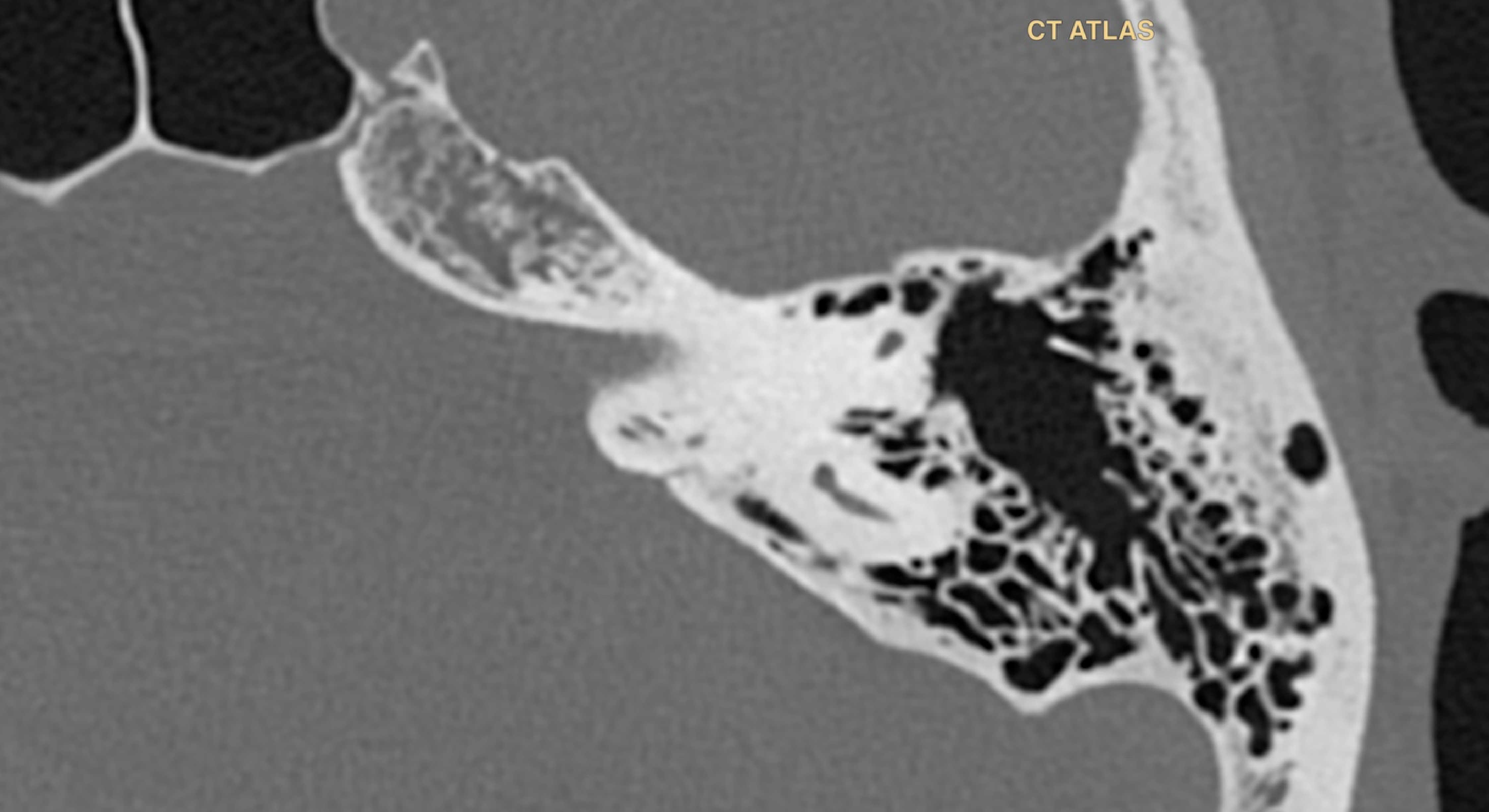
CT ATLAS

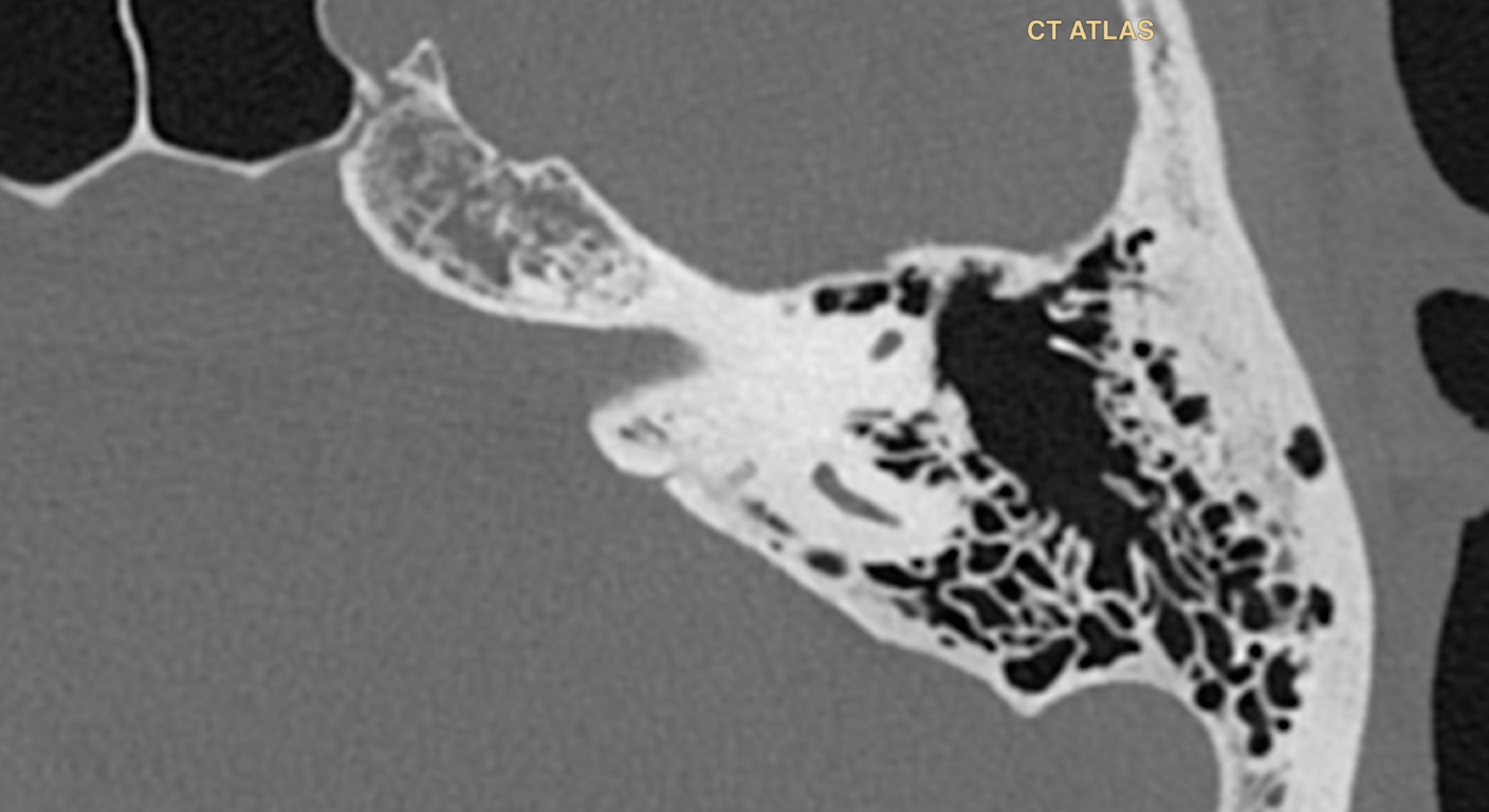
CT ATLAS

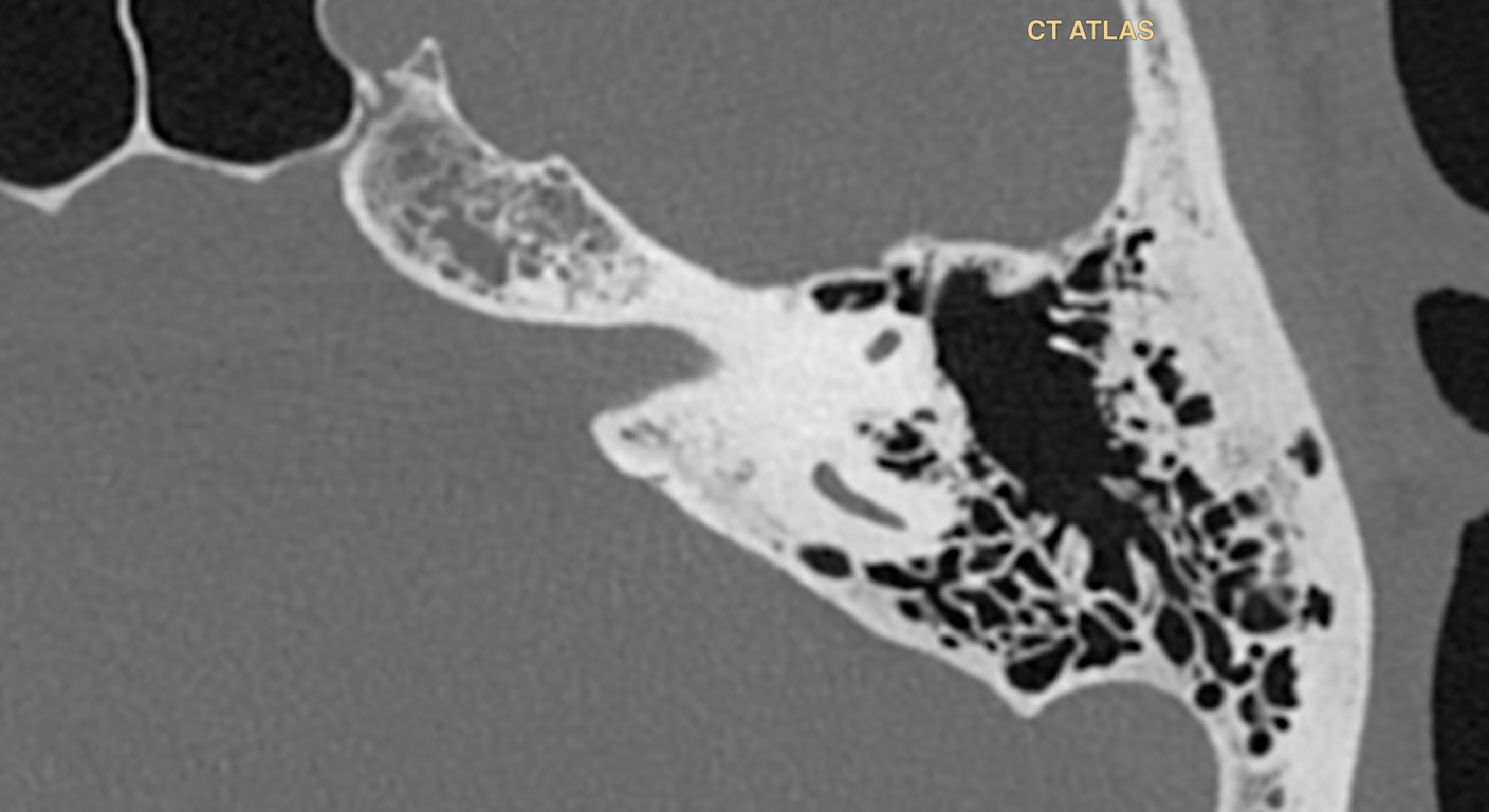
CT ATLAS

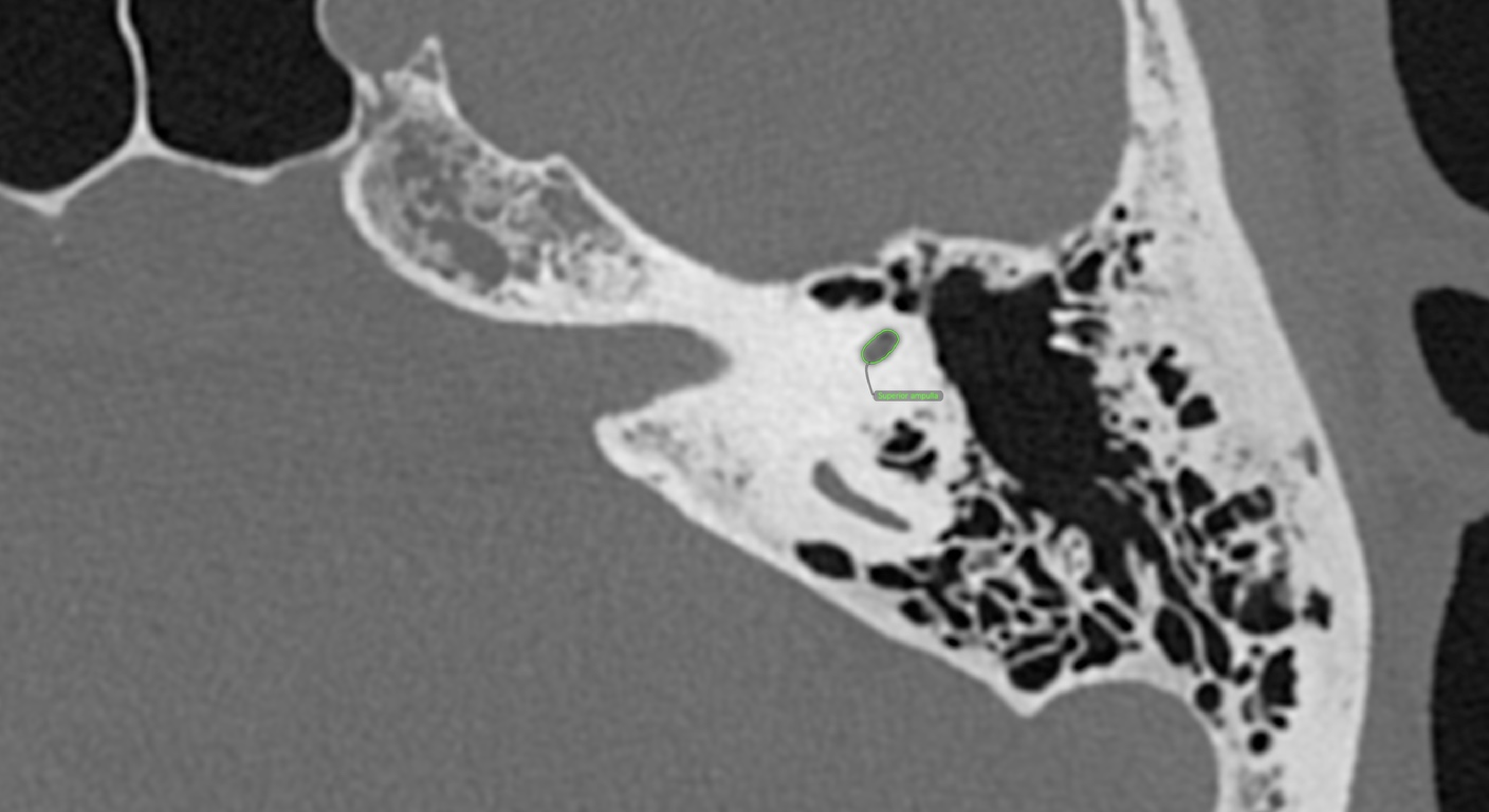
Superior ampulla

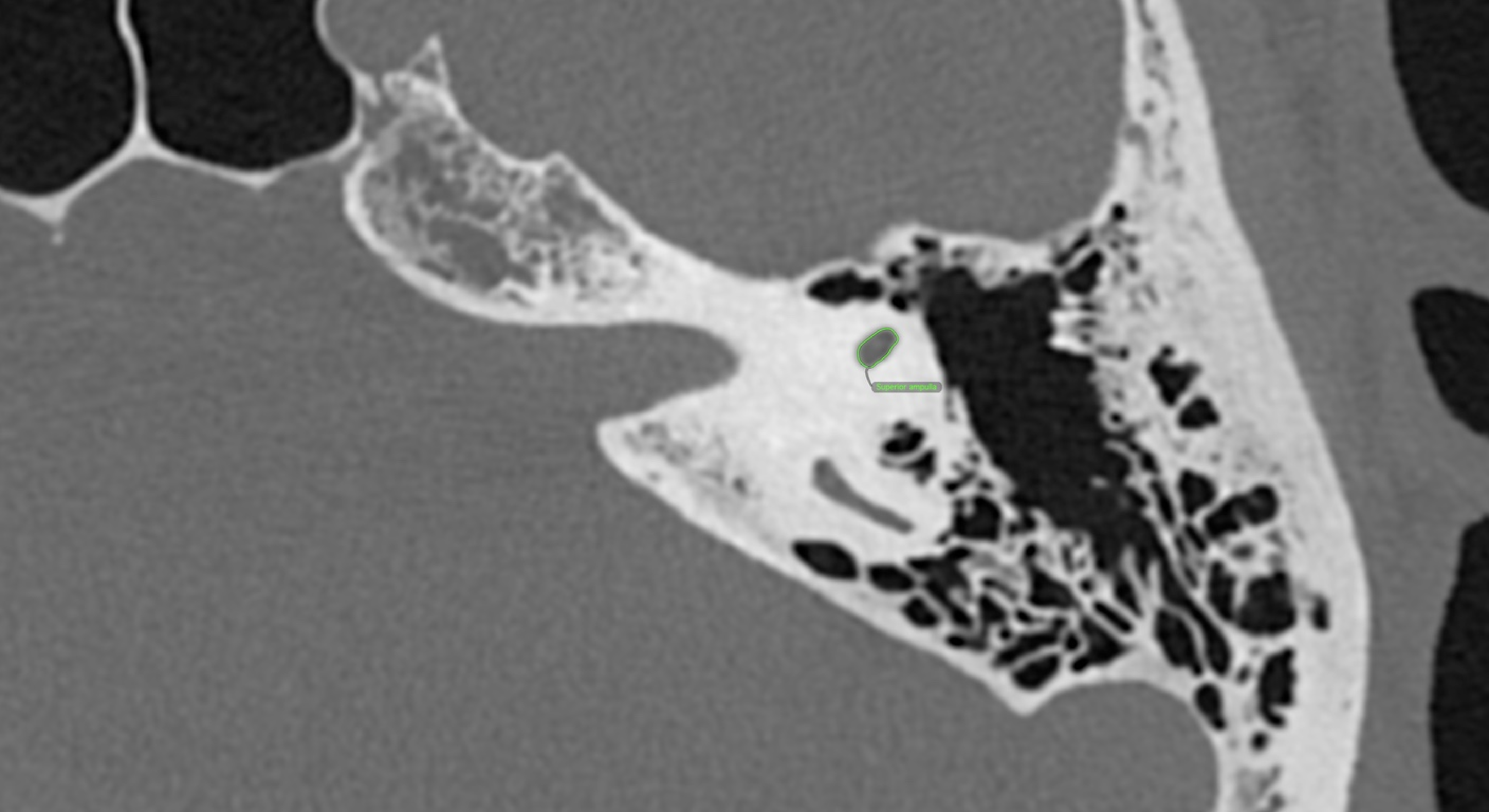
Superior ampulla

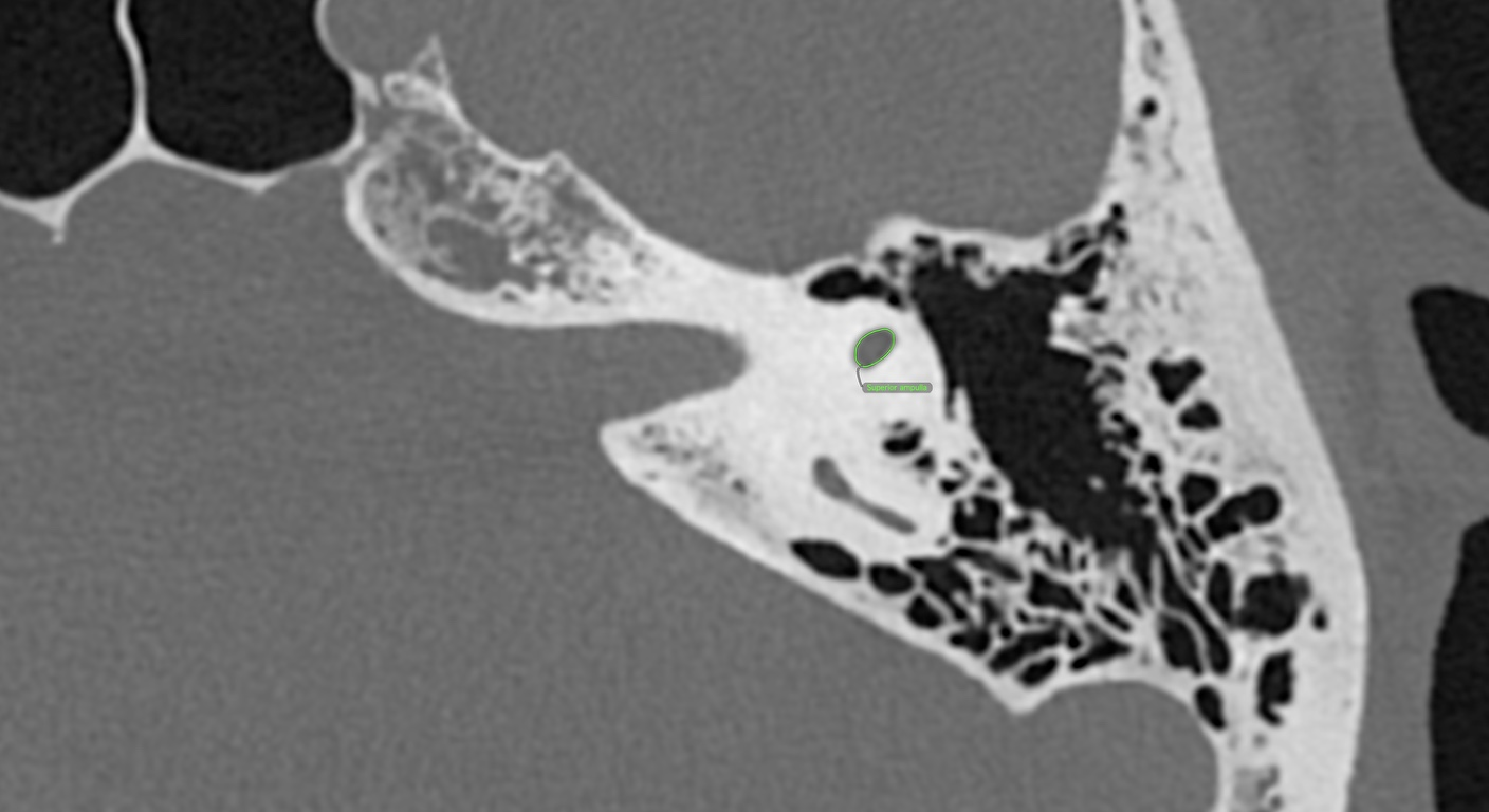
Superior ampulla

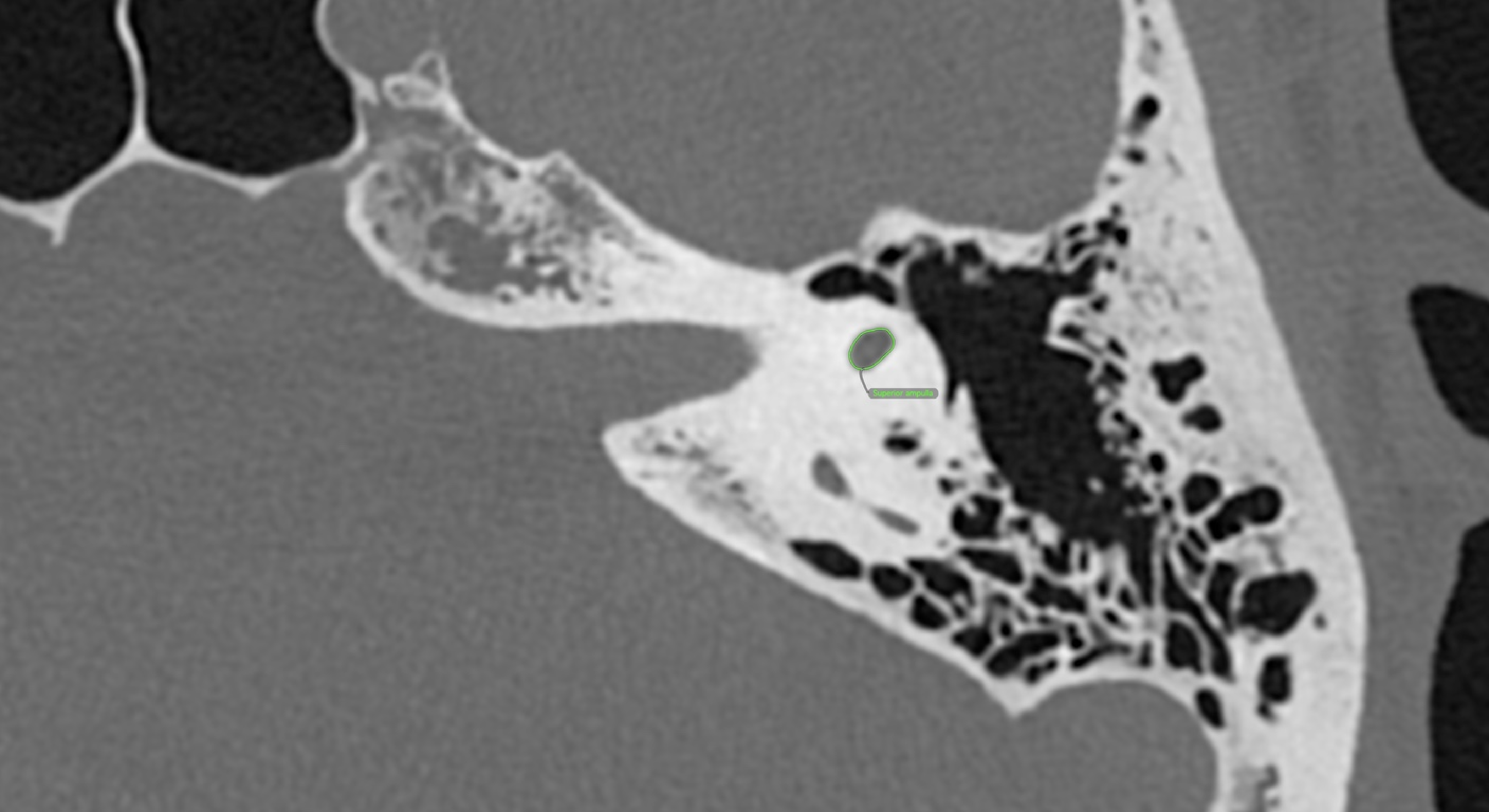
Superior ampulla

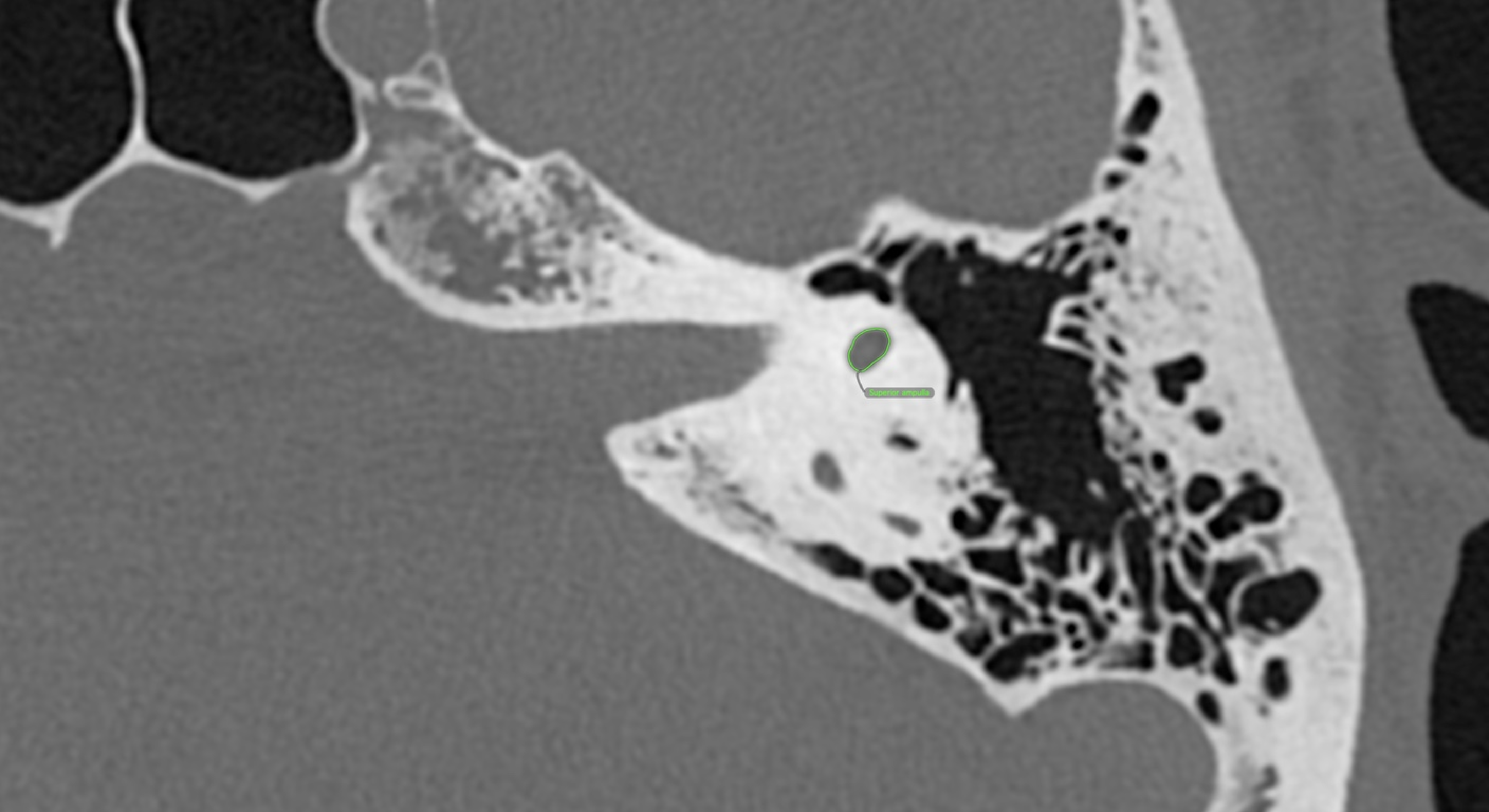
Superior ampulla

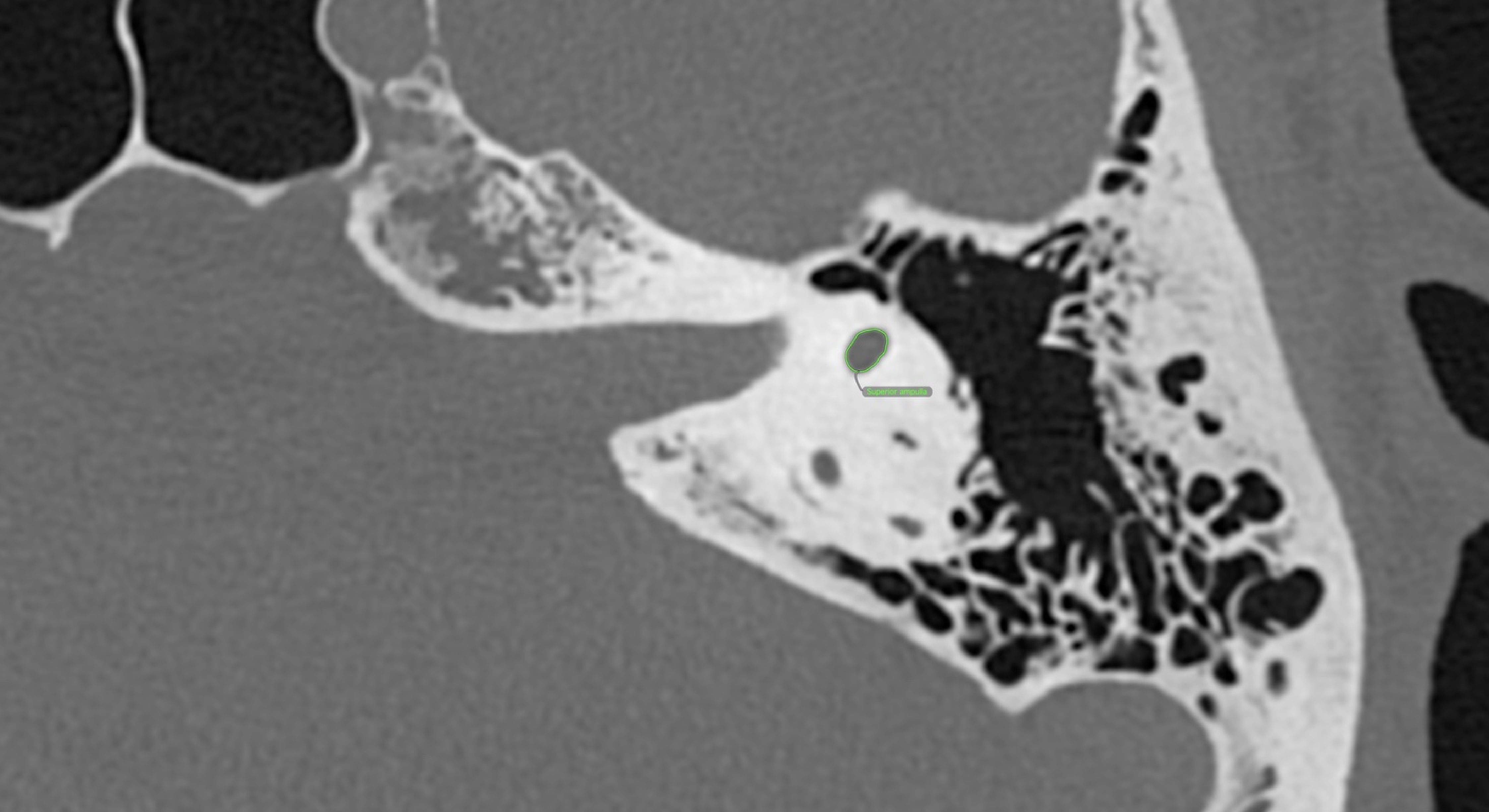
Superior ampulla

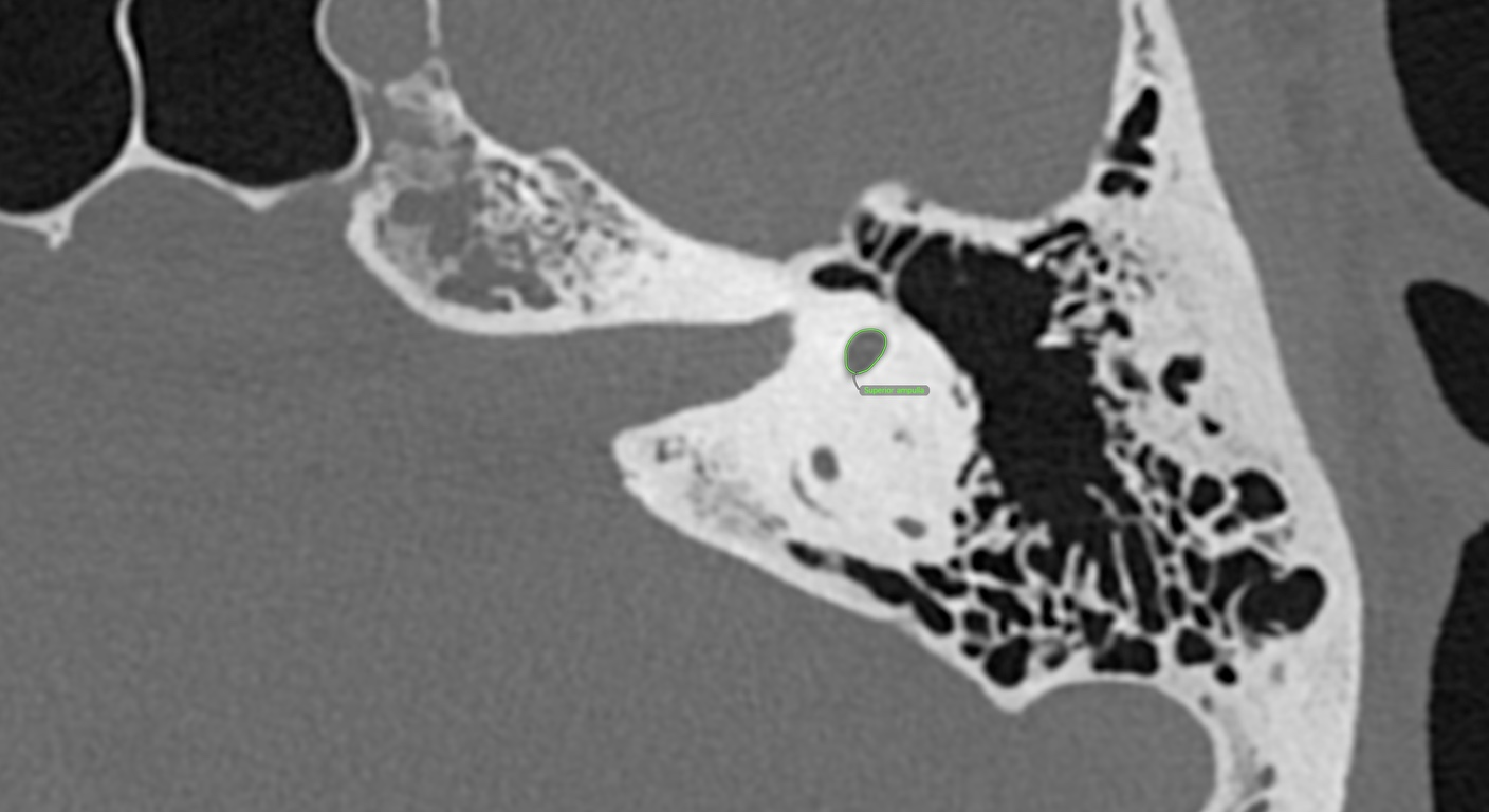
Superior ampulla

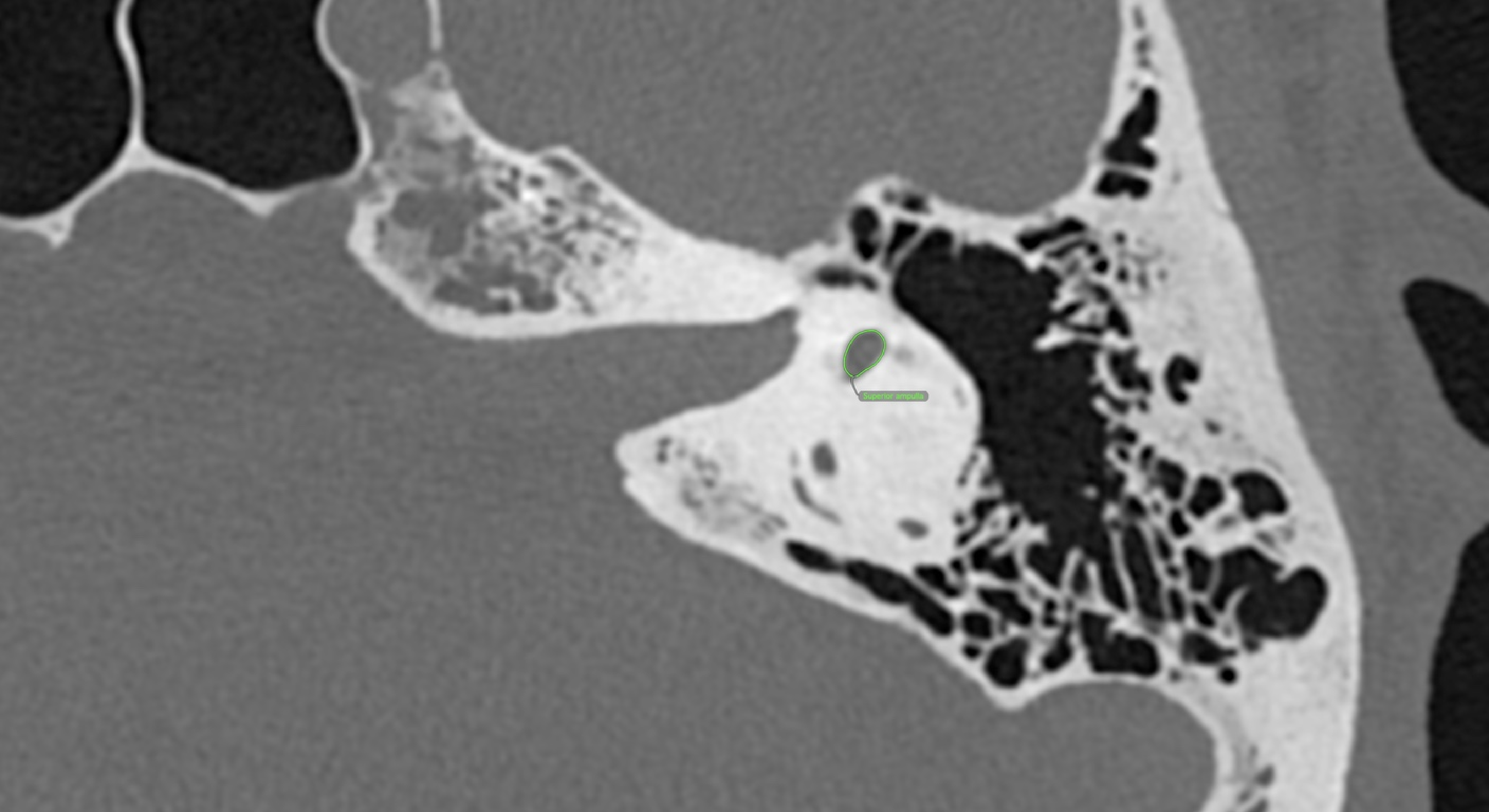
Superior ampulla

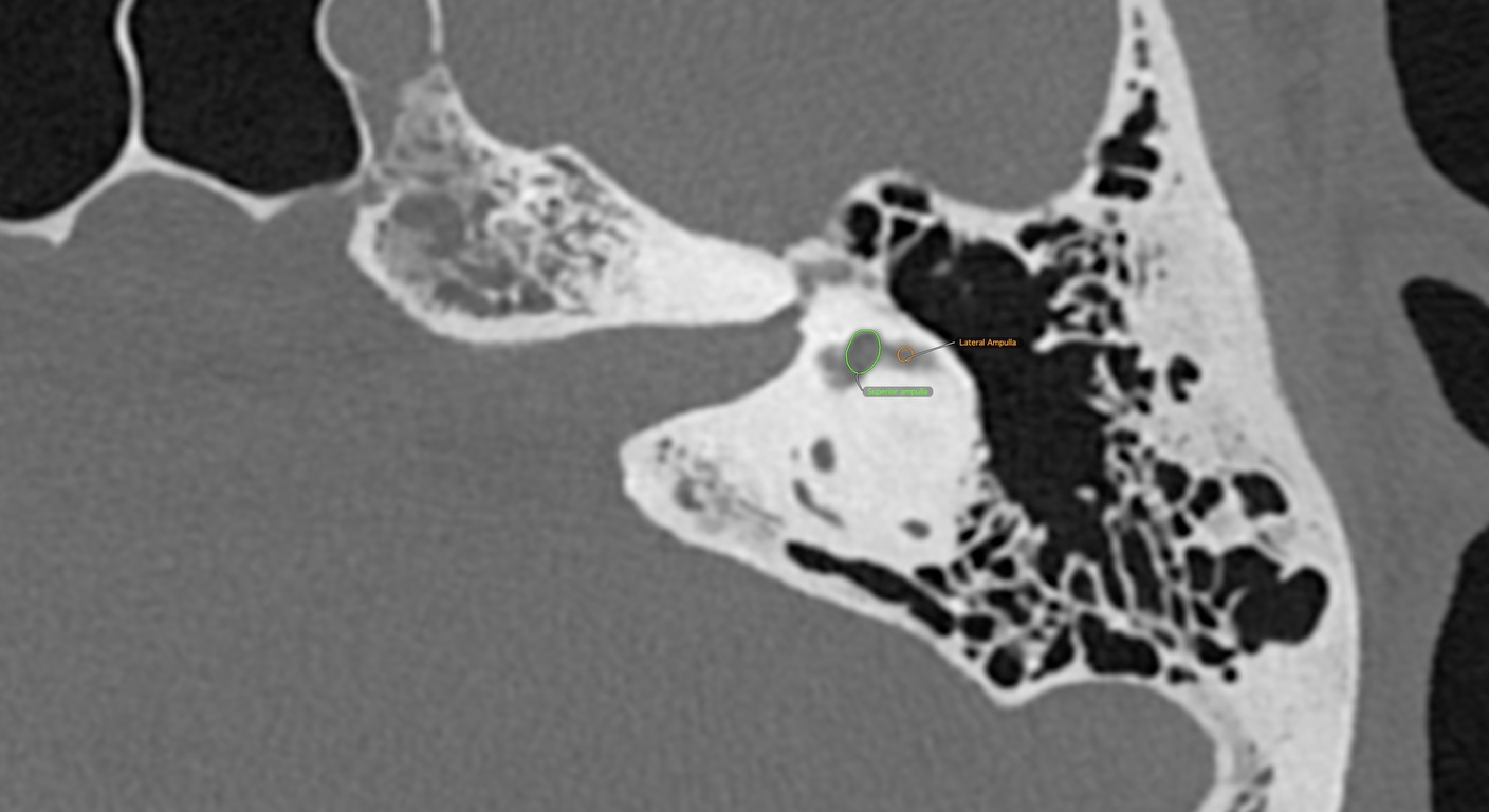
Lateral Ampulla
Superior ampulla

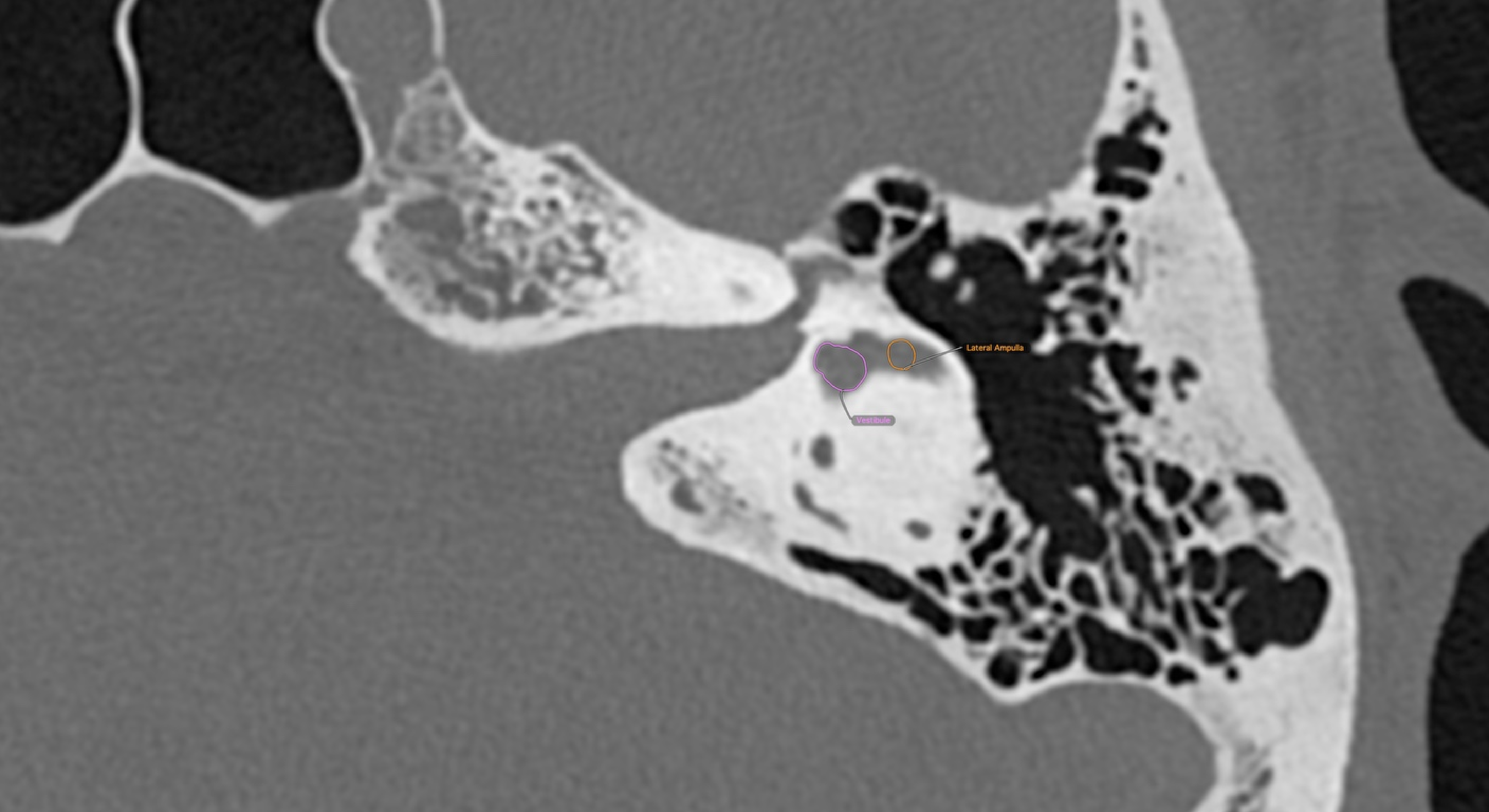
Lateral Ampulla
Vestibule

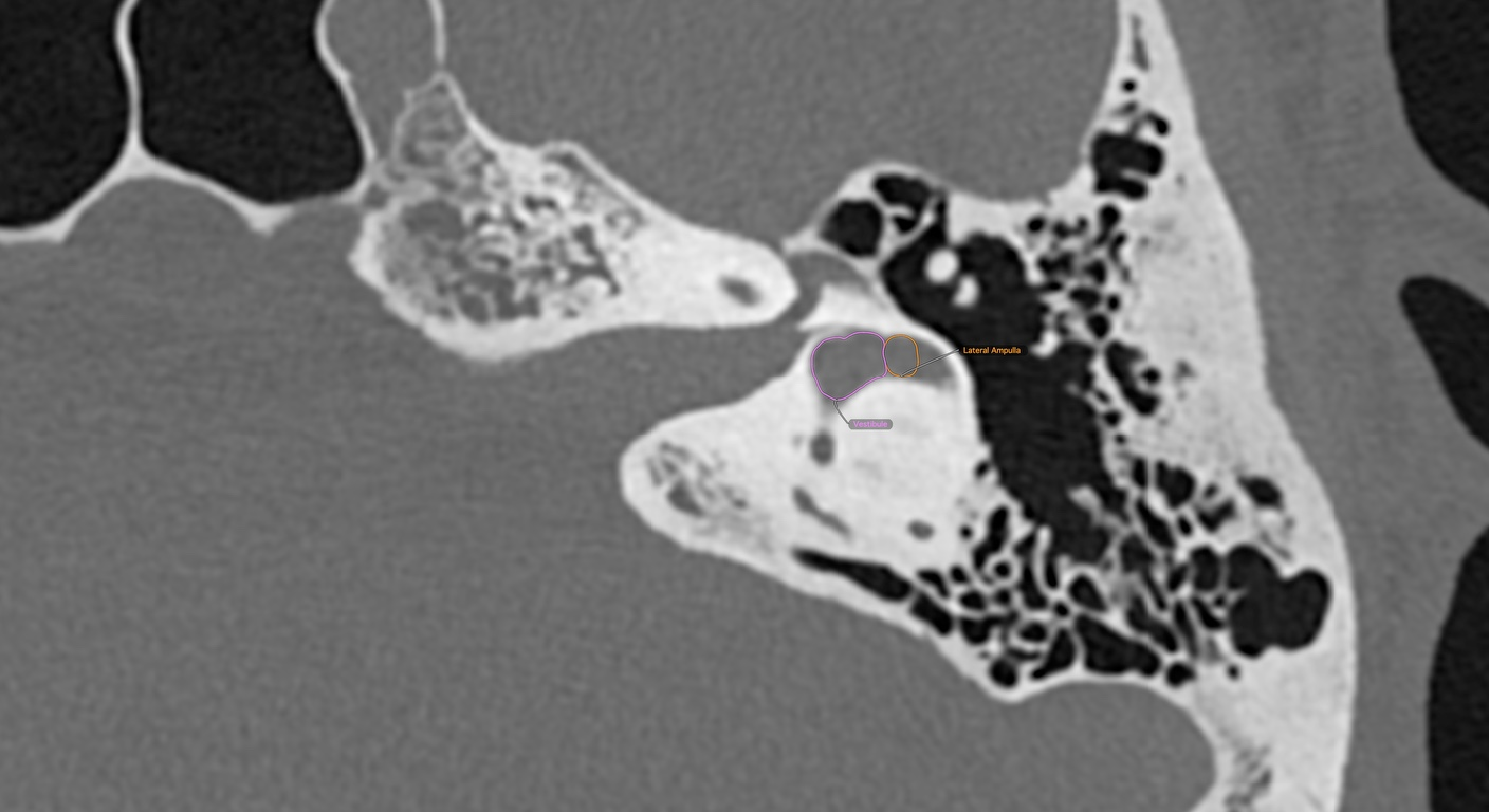
Lateral Ampulla
Vestibule

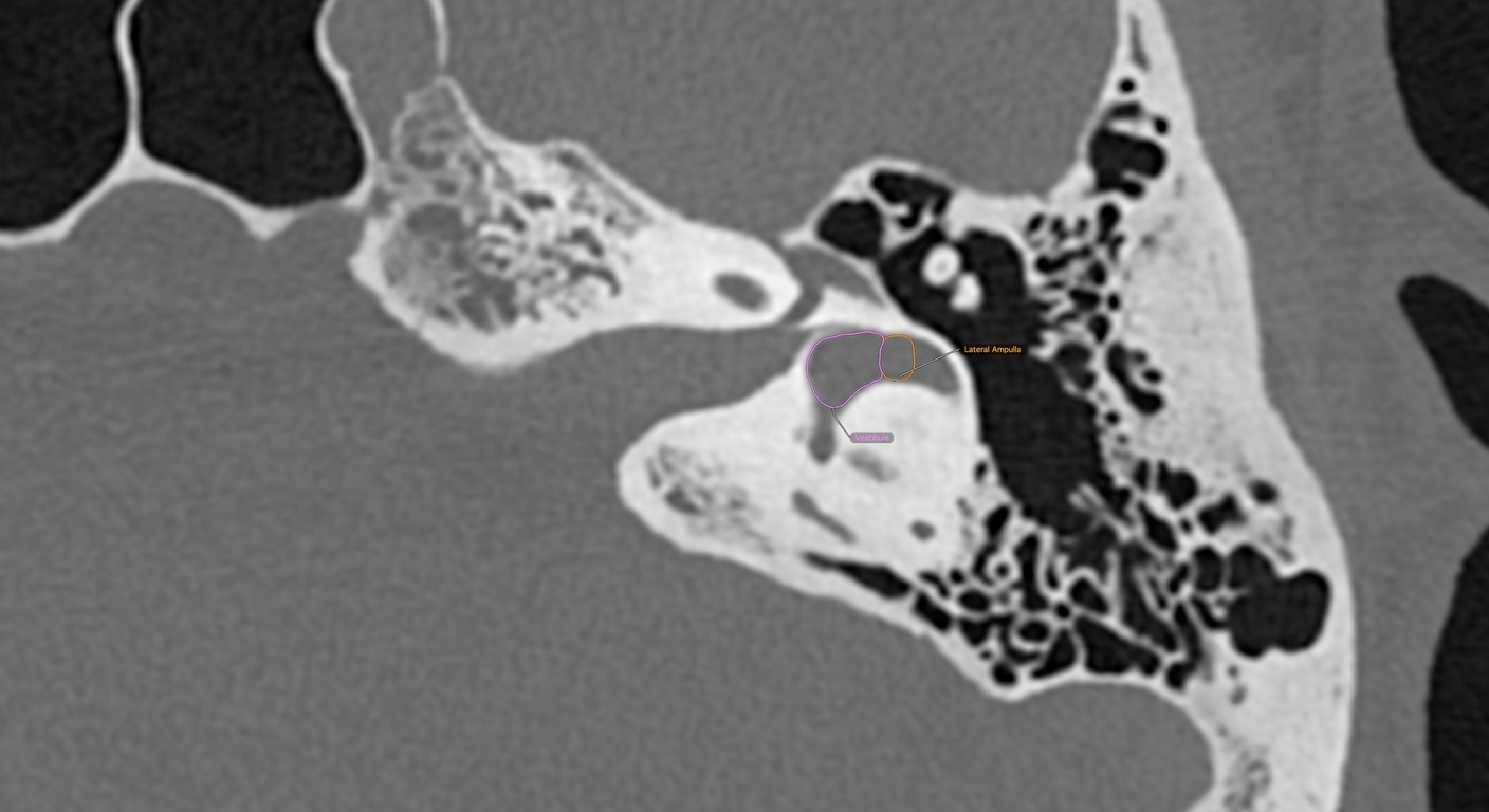

Lateral Ampulla
Vestibule

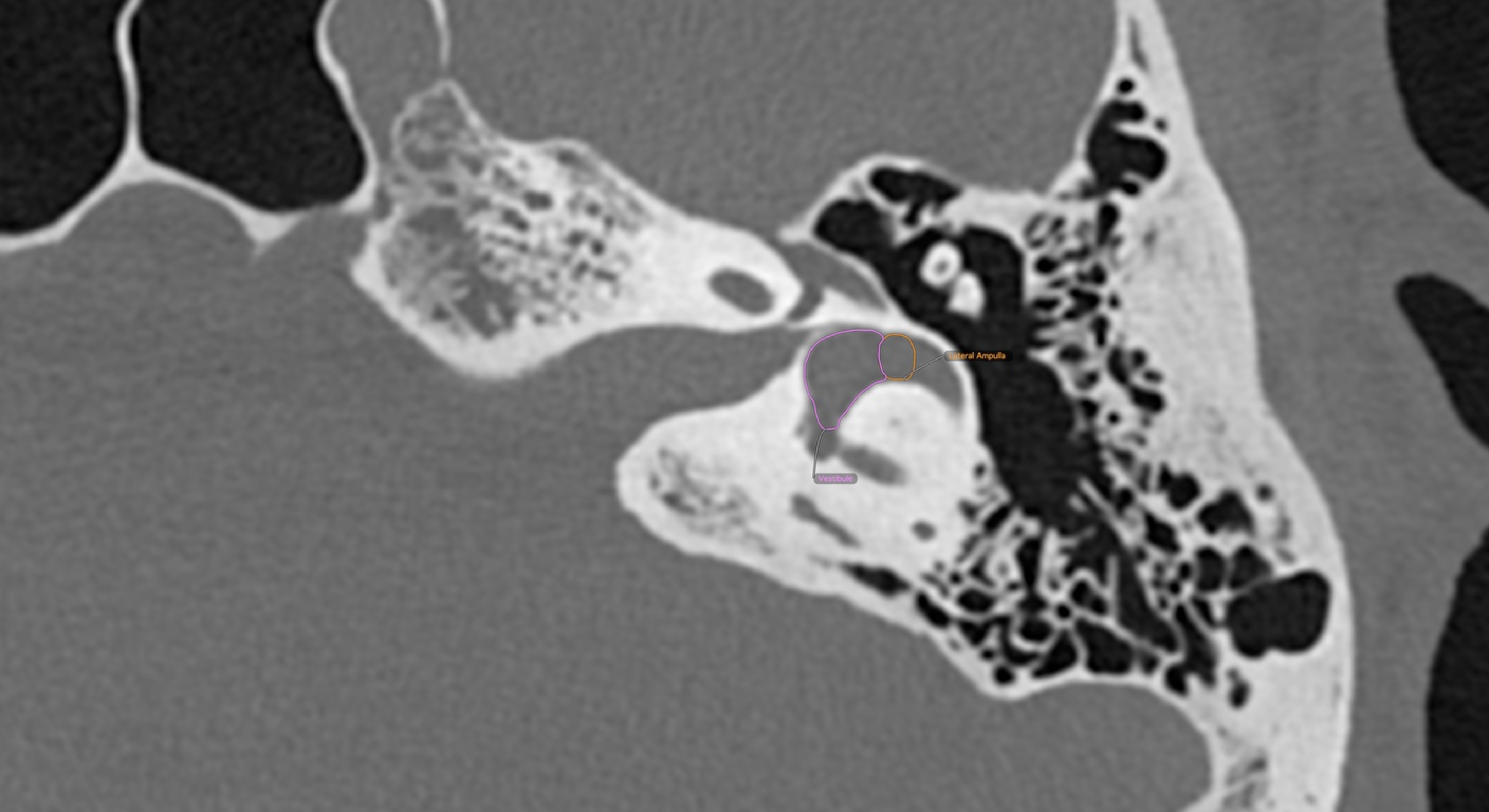
Lateral Ampulla
Vestibule

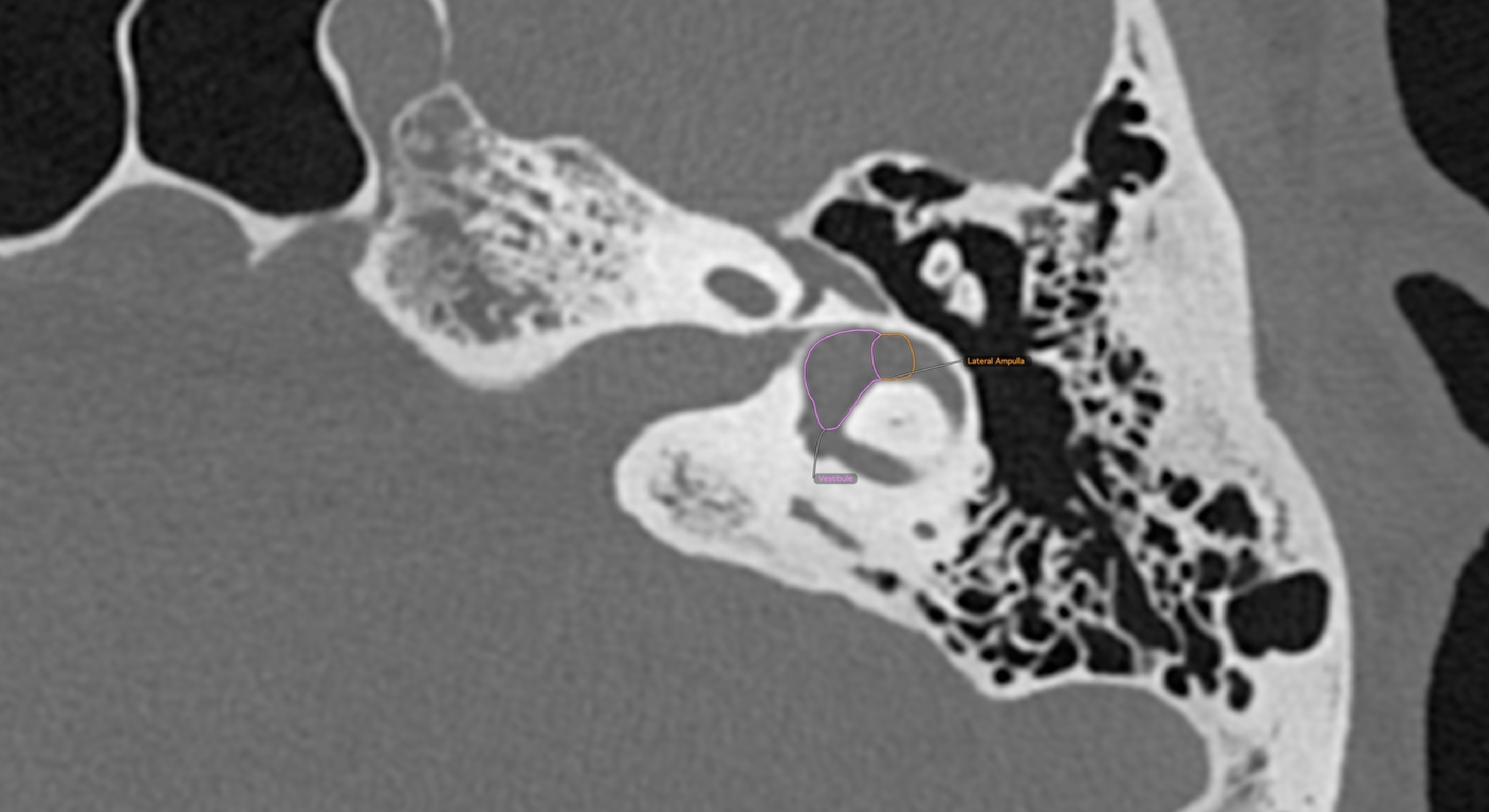
Lateral Ampulla
Vestibule

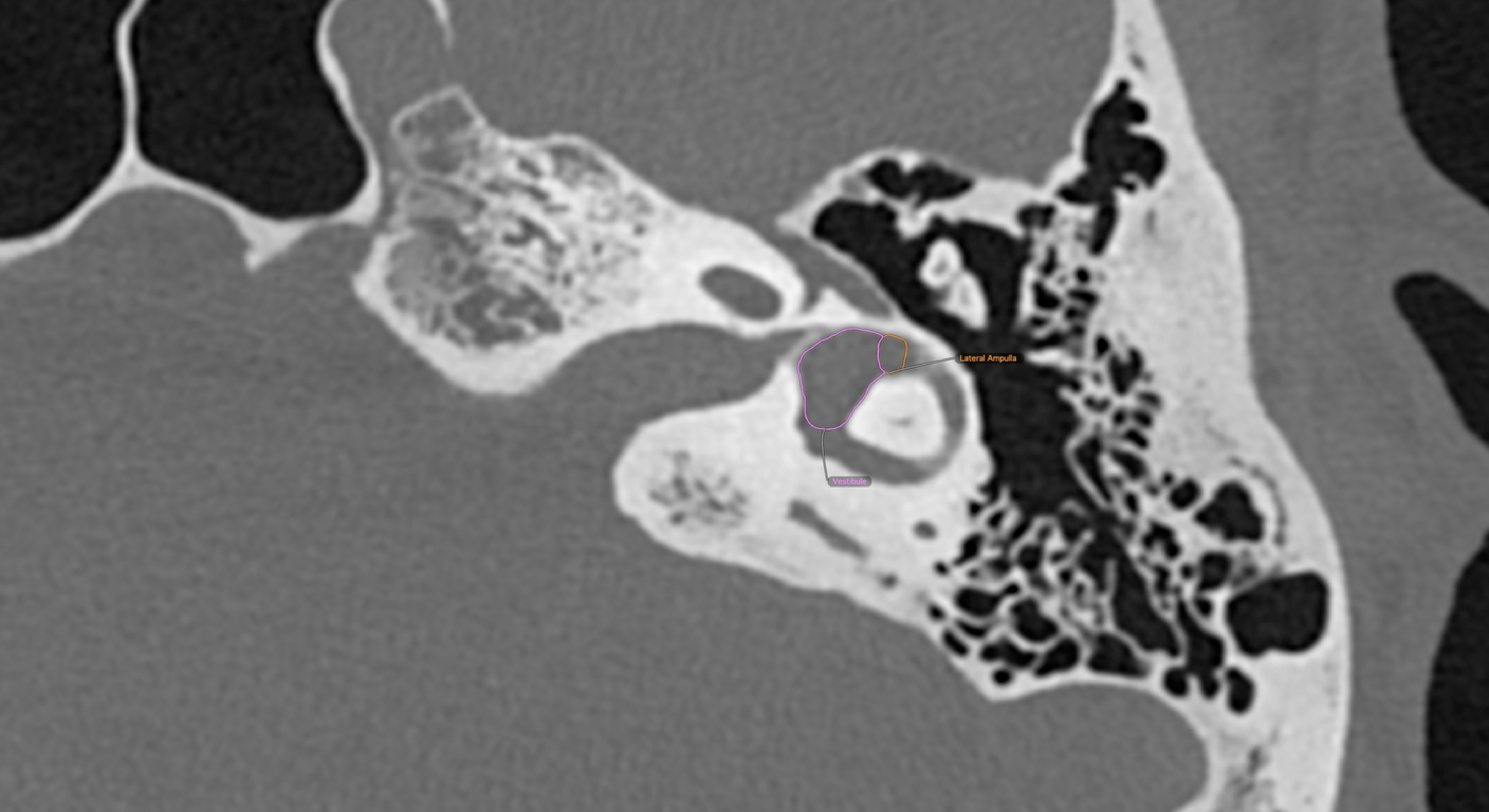
Lateral Ampulla
Vestibule

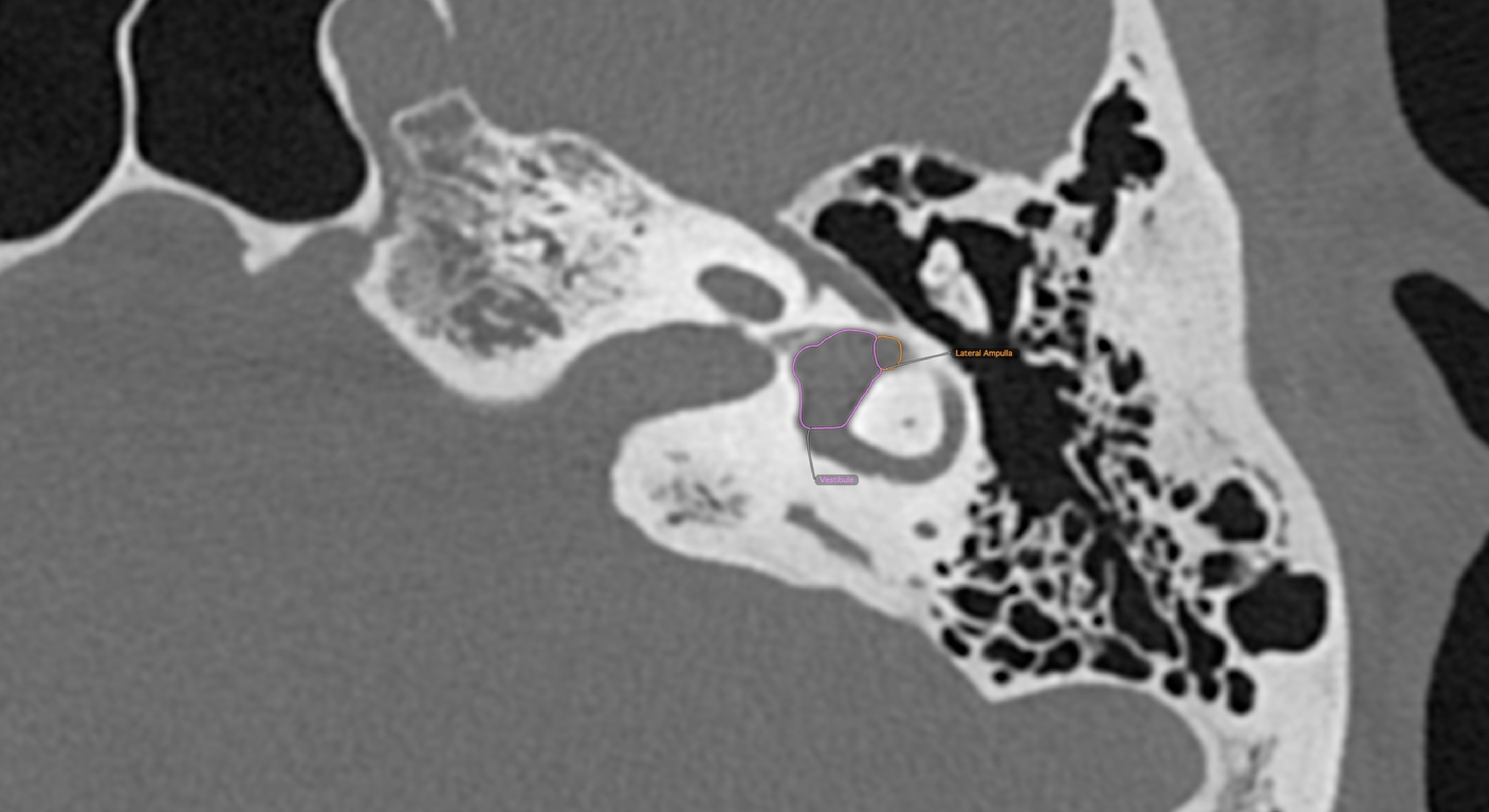
Lateral Ampulla
Vestibule

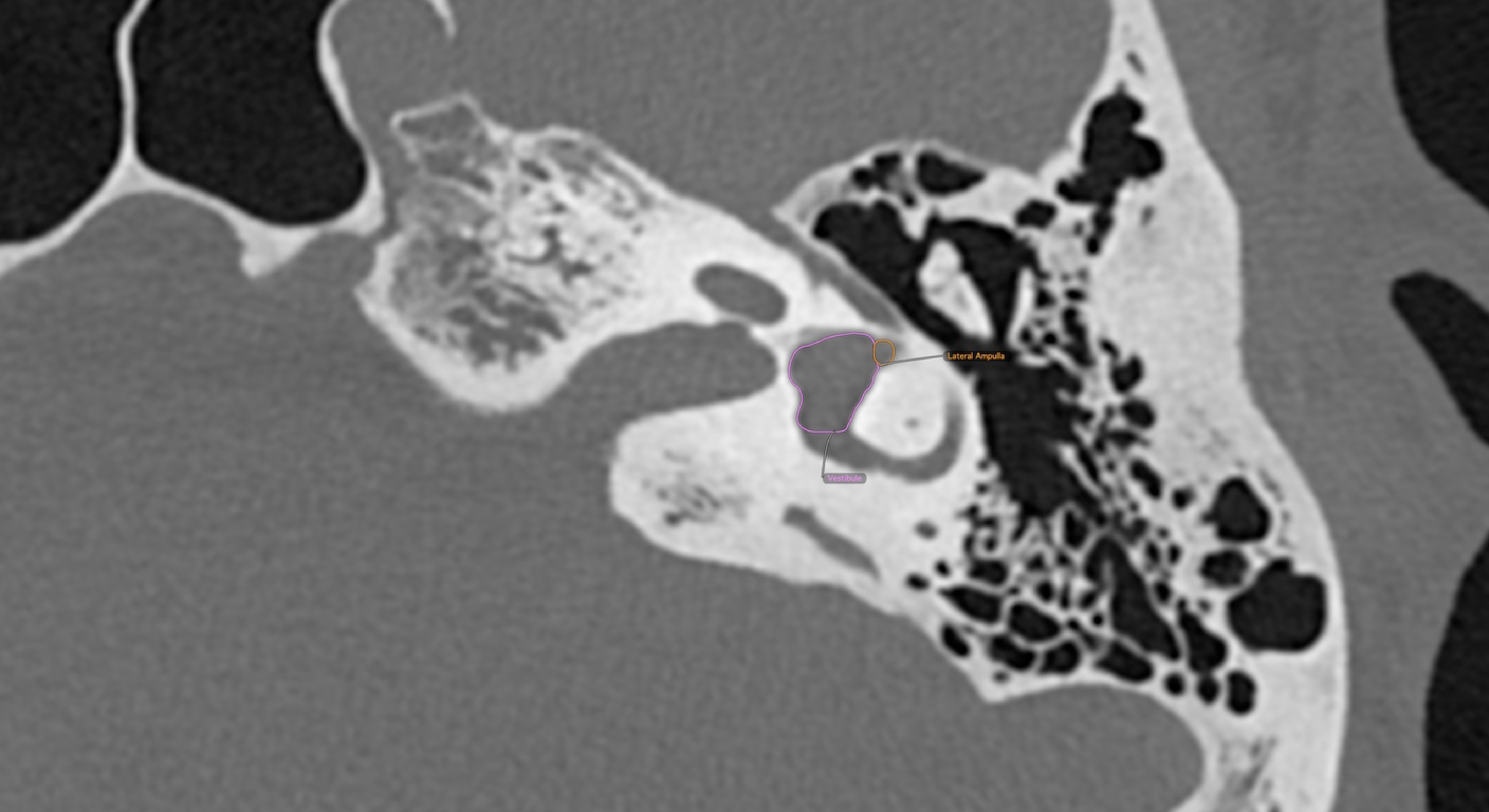
Lateral Ampulla
Vestibule

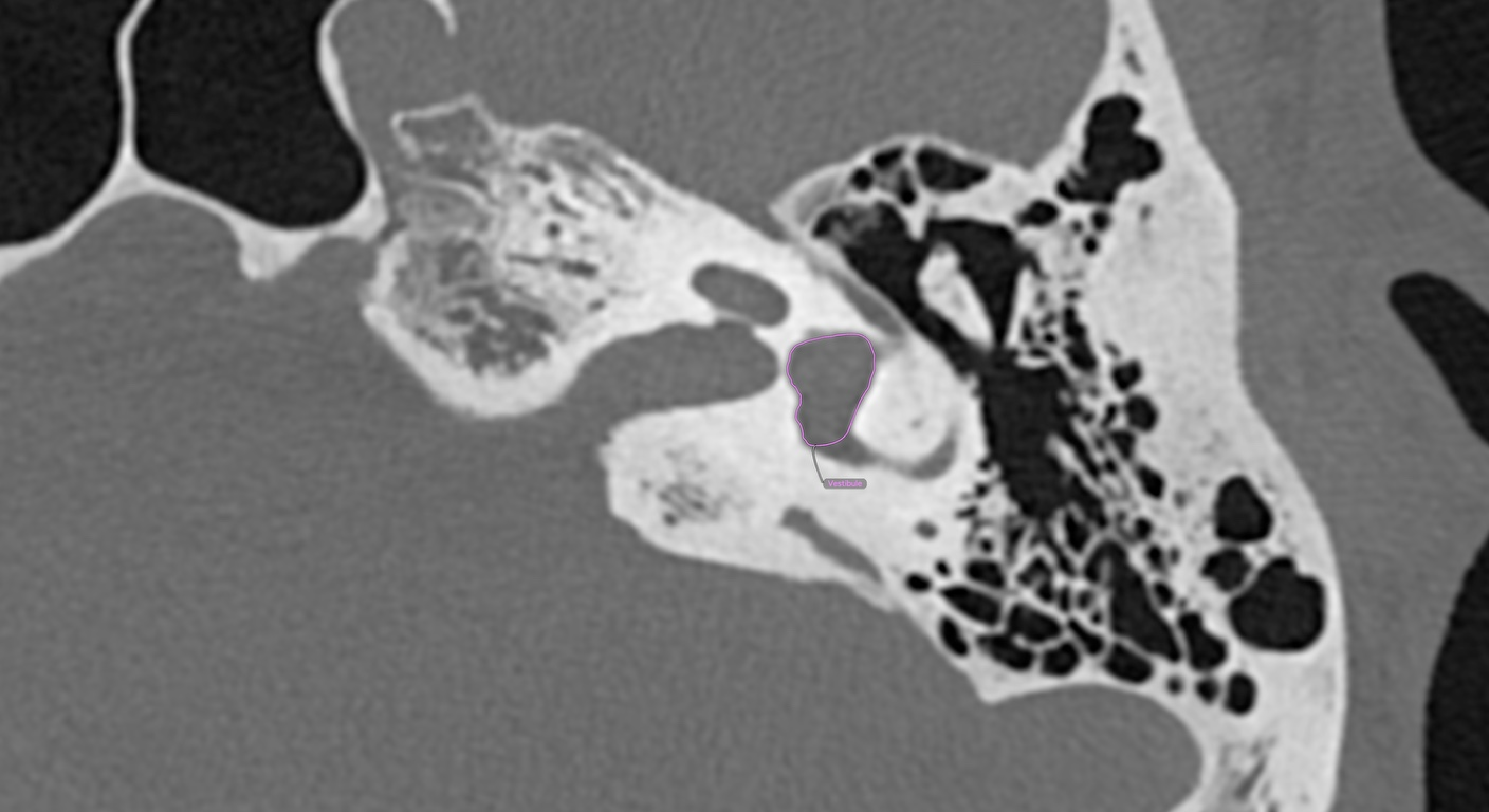
Vestibule

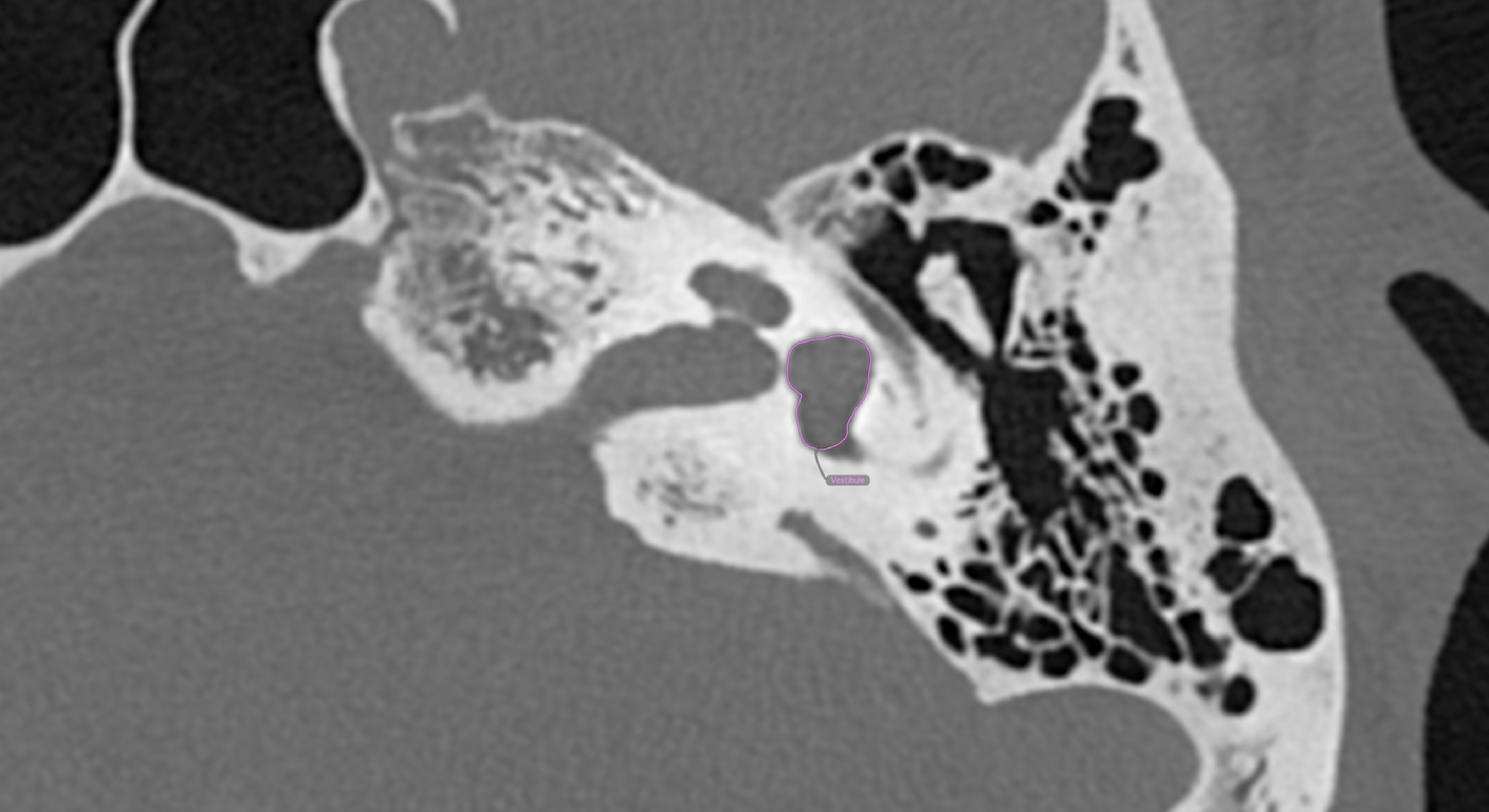
Vestibule

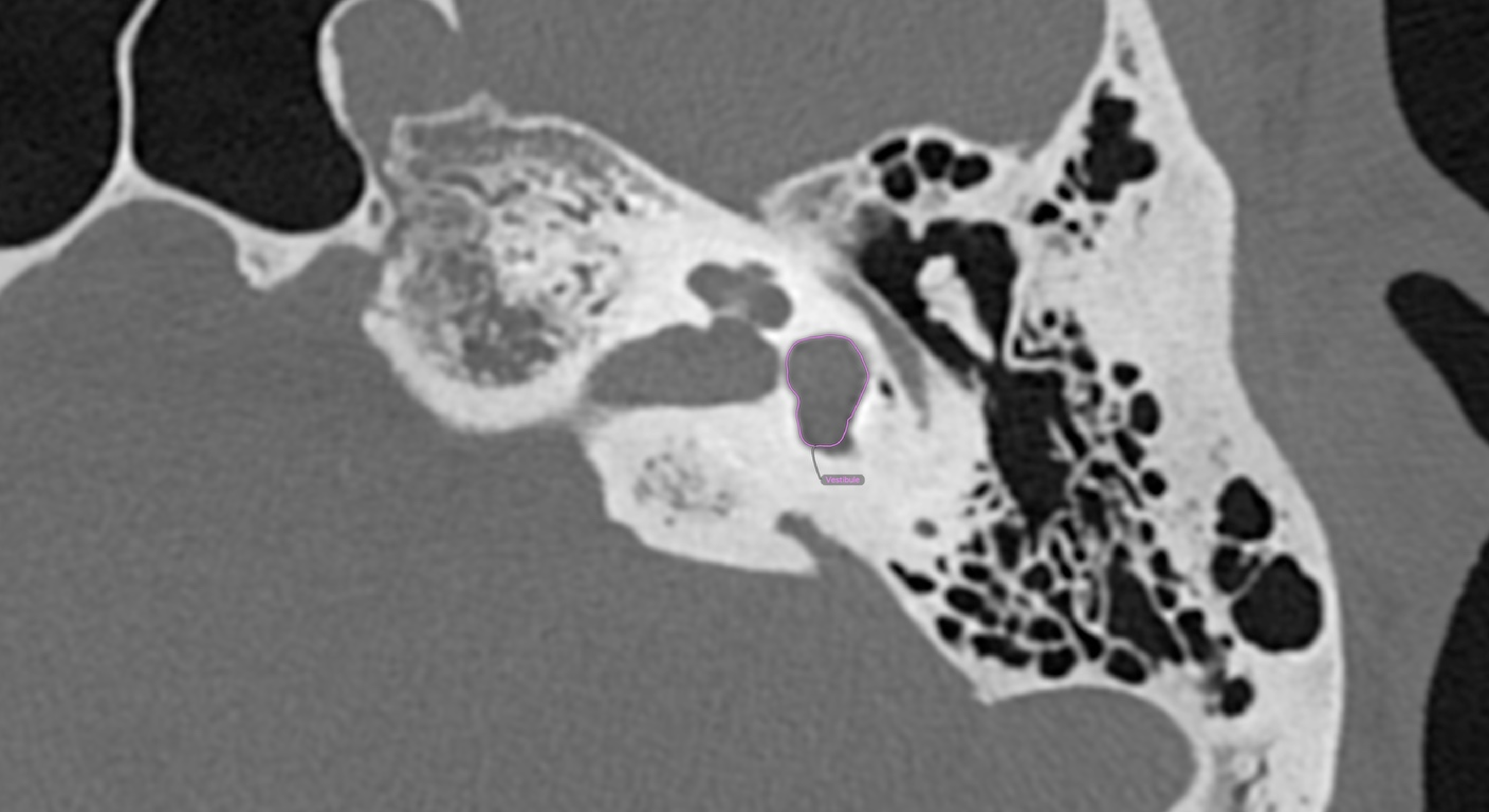
Vestibule

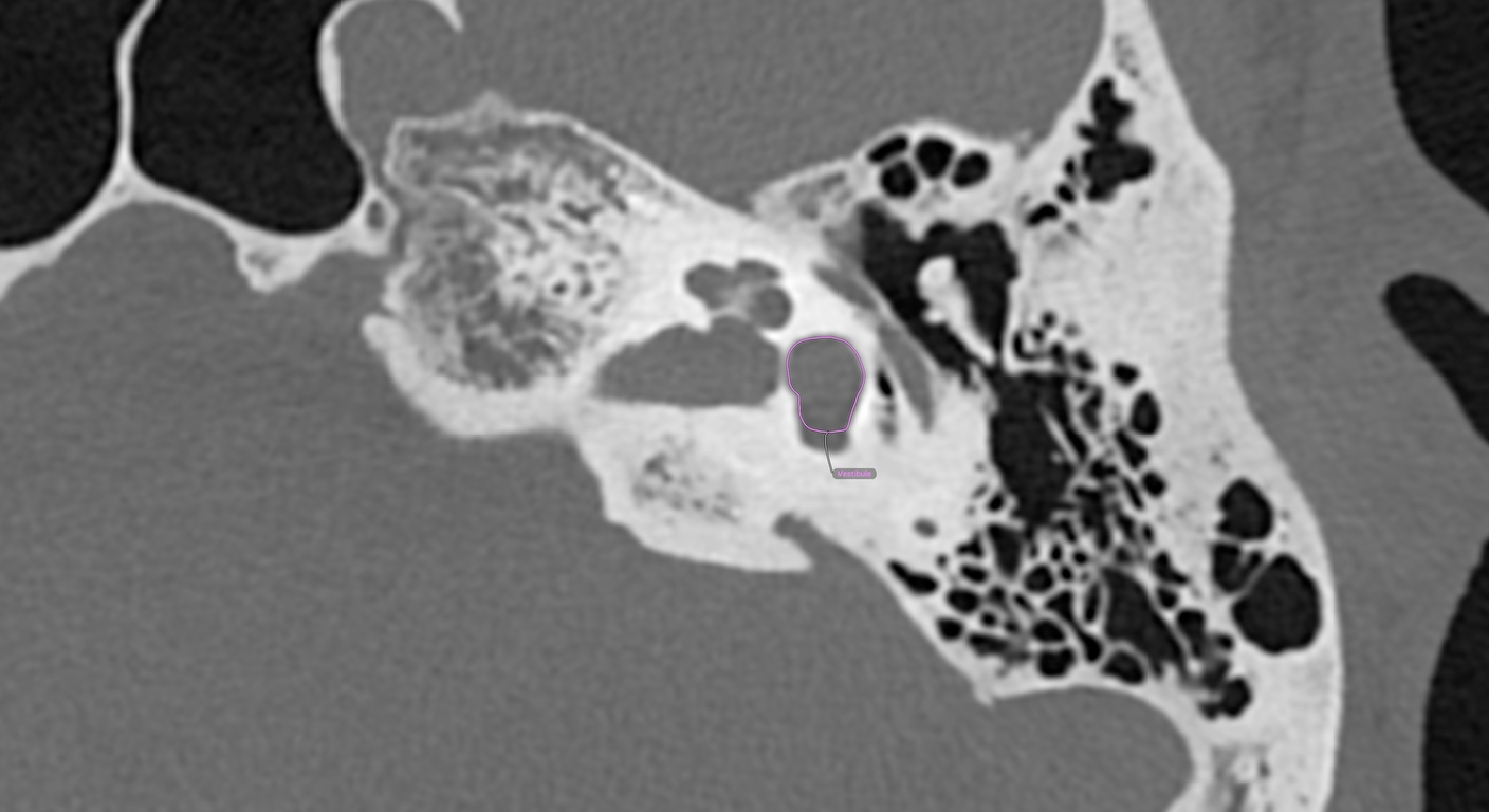
Vestibule

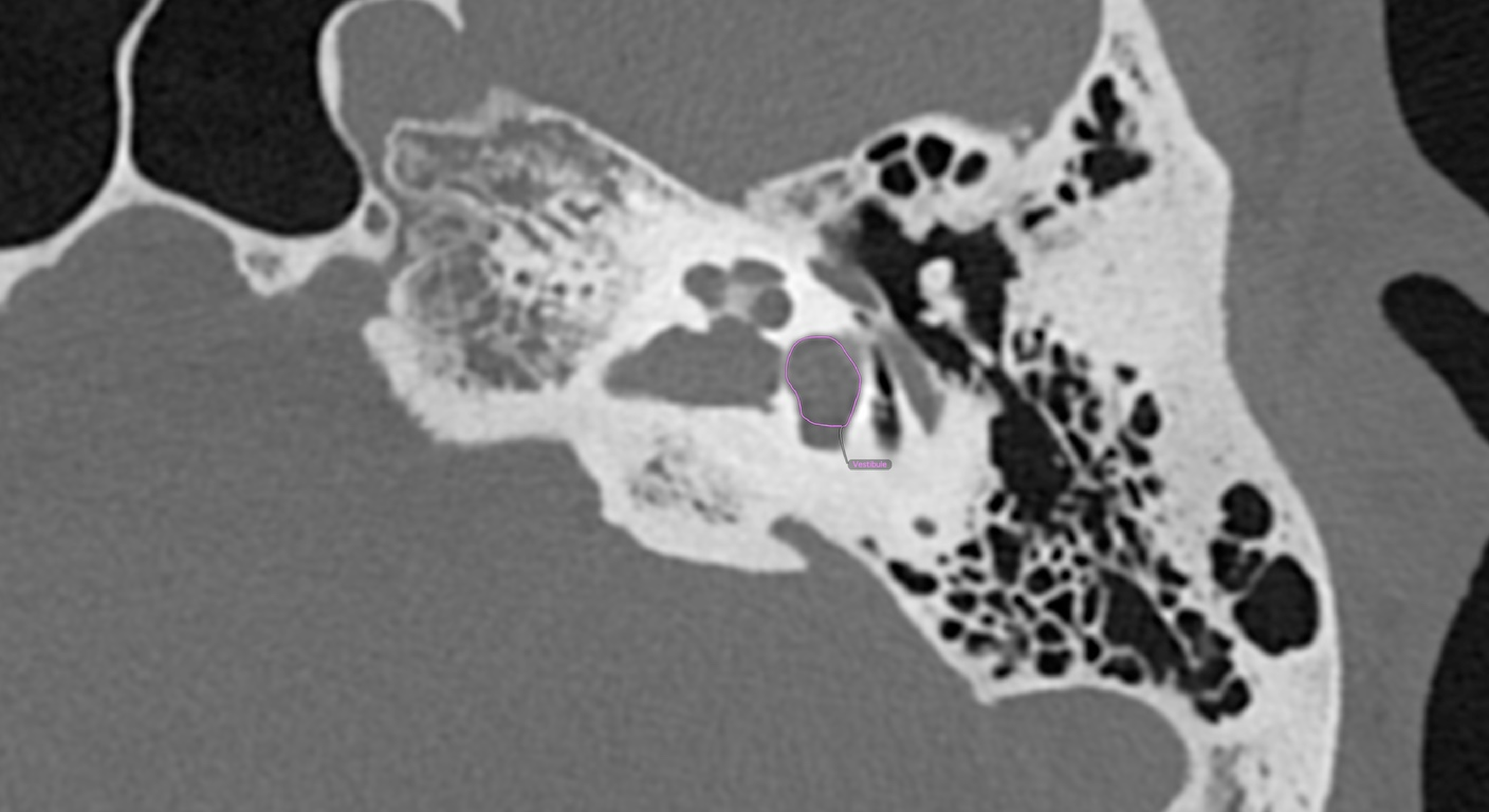
Vestibule

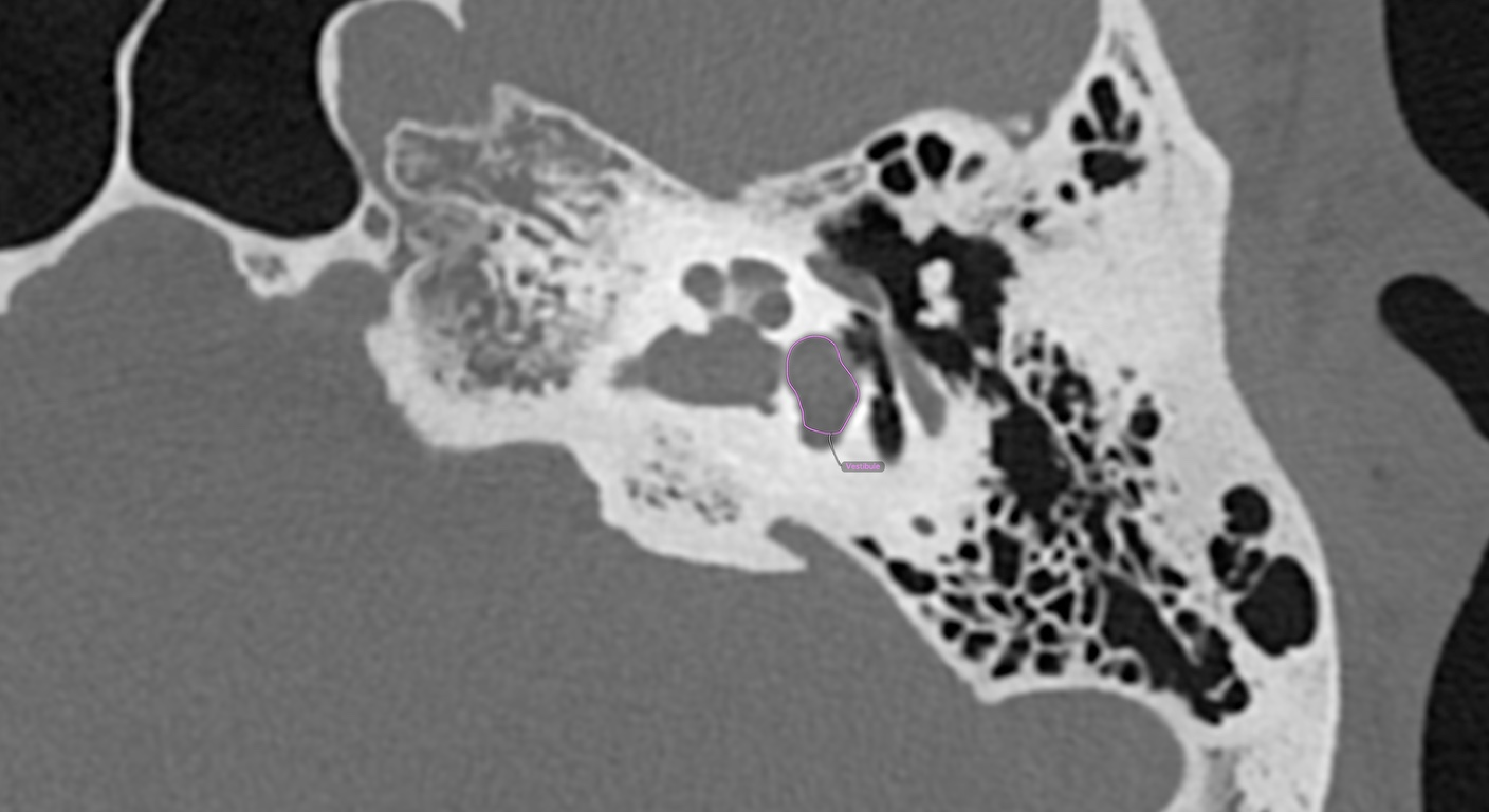
Vestibule

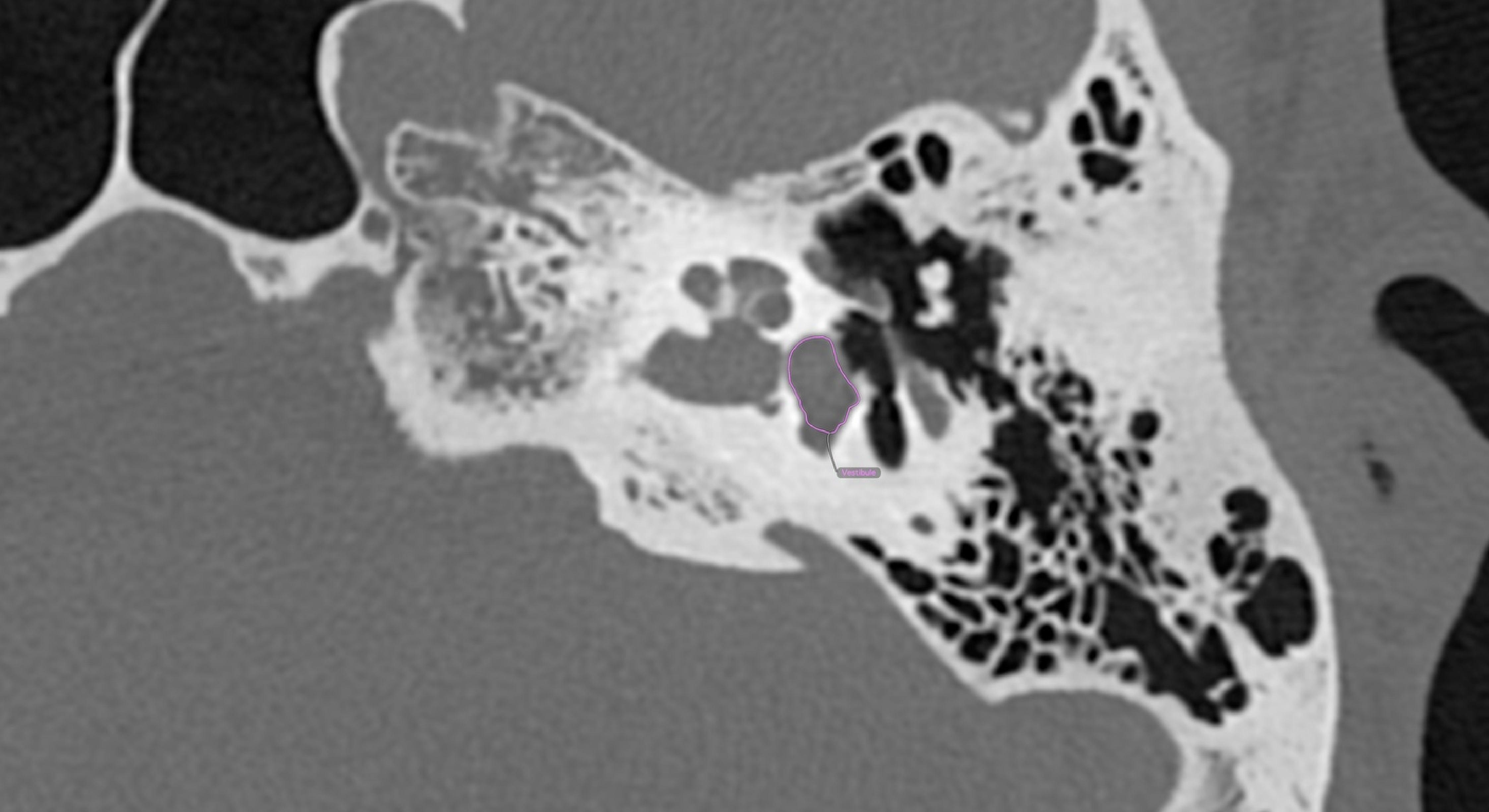
Vestibule

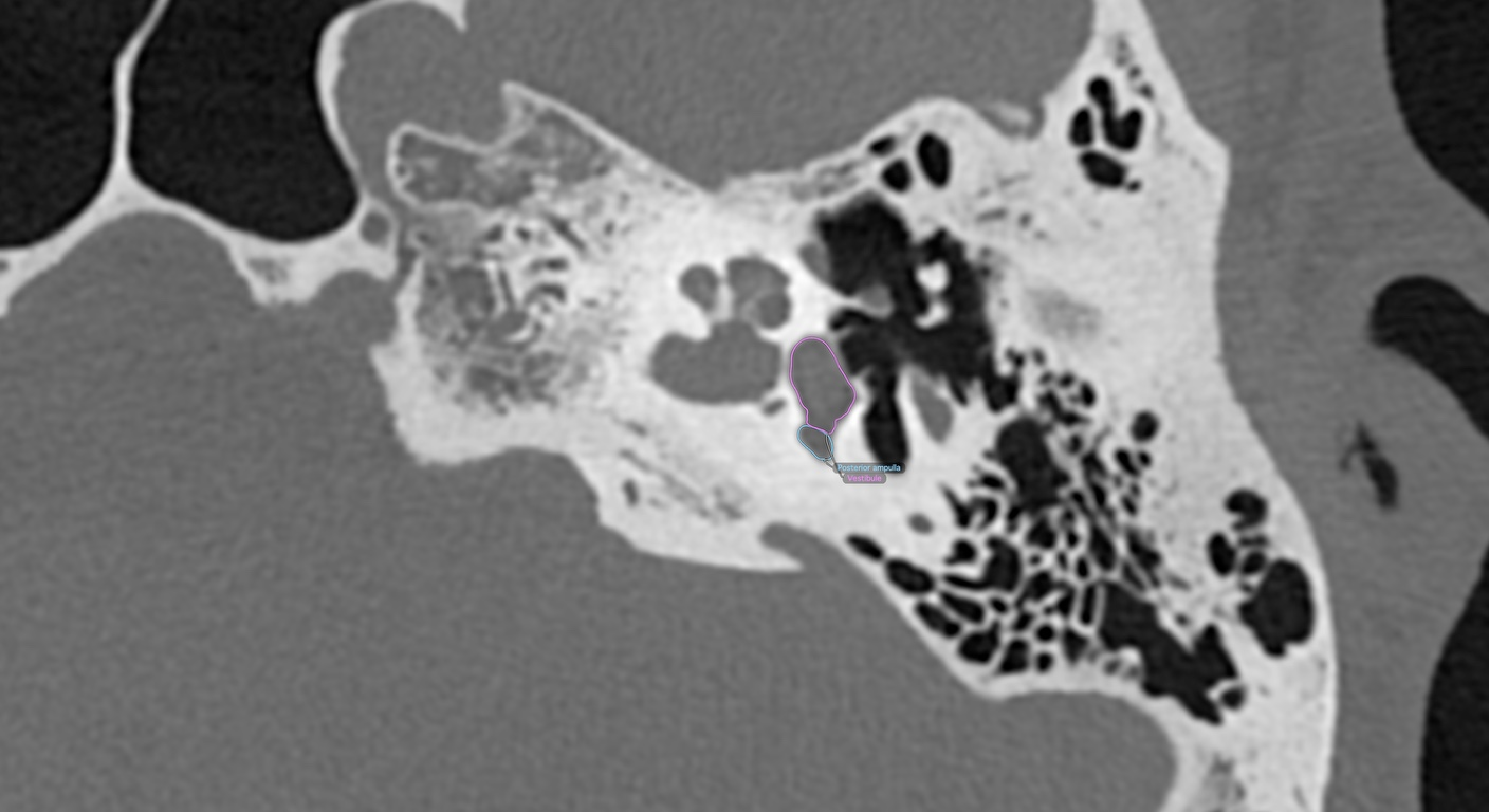
Posterior ampulla
Vestibule

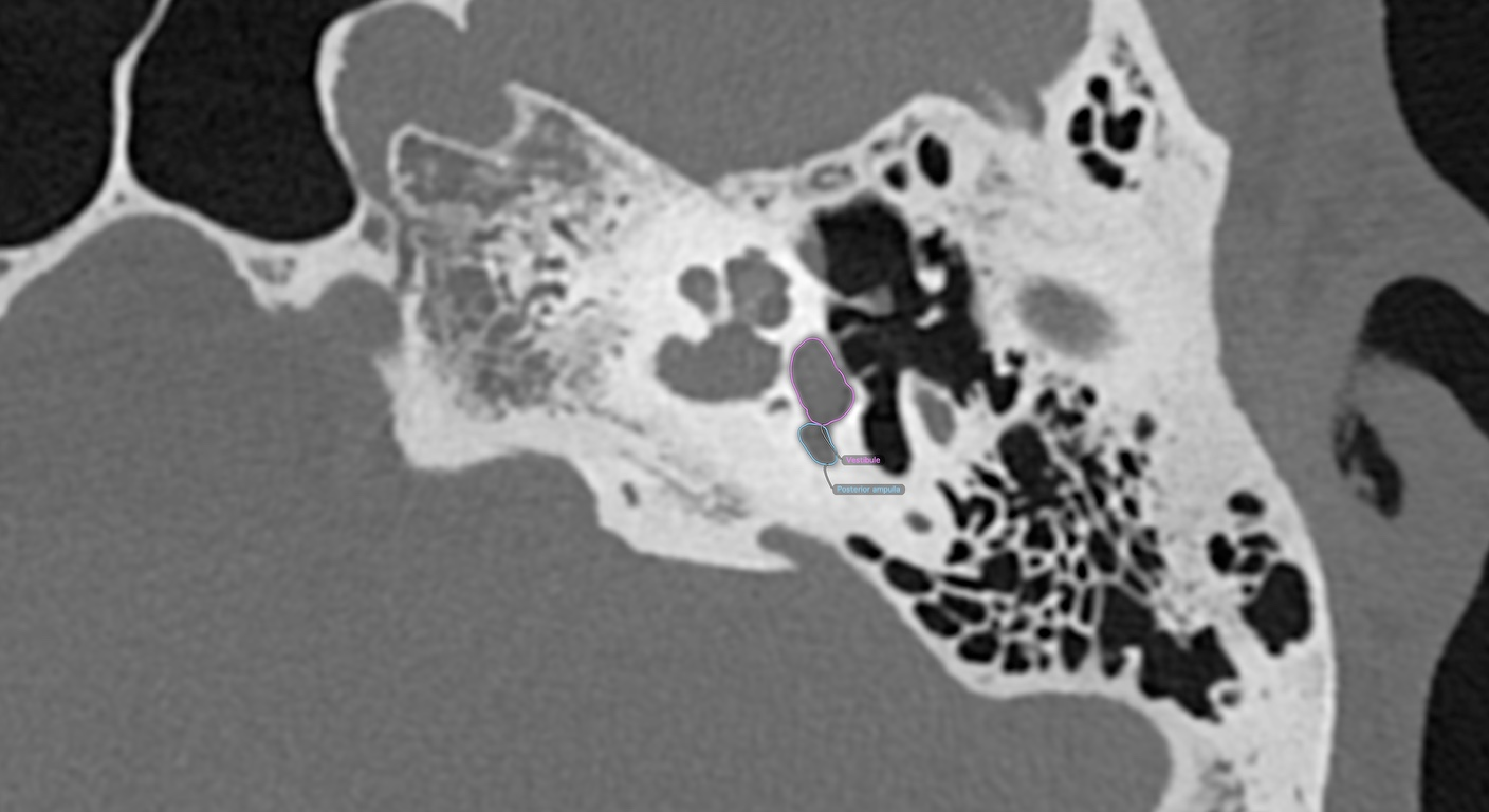
Vestibule
Posterior ampulla

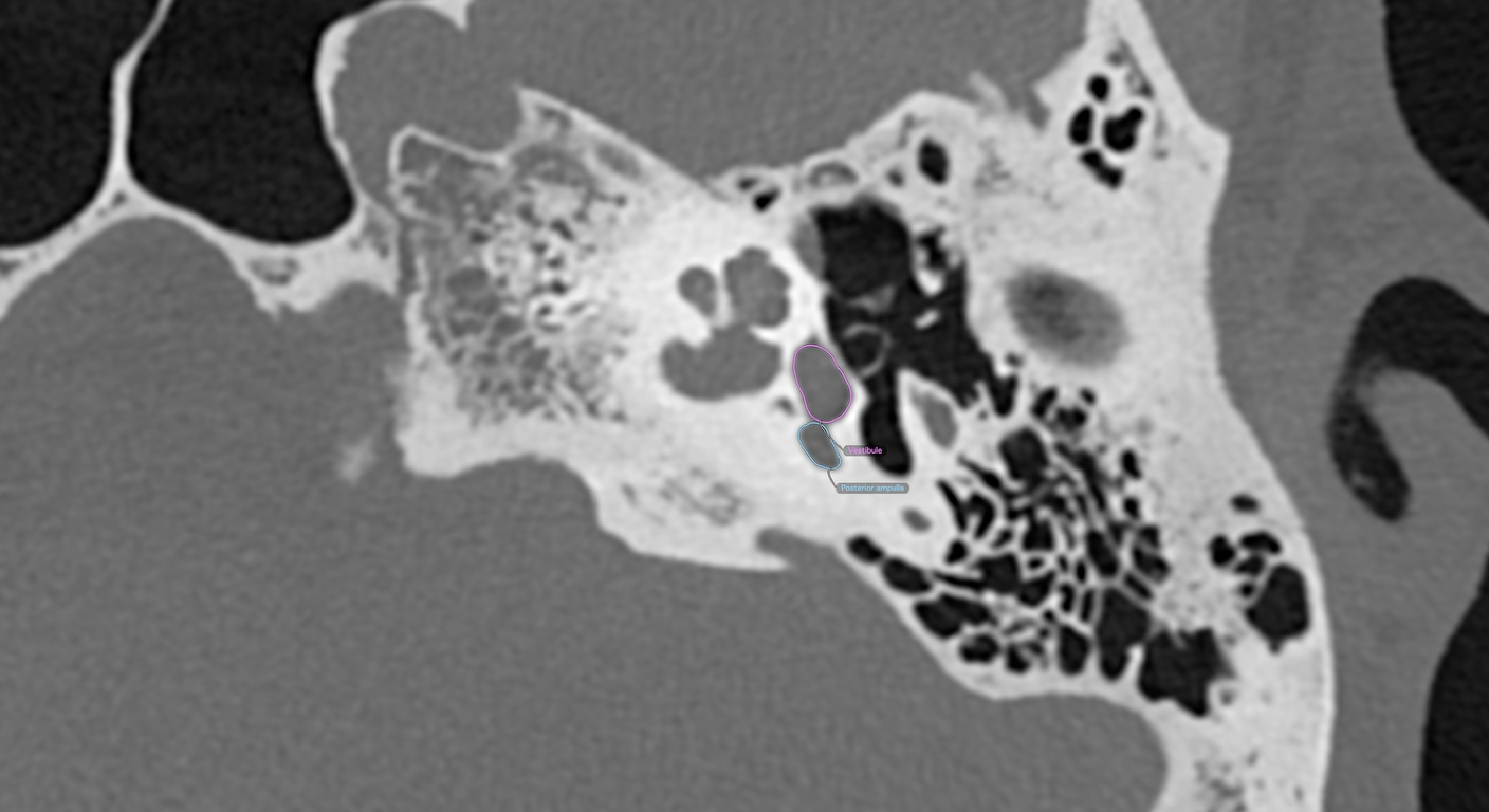
Vestibule
Posterior ampulla

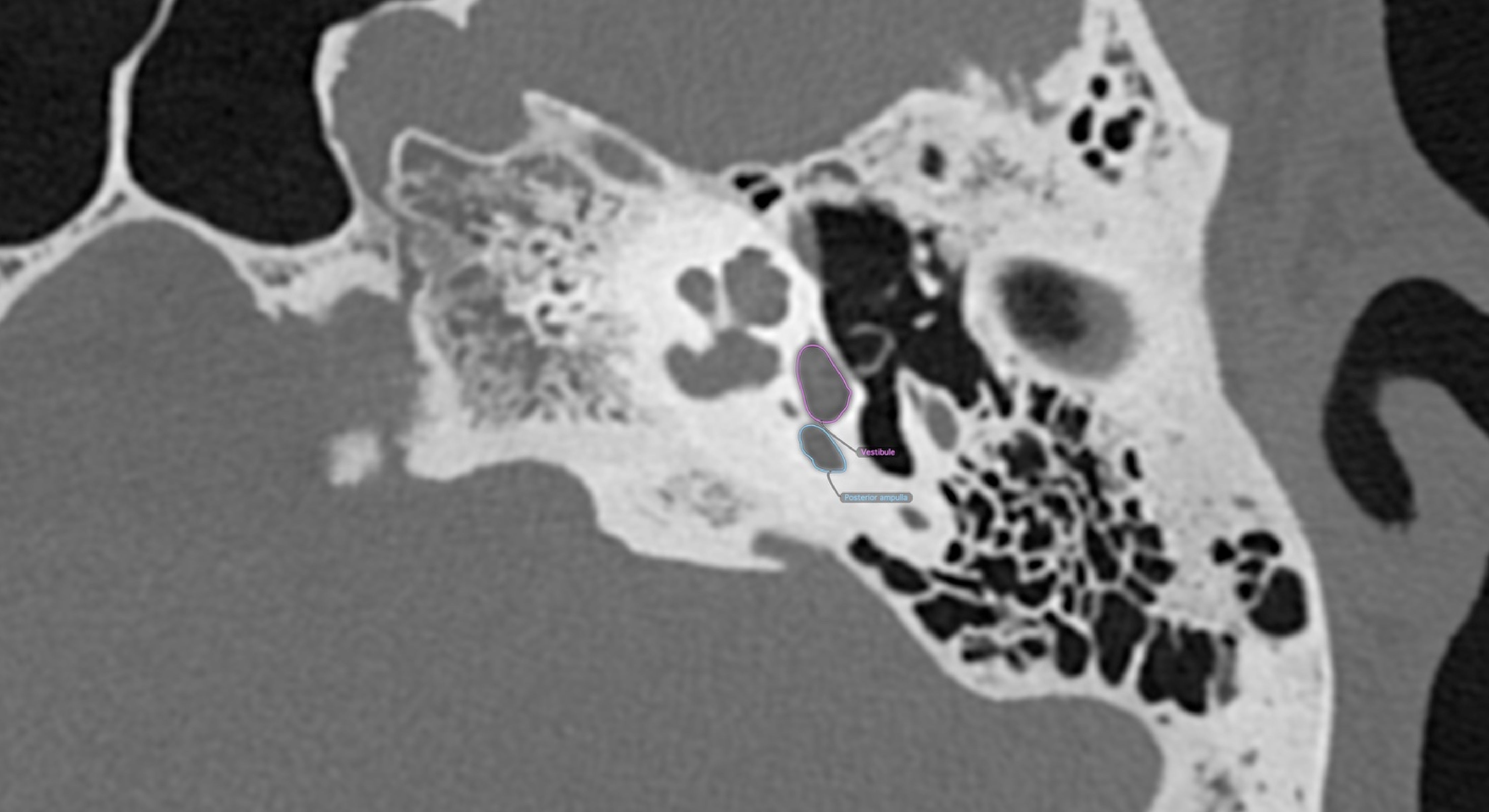

Vestibule
Posterior ampulla

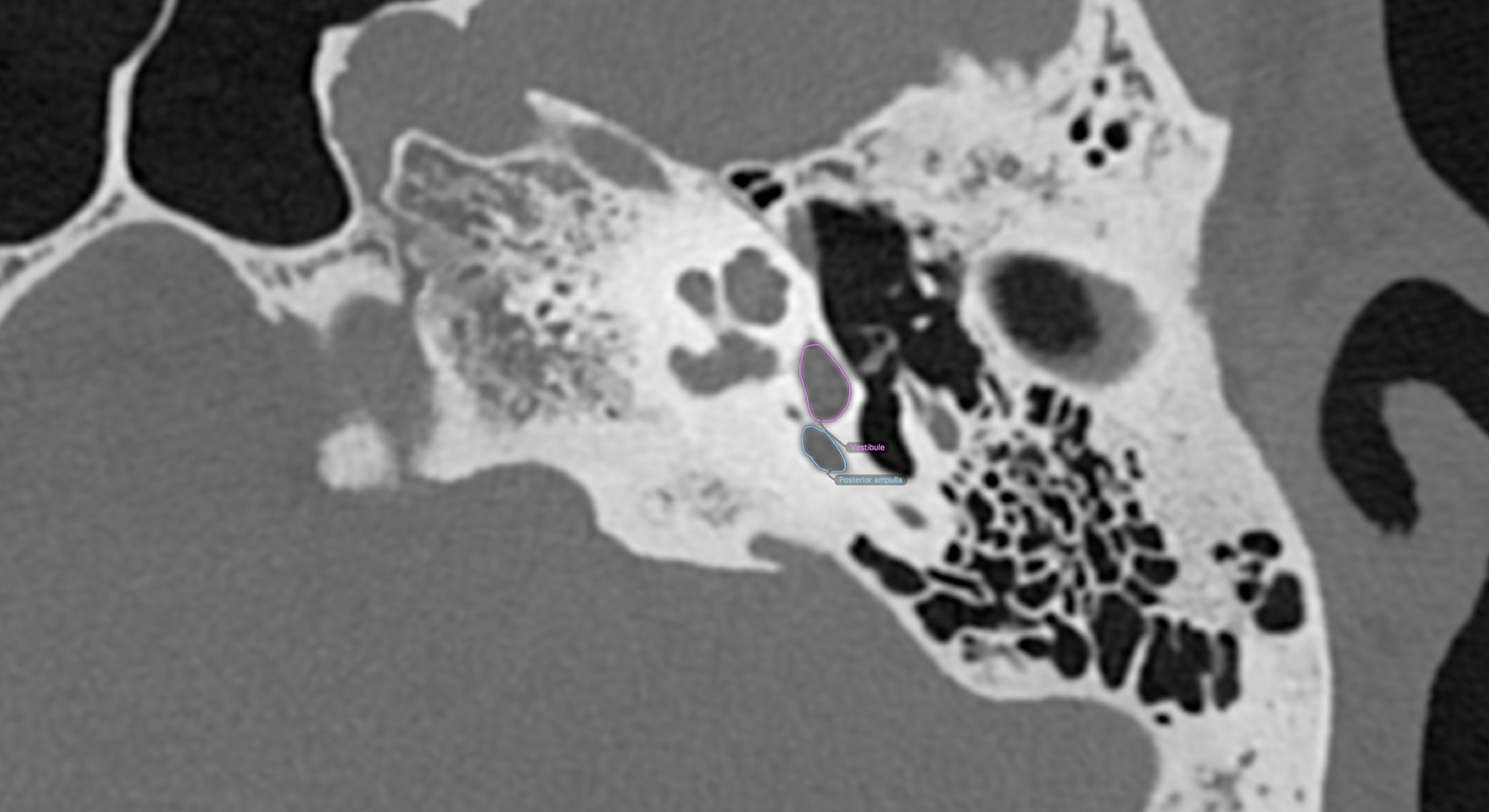
Vestibule
Posterior ampulla

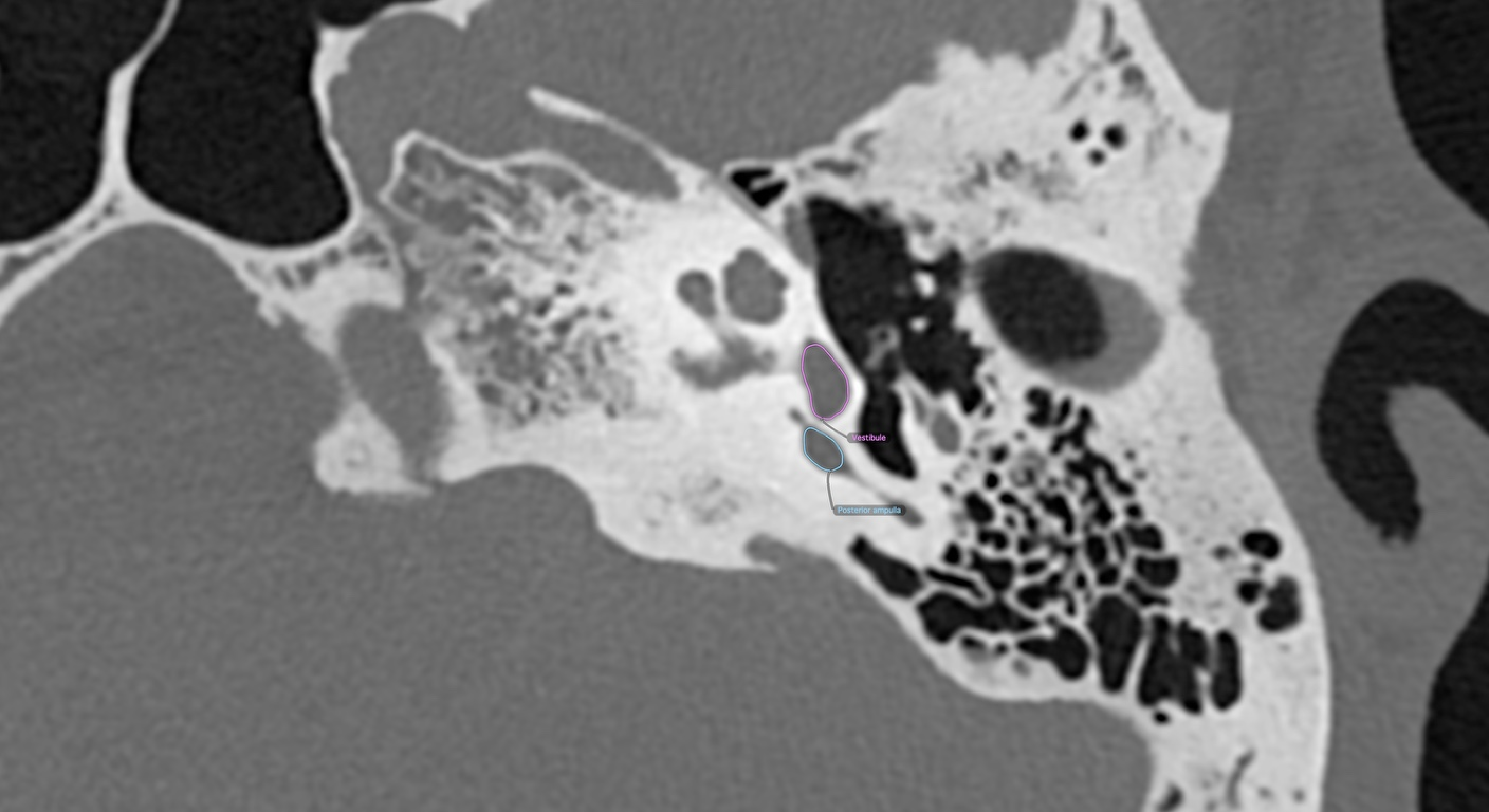
Vestibule
Posterior ampulla

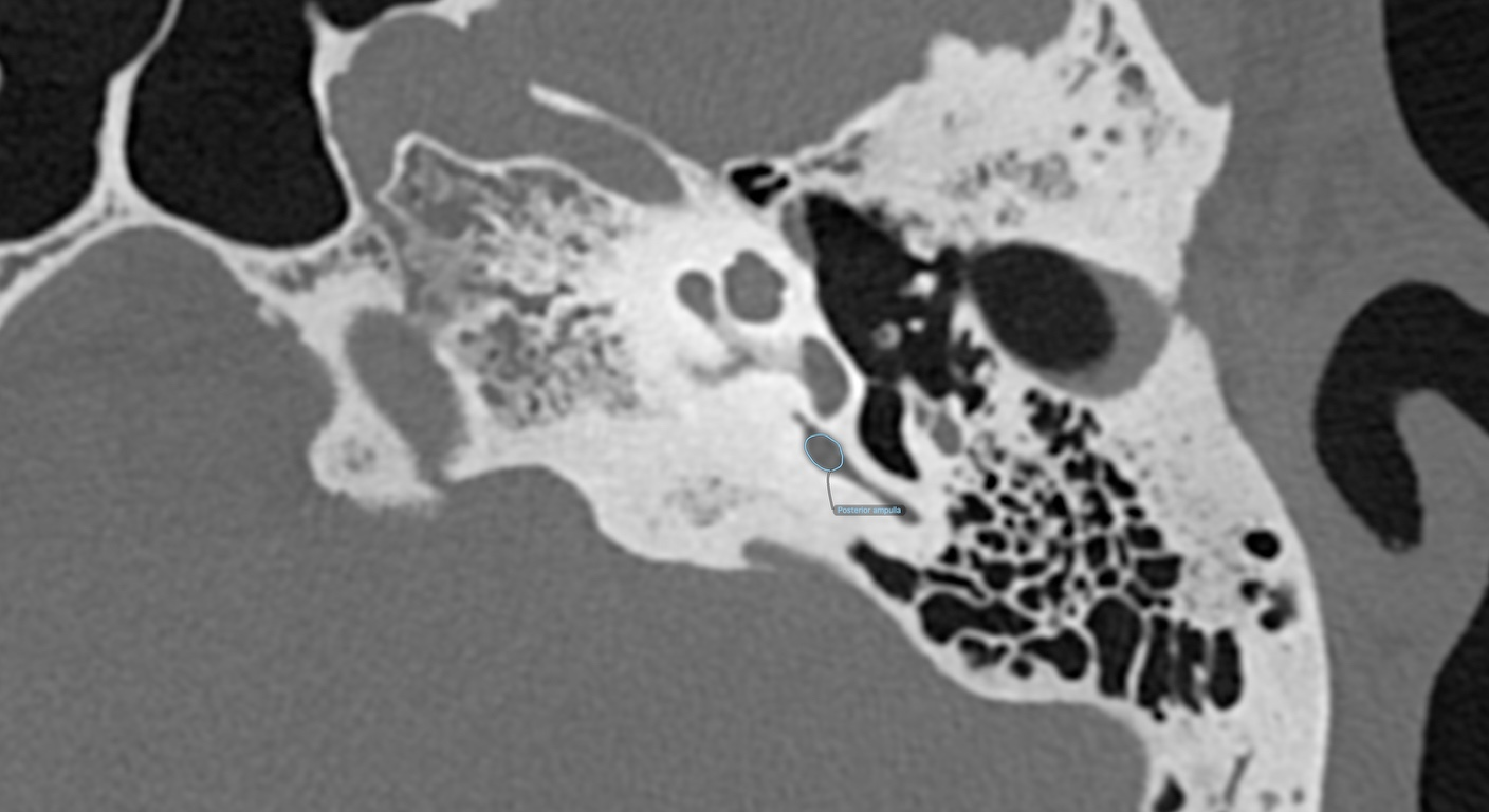
Posterior ampulla

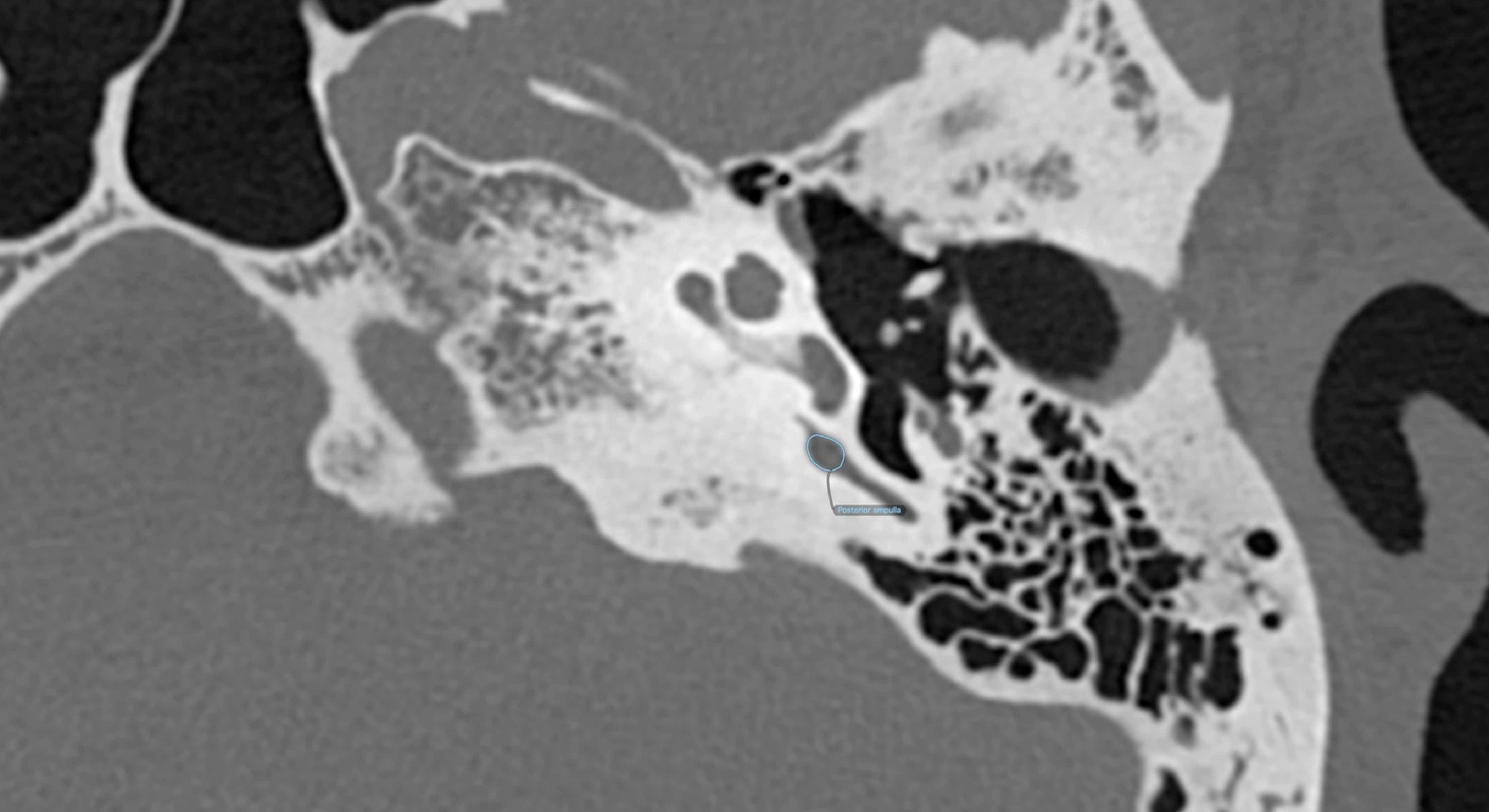
Posterior ampulla

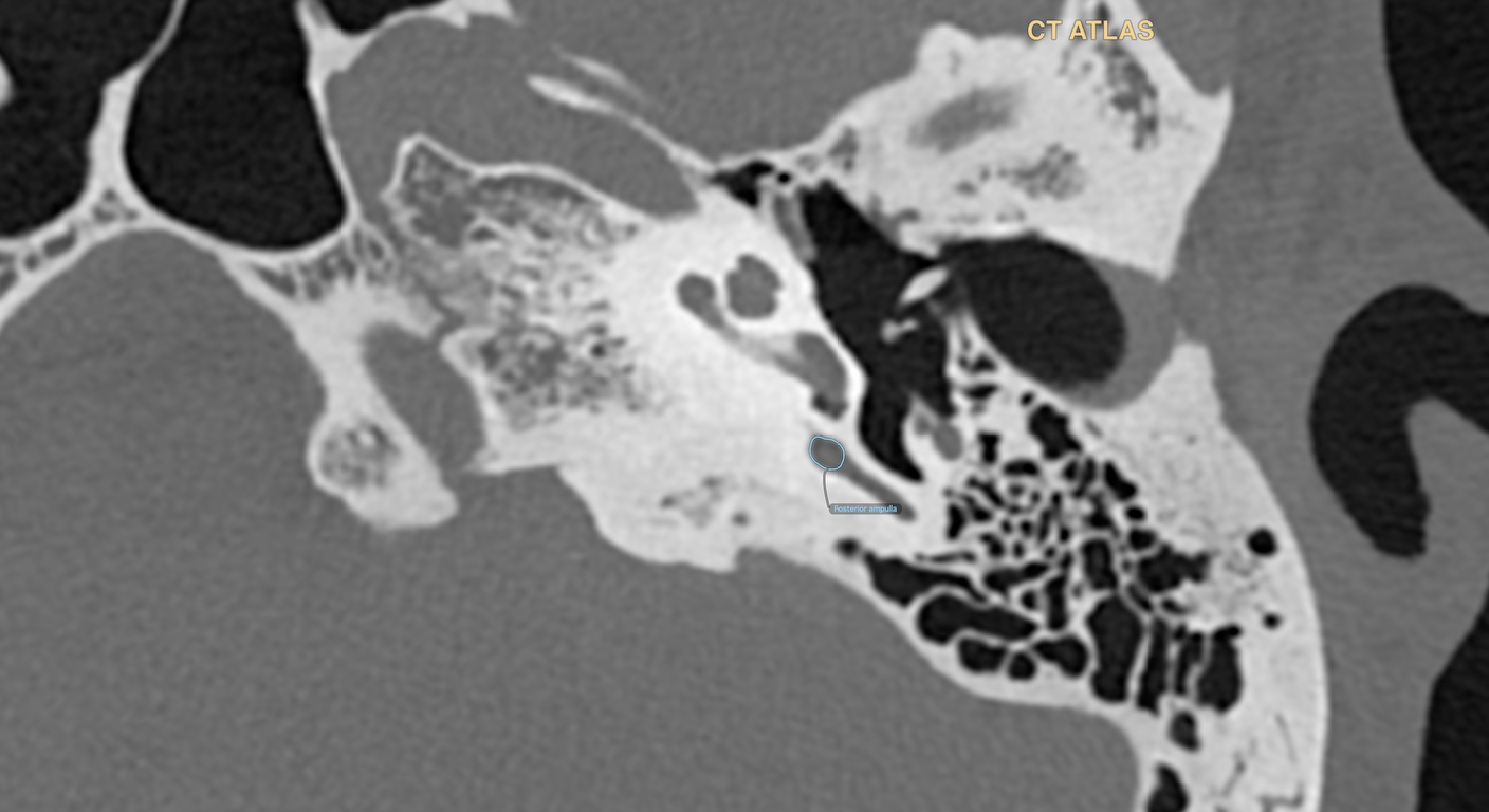
CT ATLAS
Posterior ampulla

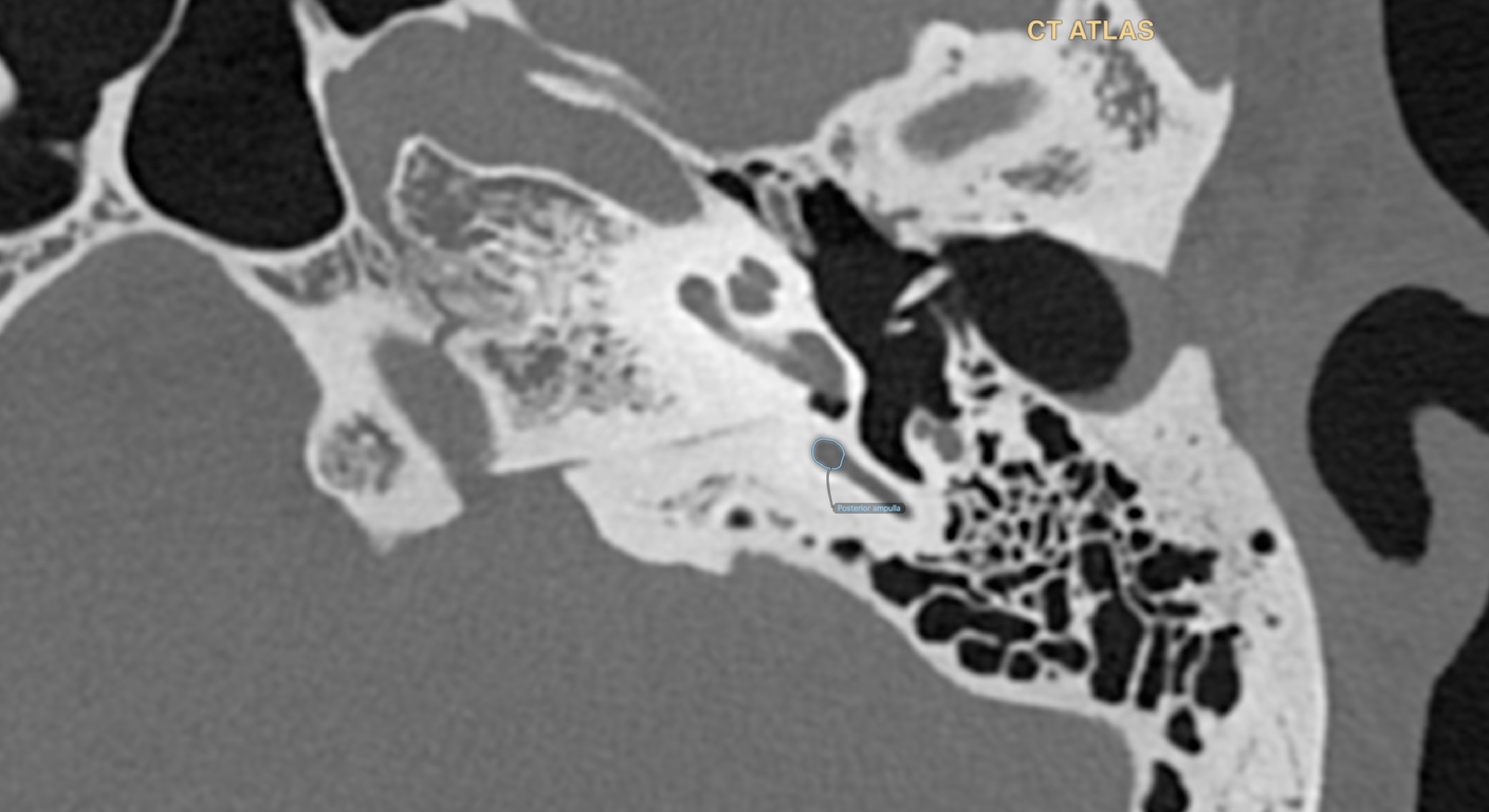
CT ATLAS
Posterior ampulla

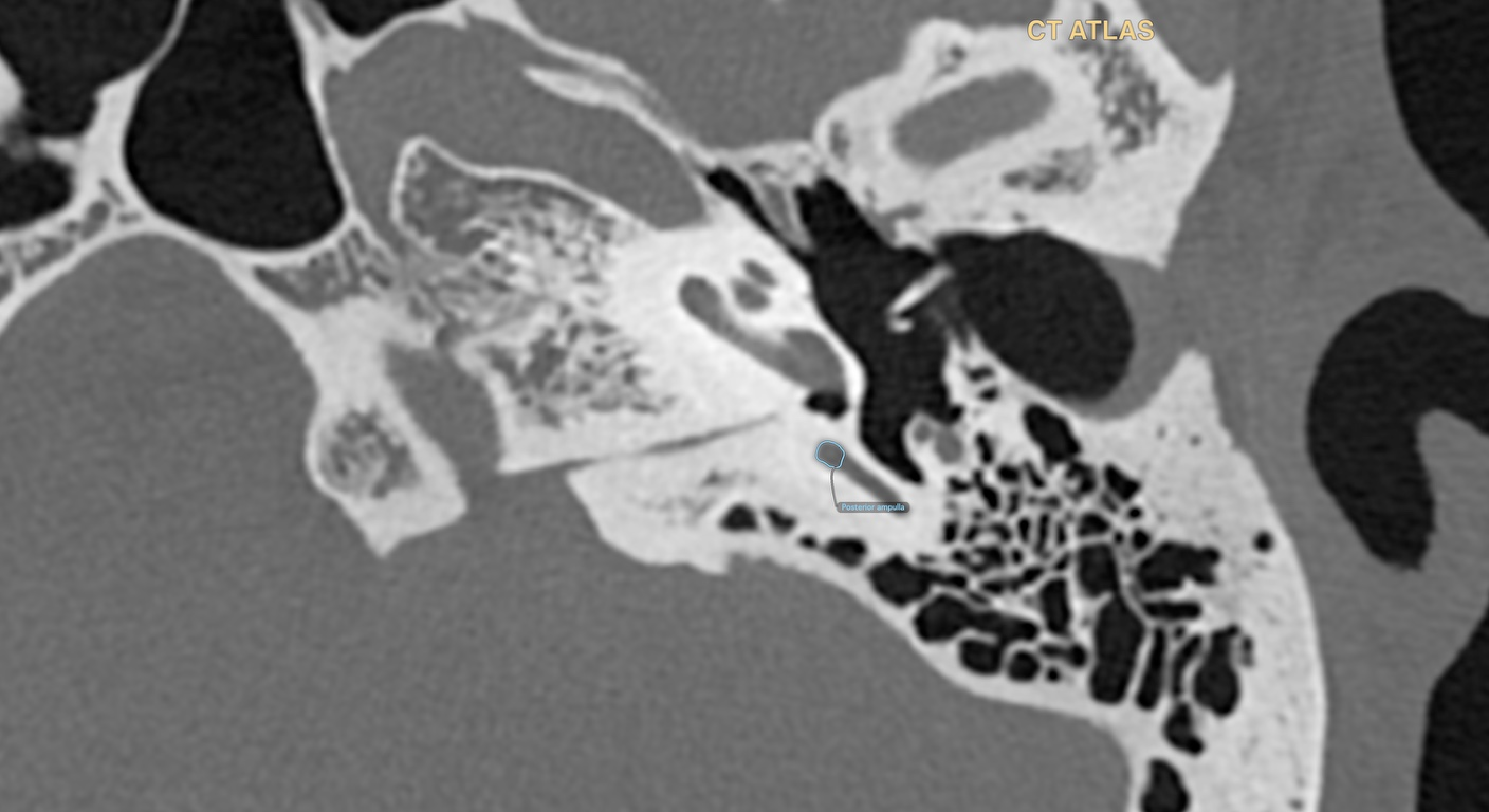
CT ATLAS
Posterior ampulla

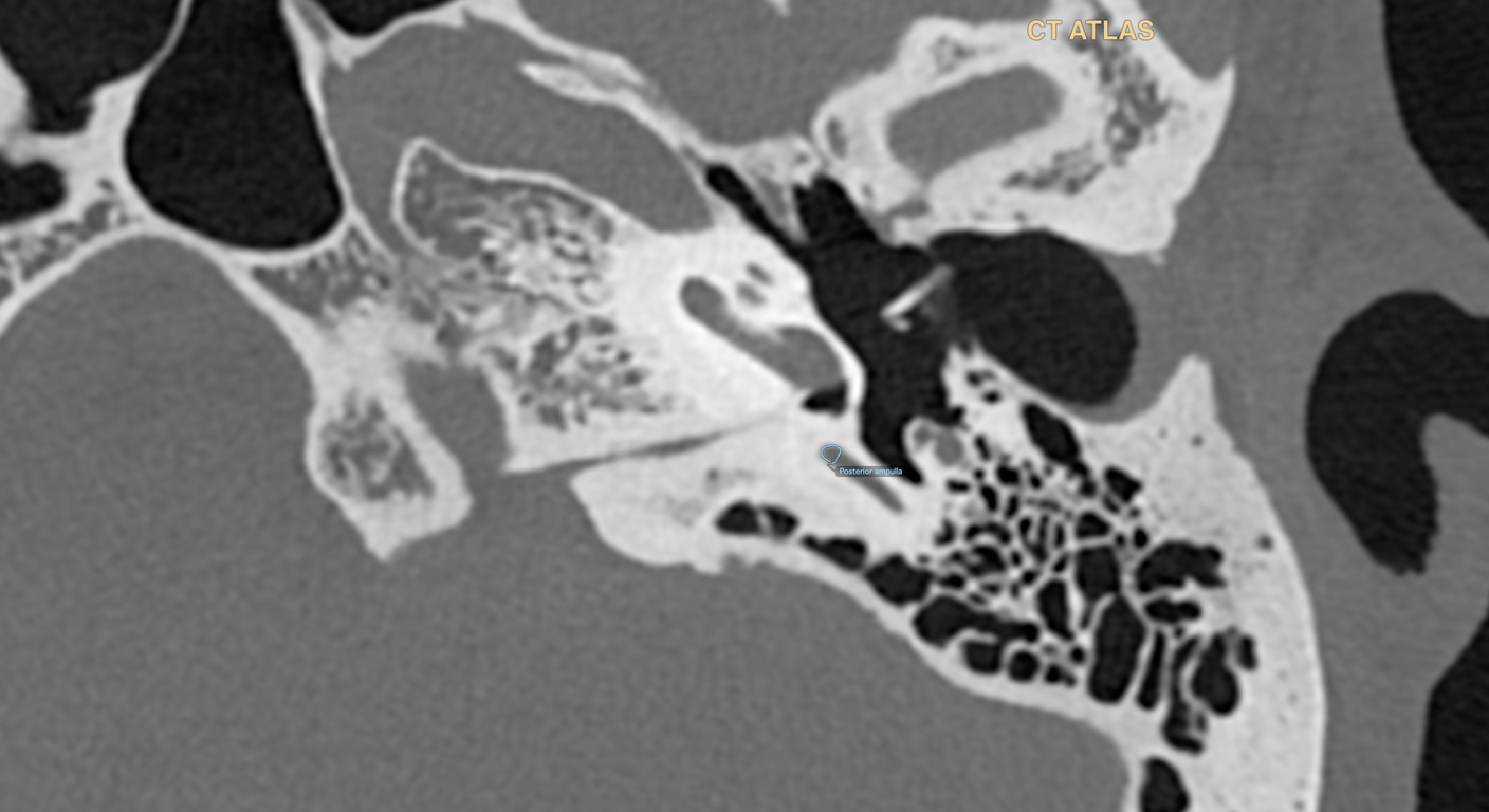
CT ATLAS
Posterior ampulla

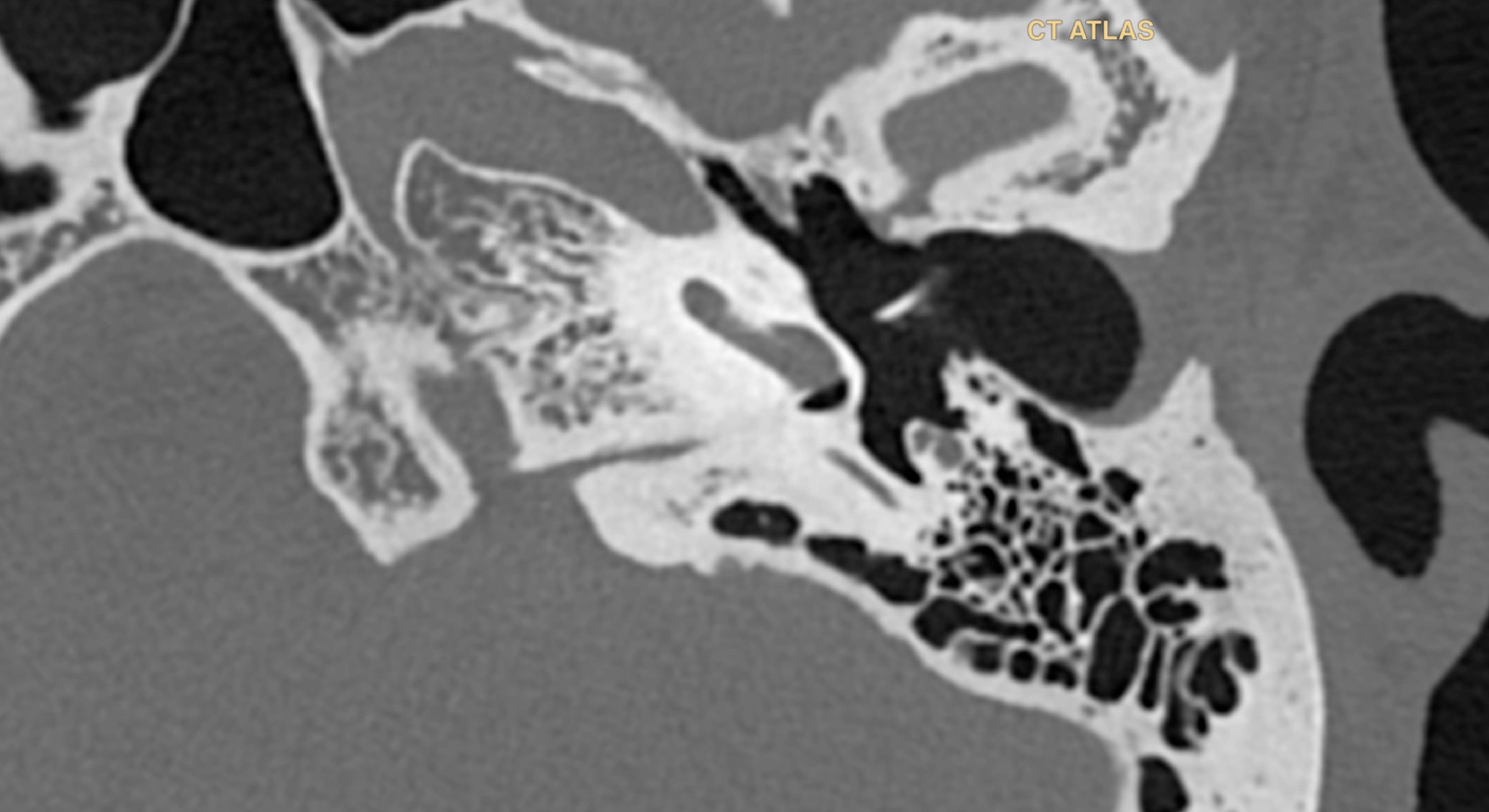
CT ATLAS

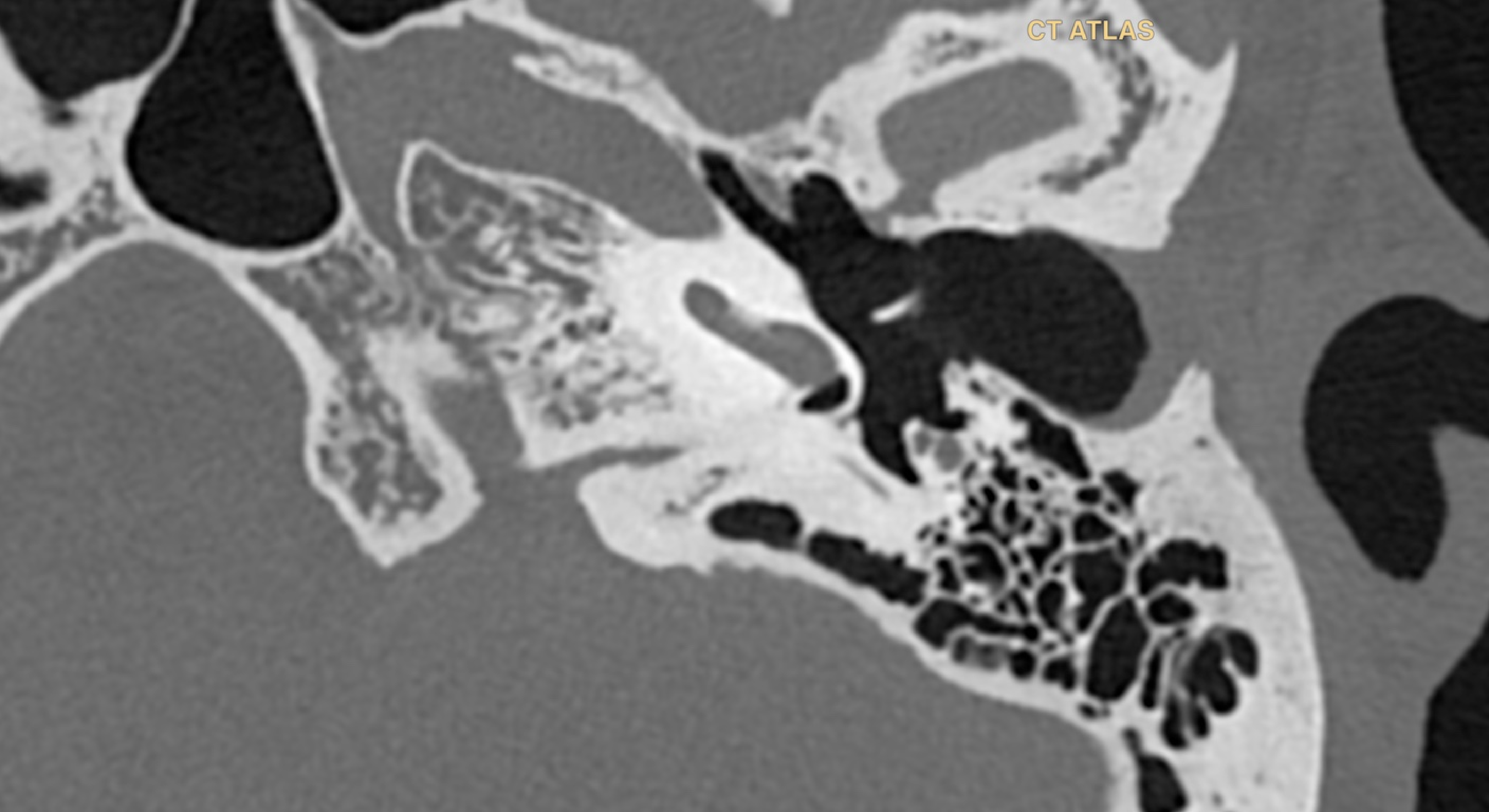
CT ATLAS

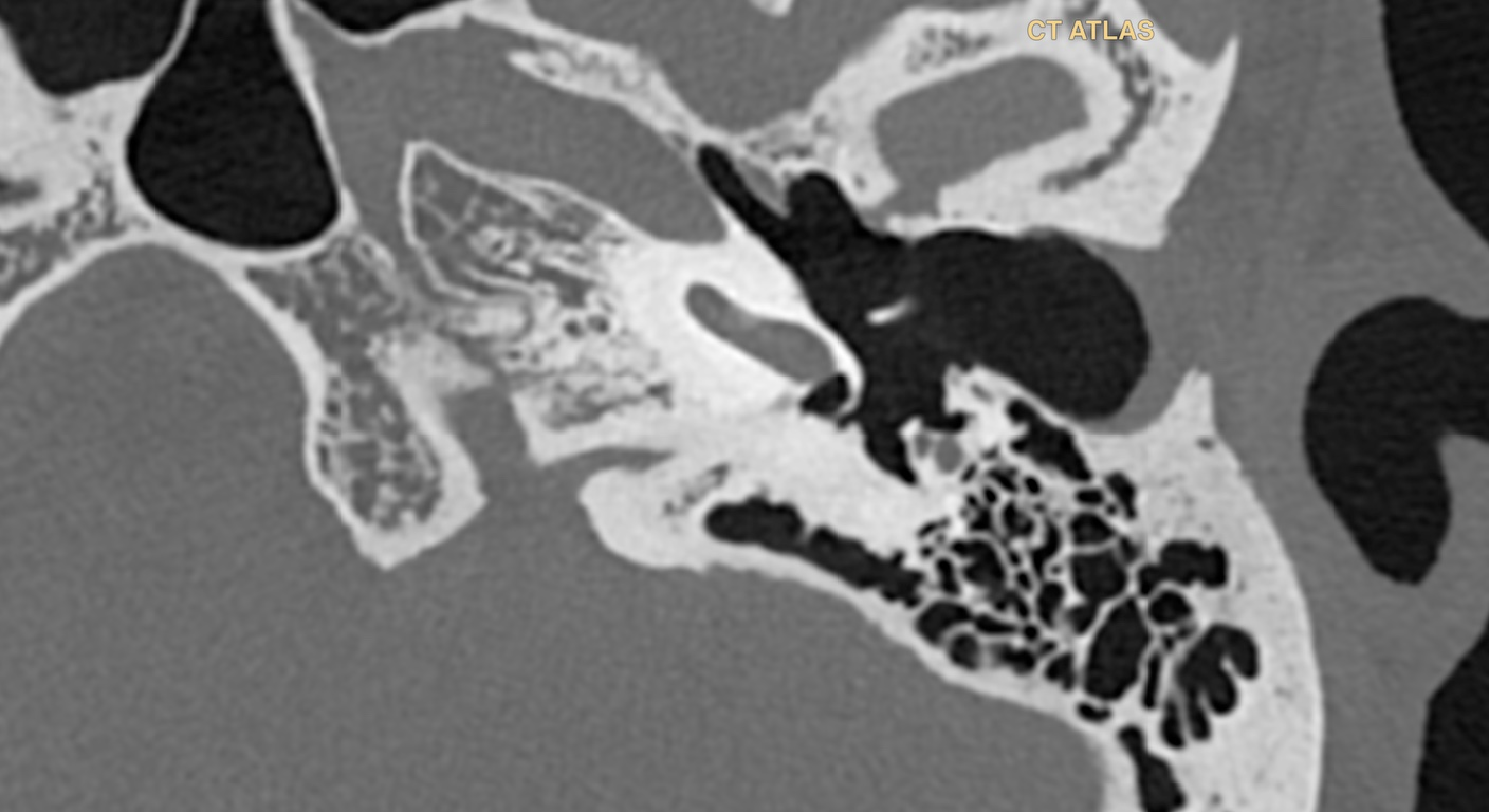
CT ATLAS

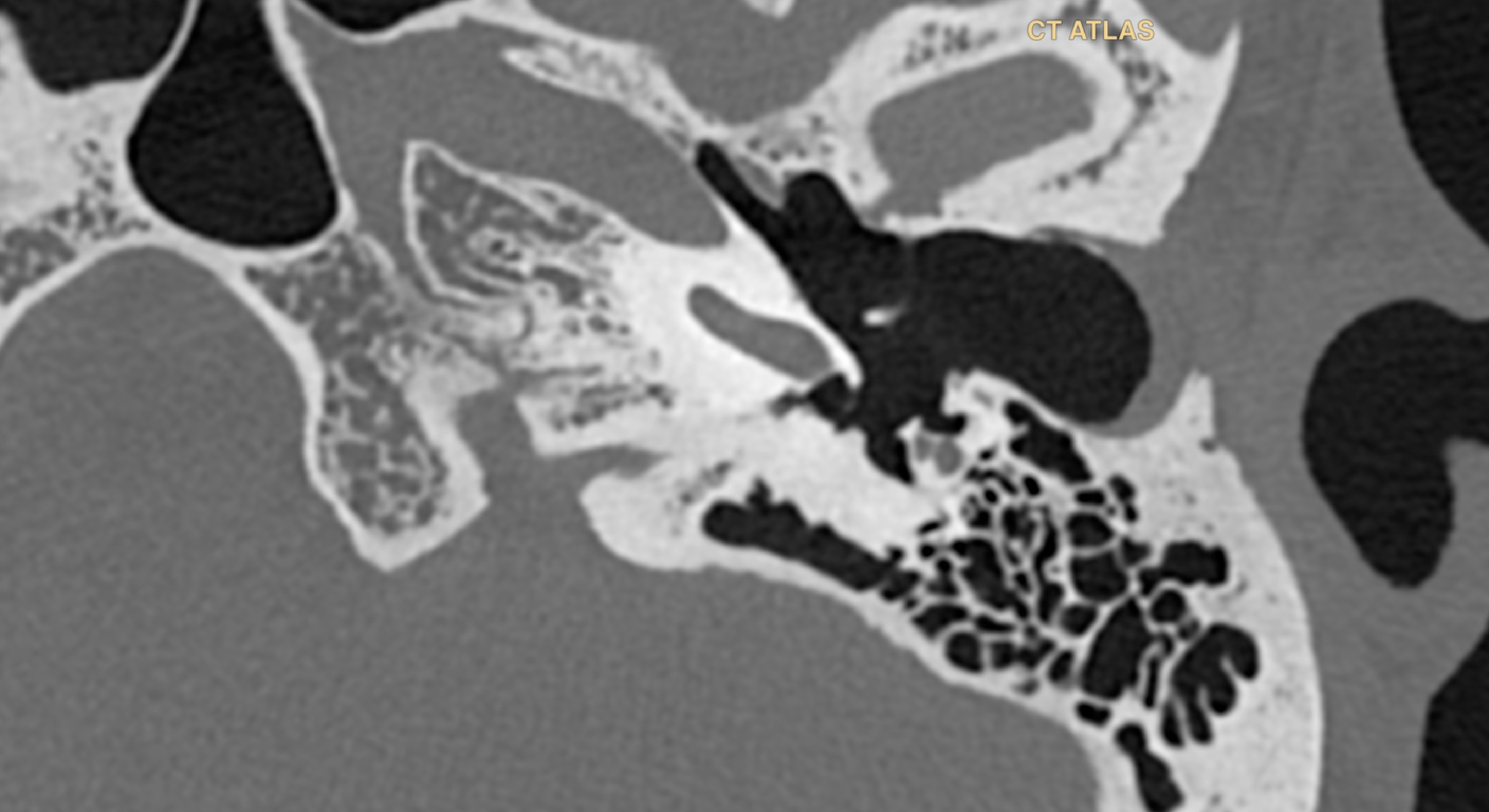
CT ATLAS

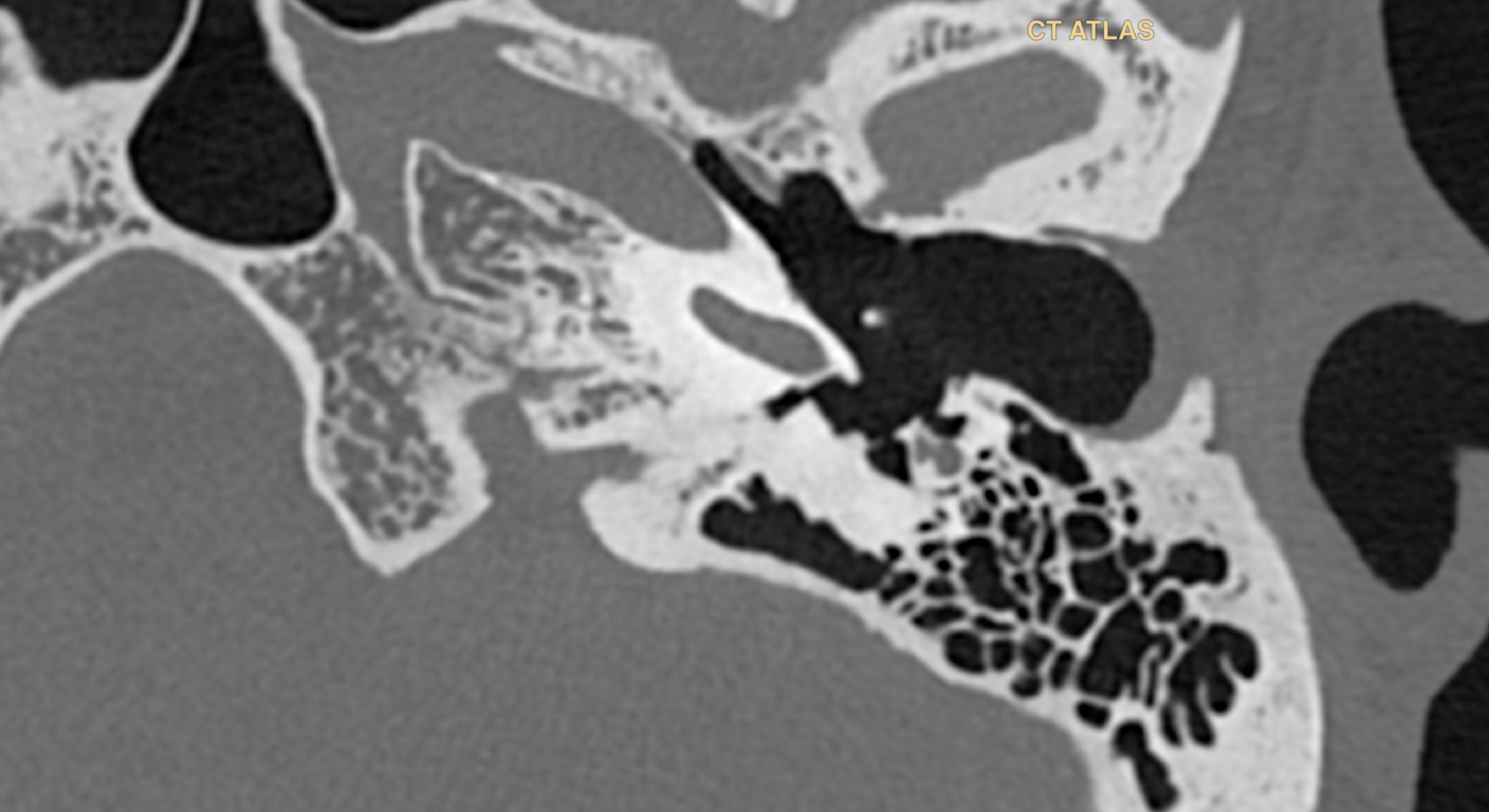
CT ATLAS

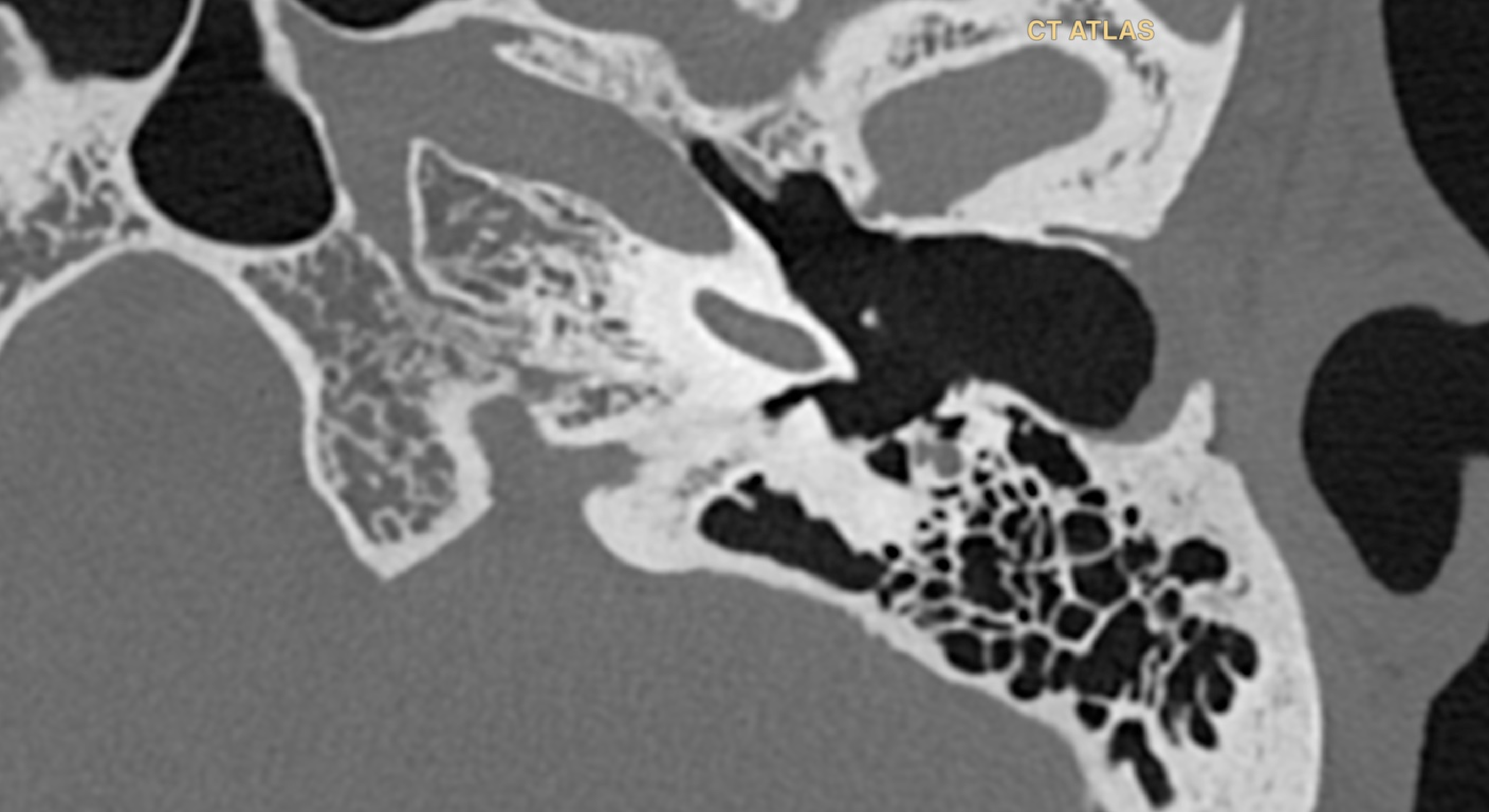
CT ATLAS

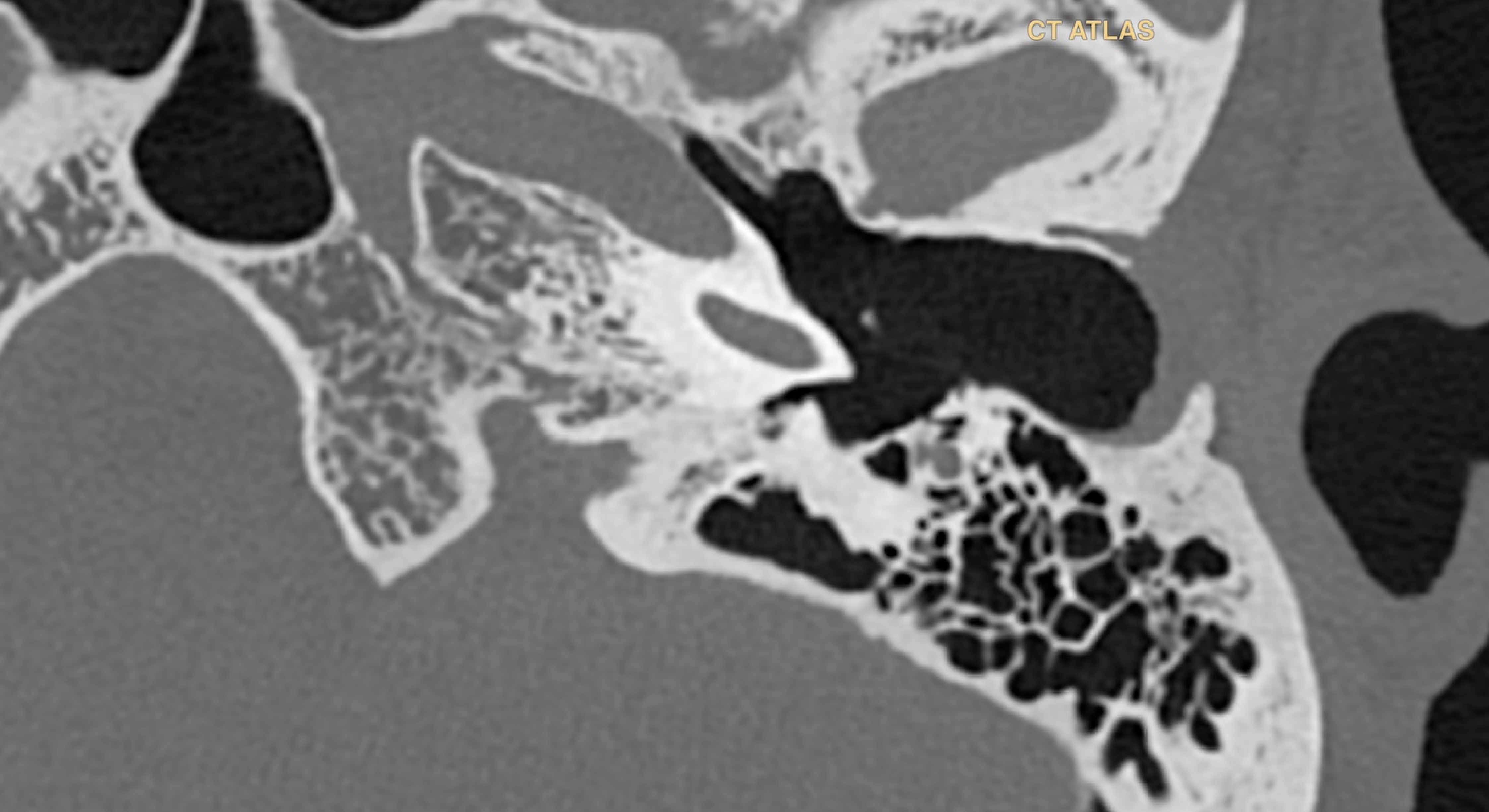
CT ATLAS

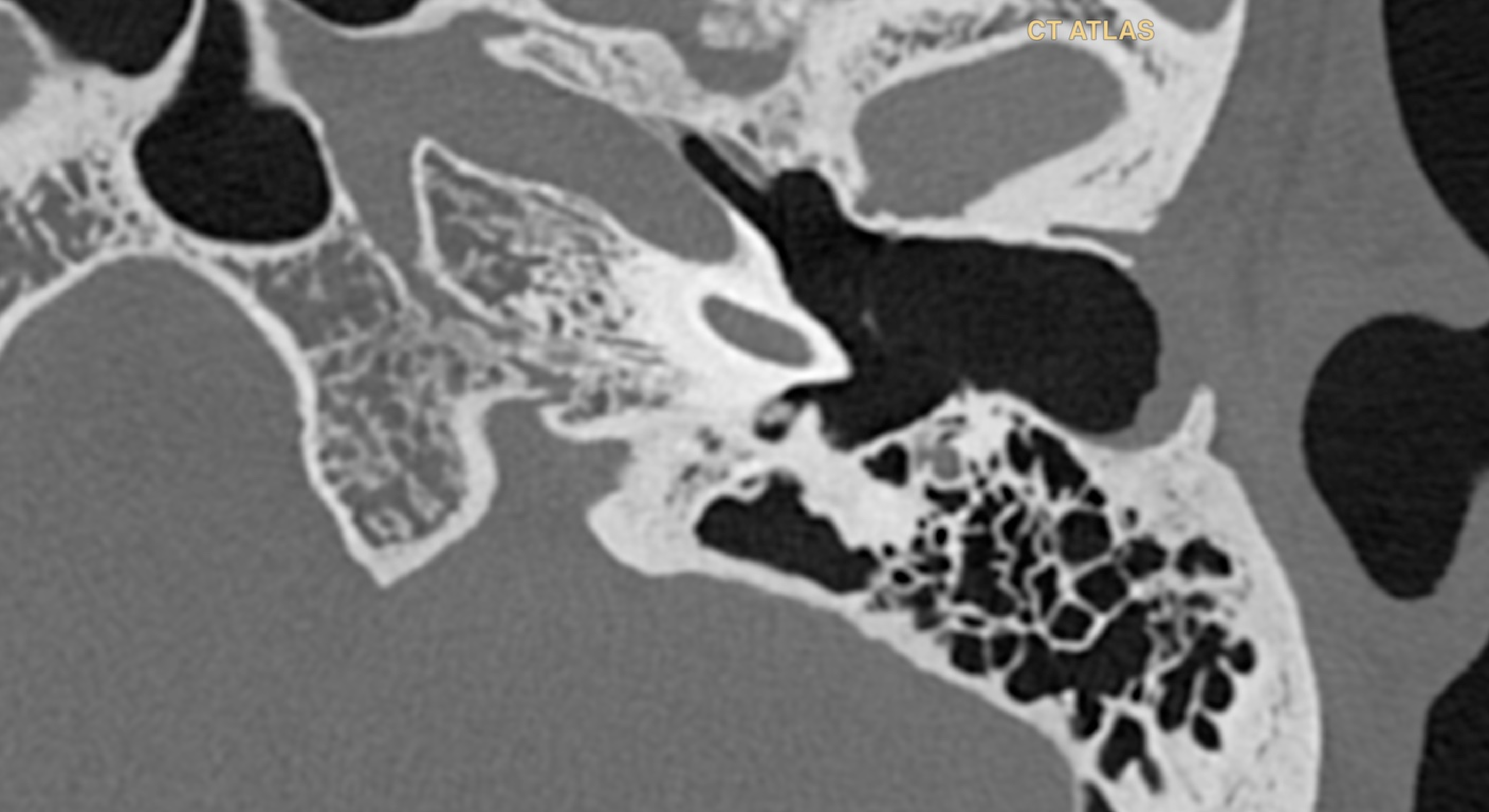
CT ATLAS

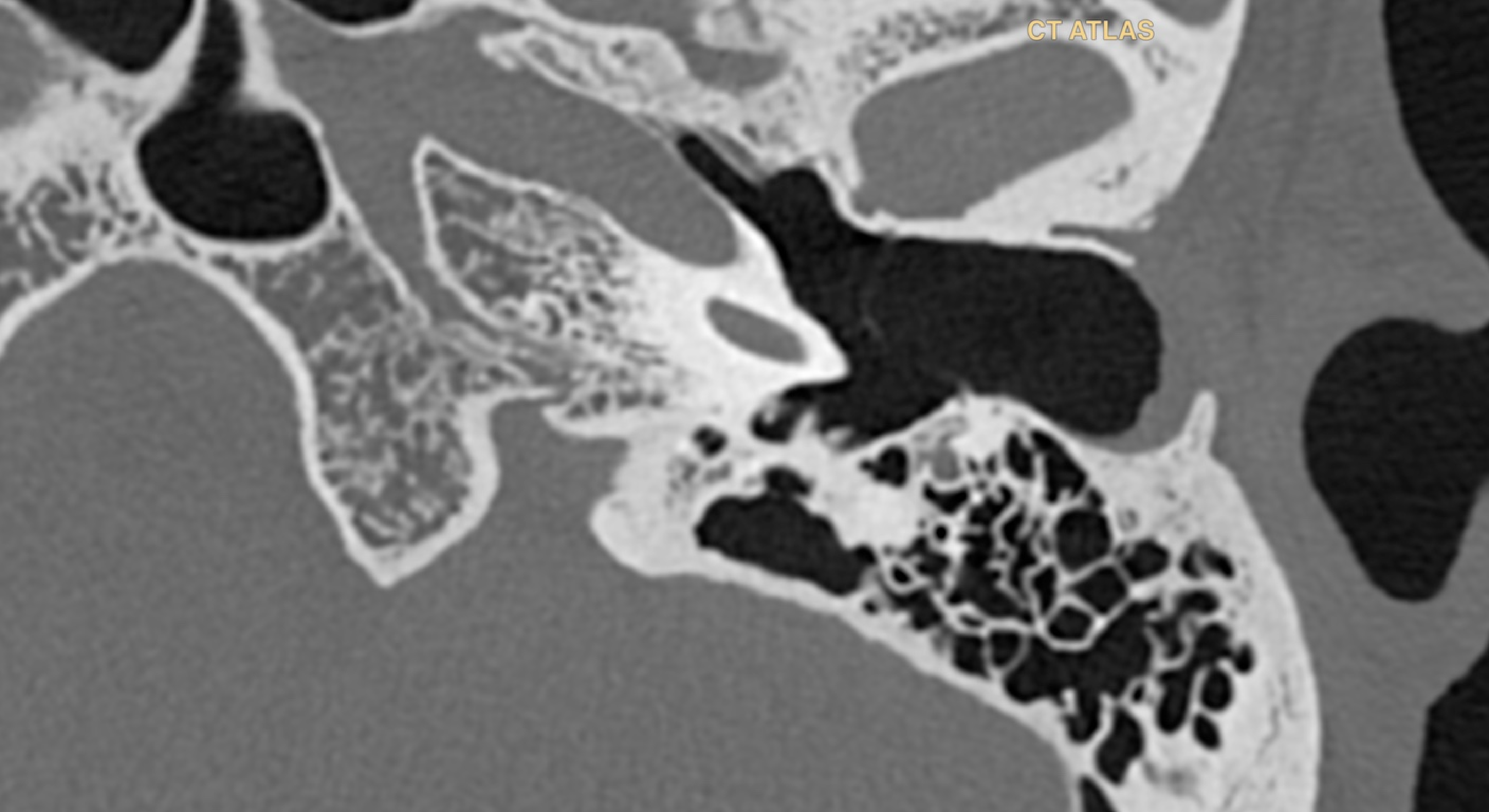
CT ATLAS

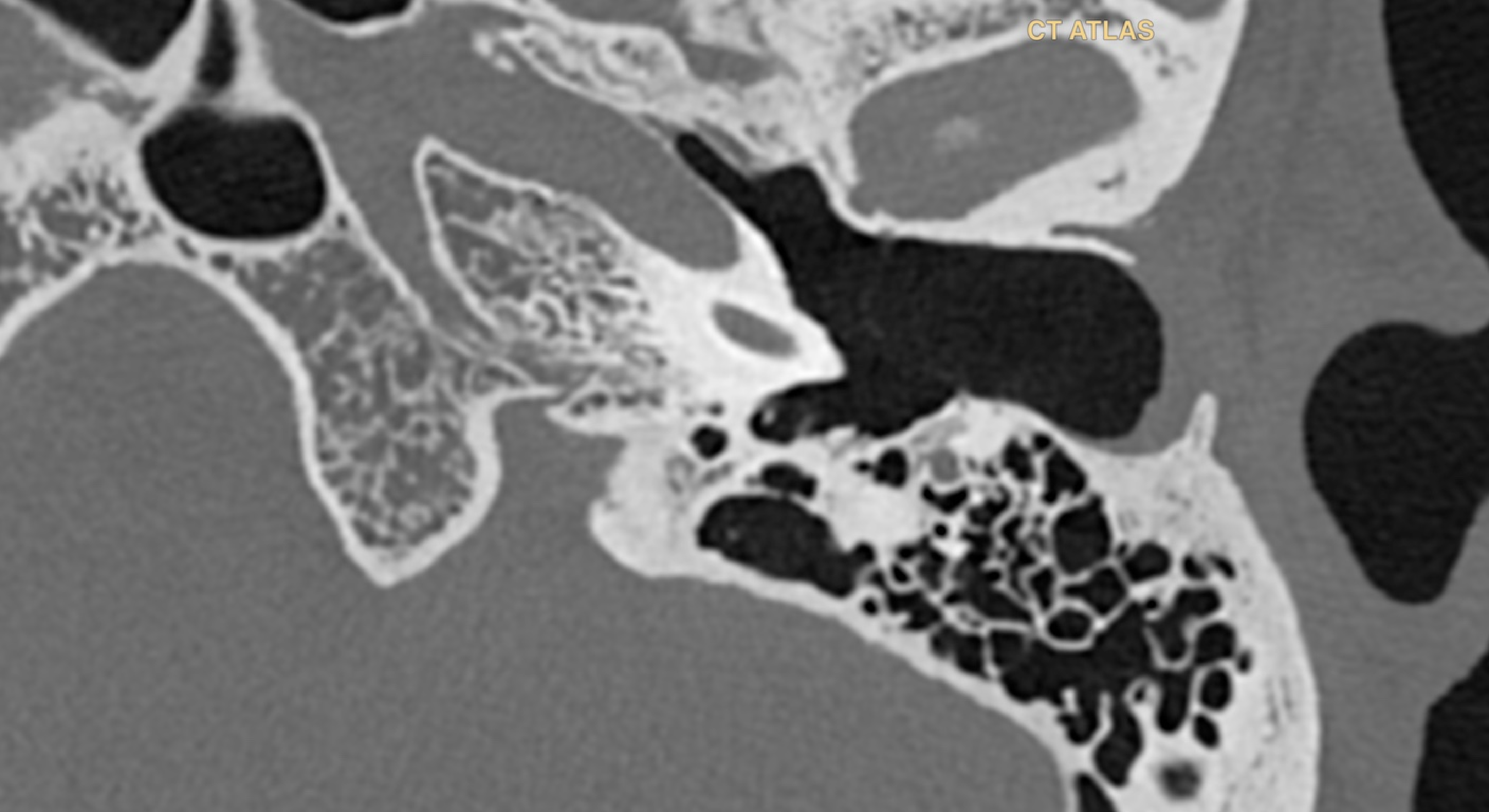
CT ATLAS

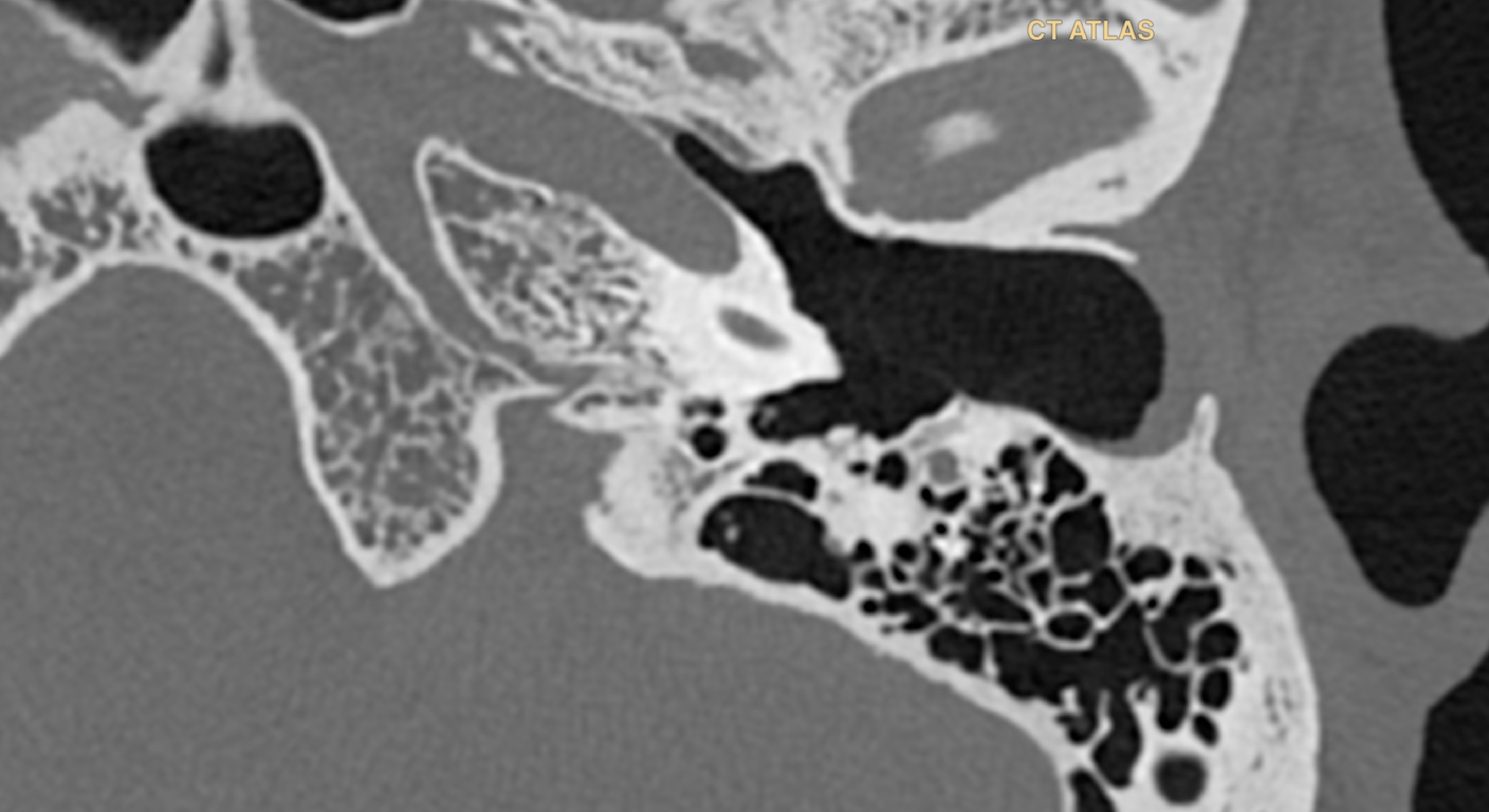
CT ATLAS

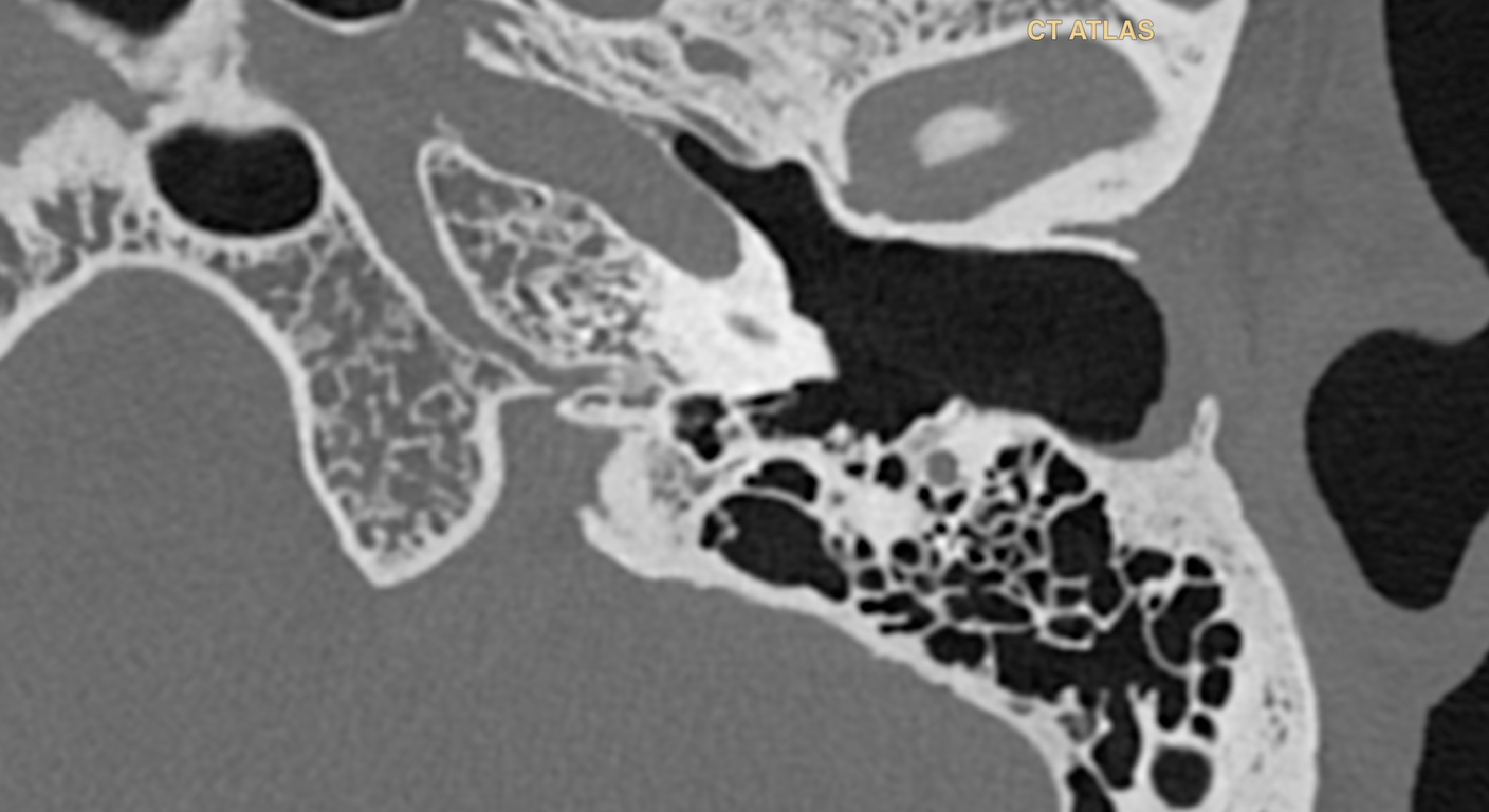
CT ATLAS

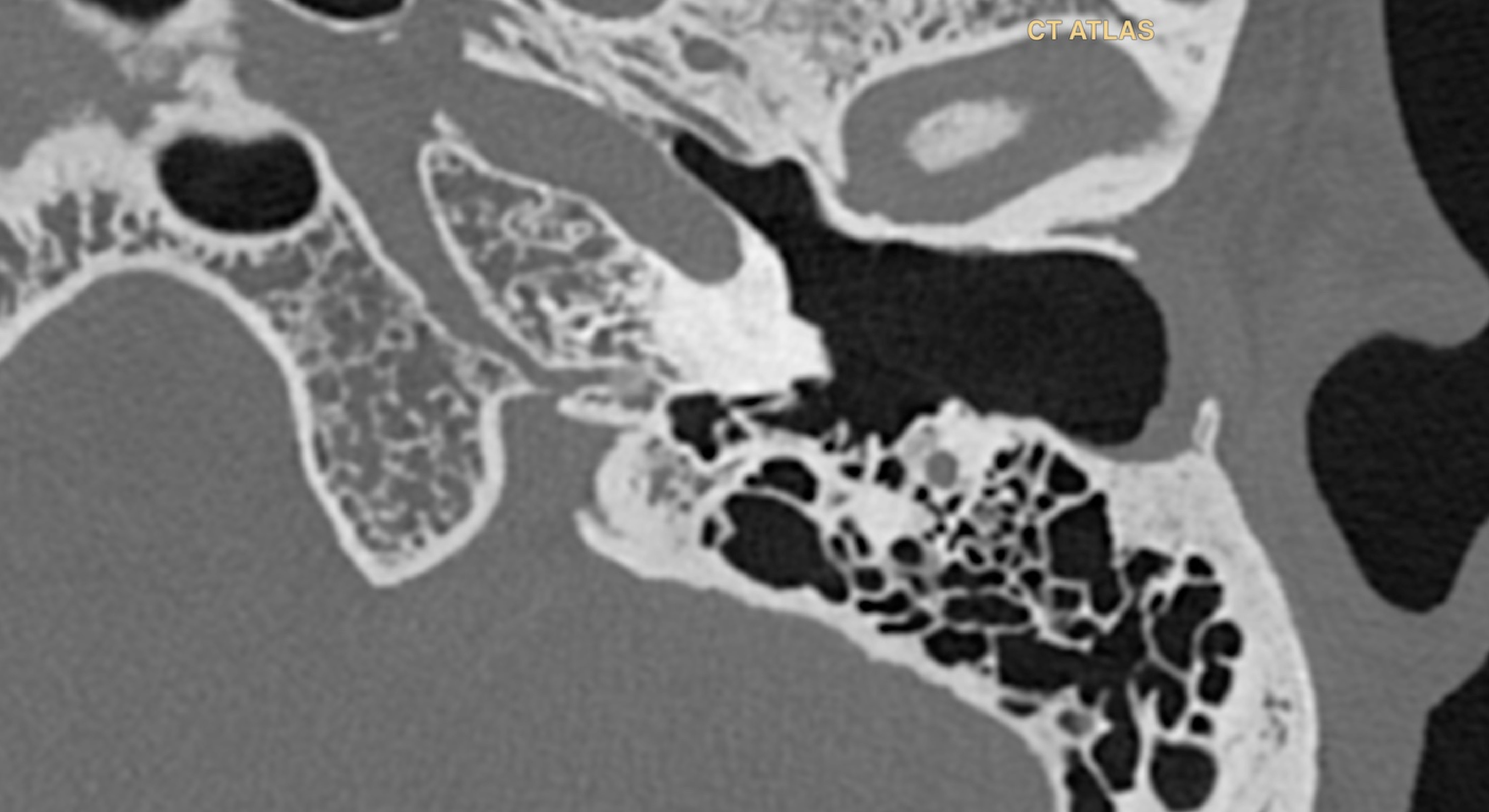
CT ATLAS

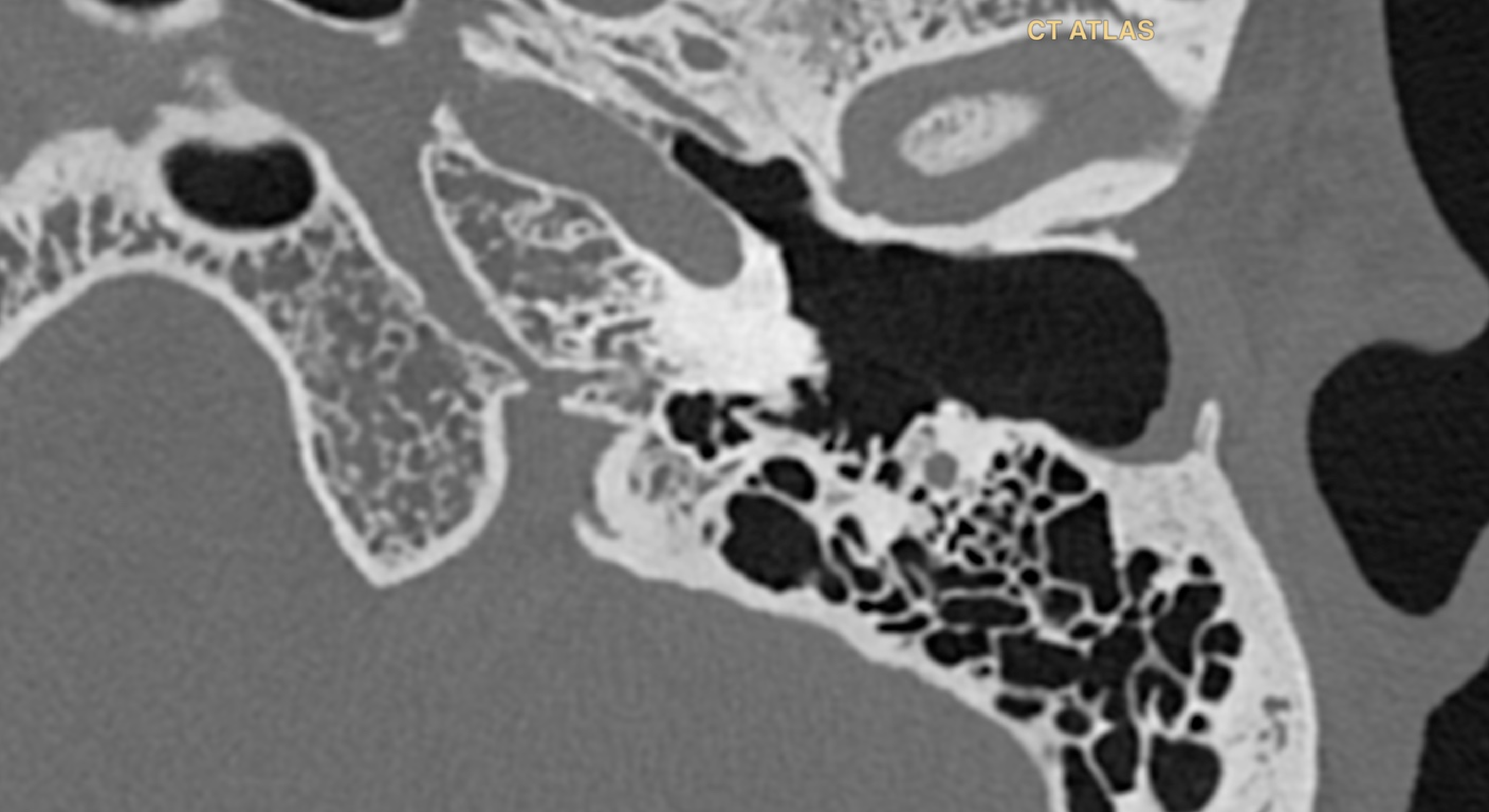
CT ATLAS

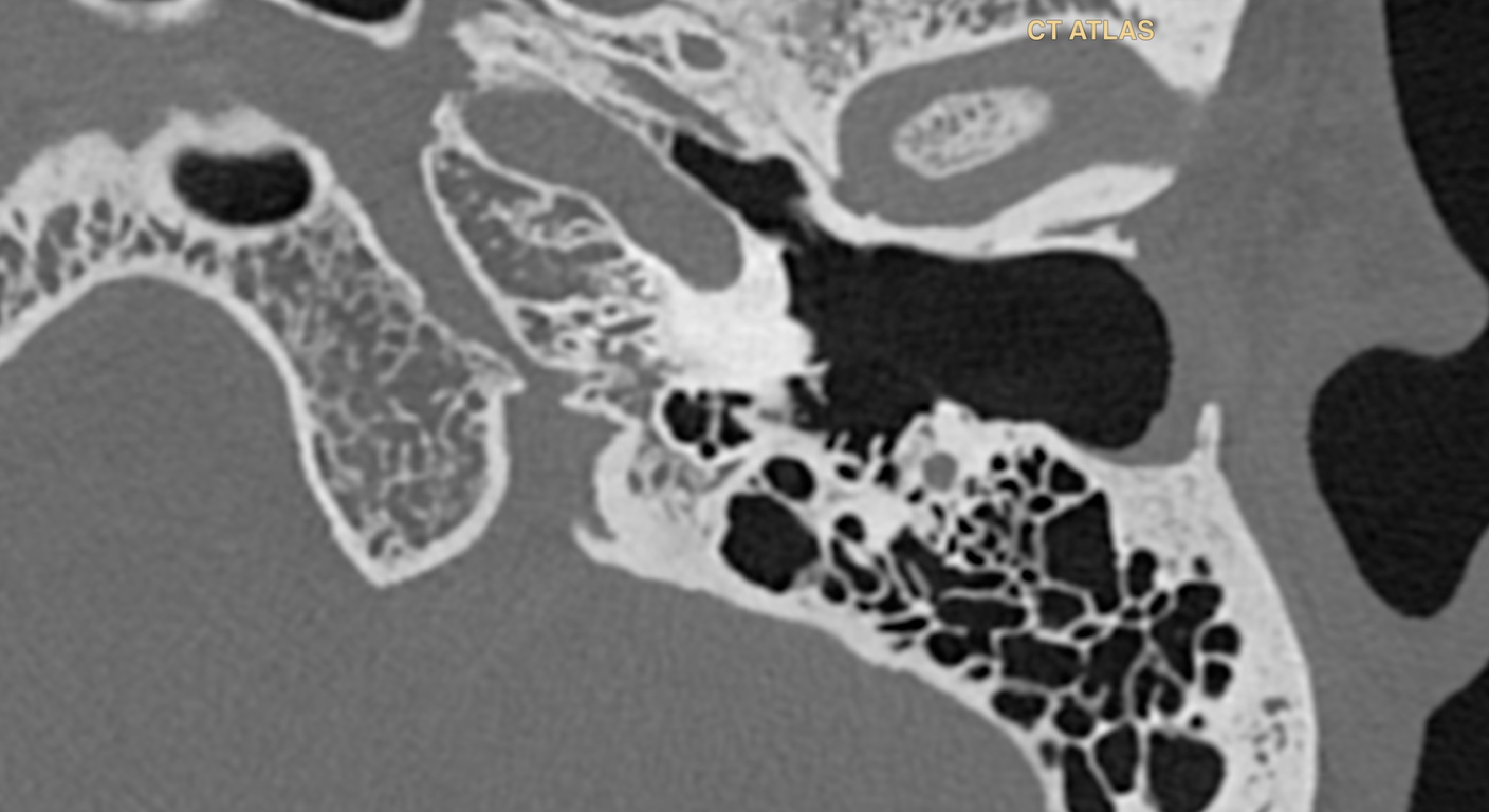
CT ATLAS

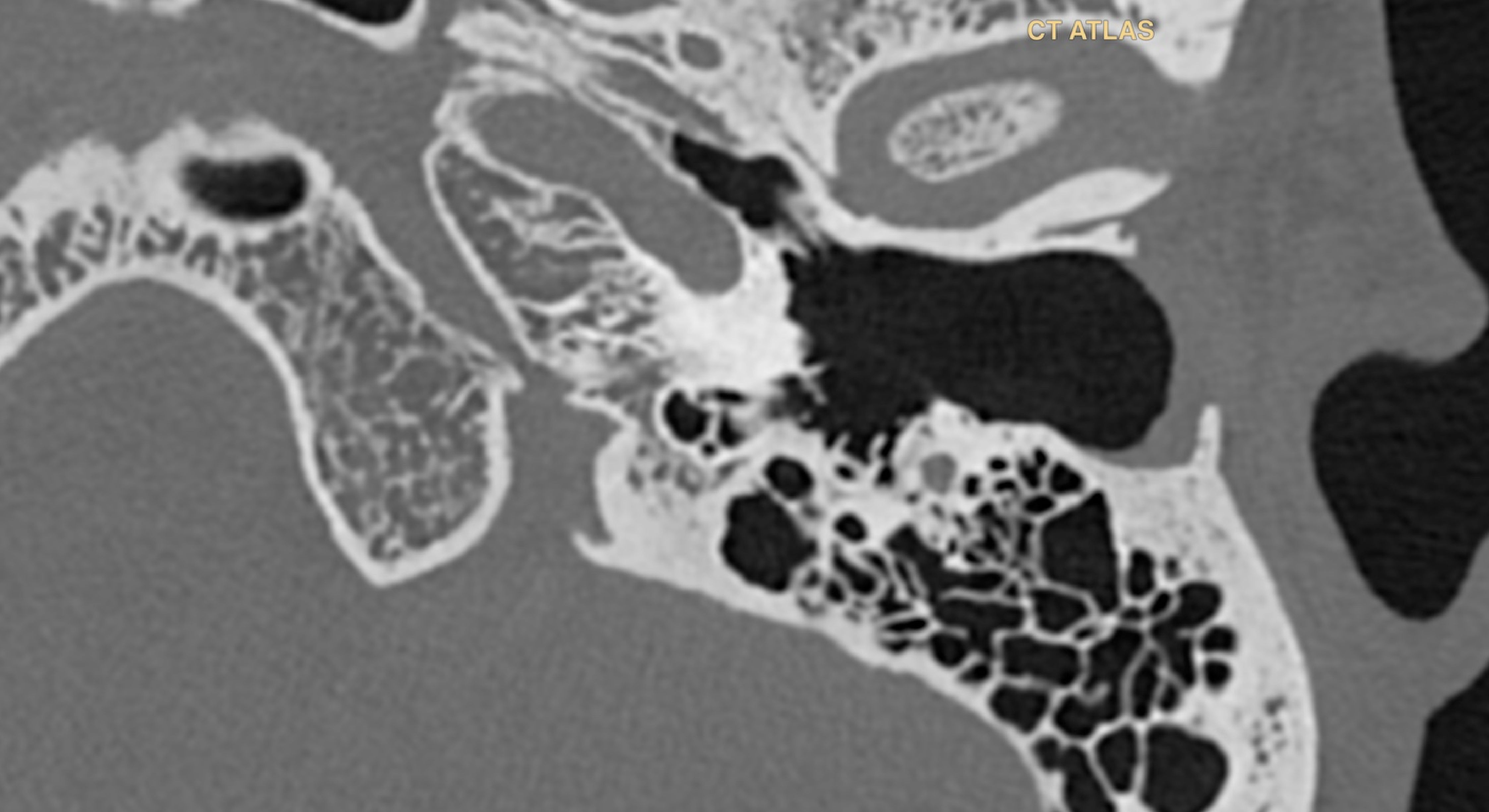
CT ATLAS

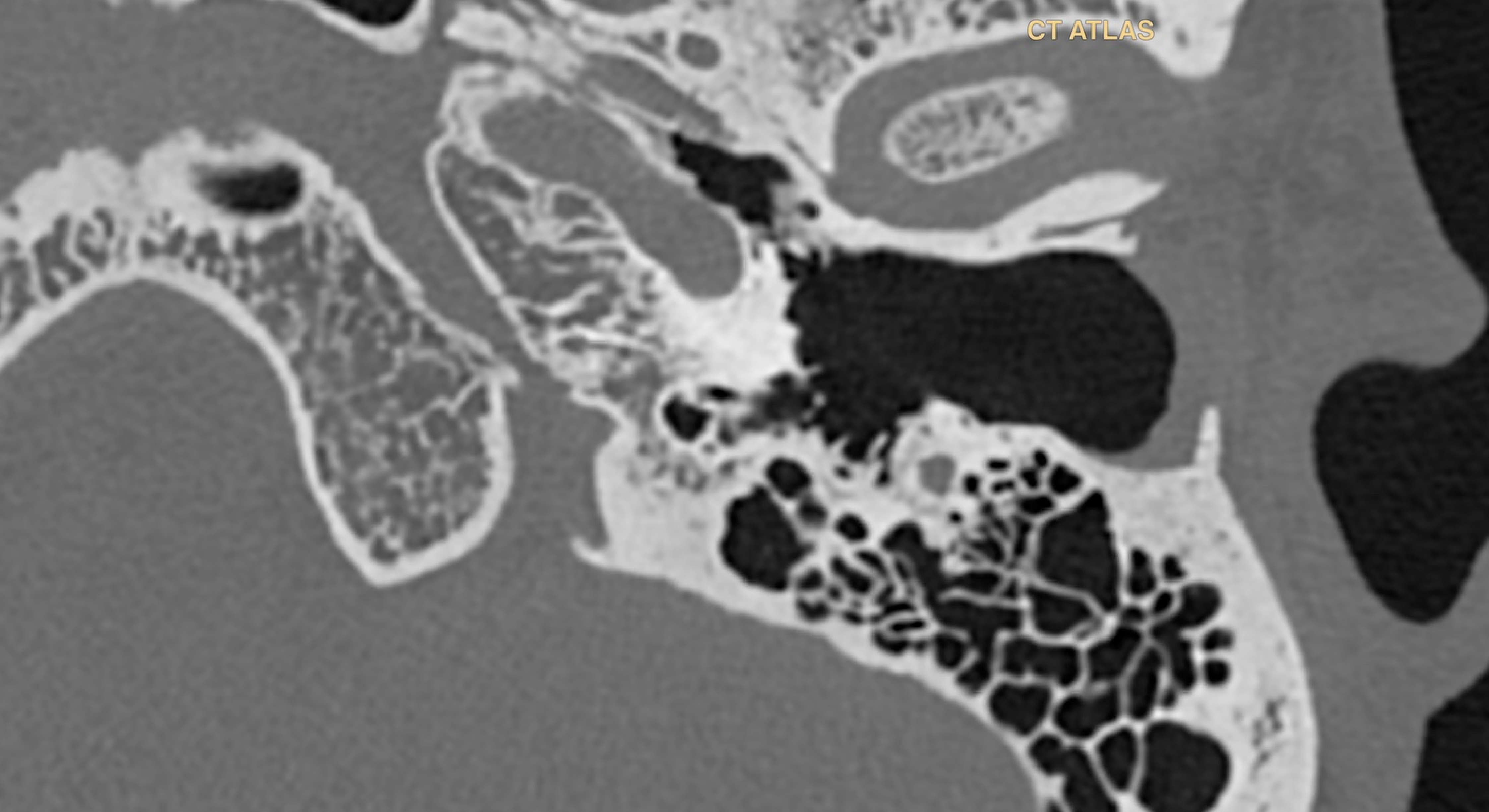
CT ATLAS
